# Chromatin Controls DNA Replication Origin Selection, Lagging-Strand Synthesis, and Replication Fork Rates

**DOI:** 10.1016/j.molcel.2016.11.016

**Published:** 2017-01-05

**Authors:** Christoph F. Kurat, Joseph T.P. Yeeles, Harshil Patel, Anne Early, John F.X. Diffley

**Affiliations:** 1 Clare Hall Laboratory, Francis Crick Institute, South Mimms, Hertfordshire EN6 3LD, UK; 2 Lincoln’s Inn Fields Laboratory, Francis Crick Institute, London NW1 1AT, UK

**Keywords:** DNA replication, chromatin, biochemistry

## Abstract

The integrity of eukaryotic genomes requires rapid and regulated chromatin replication. How this is accomplished is still poorly understood. Using purified yeast replication proteins and fully chromatinized templates, we have reconstituted this process in vitro. We show that chromatin enforces DNA replication origin specificity by preventing non-specific MCM helicase loading. Helicase activation occurs efficiently in the context of chromatin, but subsequent replisome progression requires the histone chaperone FACT (facilitates chromatin transcription). The FACT-associated Nhp6 protein, the nucleosome remodelers INO80 or ISW1A, and the lysine acetyltransferases Gcn5 and Esa1 each contribute separately to maximum DNA synthesis rates. Chromatin promotes the regular priming of lagging-strand DNA synthesis by facilitating DNA polymerase α function at replication forks. Finally, nucleosomes disrupted during replication are efficiently re-assembled into regular arrays on nascent DNA. Our work defines the minimum requirements for chromatin replication in vitro and shows how multiple chromatin factors might modulate replication fork rates in vivo.

## Highlights

• Reconstitution of eukaryotic chromatin replication with purified proteins
• Chromatin enforces origin-specific MCM loading
• FACT is essential for chromatin replication
• Nucleosomes are efficiently repositioned behind the replication fork

## Graphical Abstract

**Figure:**
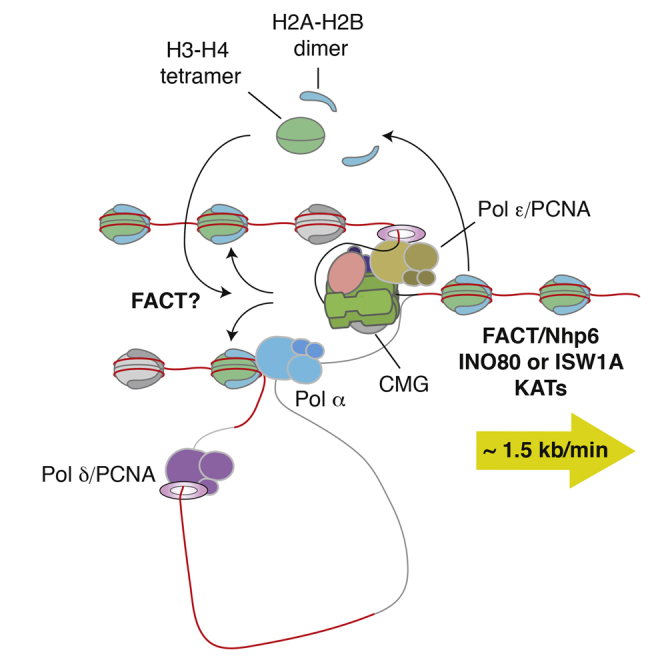

## Teaser

By reconstituting chromatin replication with purified proteins, Kurat et al. show that timely progression of the replisome through chromatin requires a complex interplay between FACT, Nhp6, chromatin remodelers, and lysine acetyltransferases. Parental nucleosomes are efficiently re-assembled in the back of the replisome and positively influence lagging-strand synthesis.

## Introduction

Eukaryotic genomes are packaged into nucleosomes comprising 147 bp of duplex DNA wrapped around a histone octamer containing two copies each of the four core histones (H2A, H2B, H3, and H4) (Luger et al., 1997). Histones are highly basic proteins and nucleosomes are therefore very stable structures, requiring, for example, high salt concentrations for their removal from chromatin. Within this context, the replication machinery must define sites of replication initiation (origins), load the MCM replicative helicase, and activate it by converting it to the CMG (Cdc45-MCM-GINS) complex. Each and every nucleosome then must be transiently disrupted to allow duplex unwinding and DNA synthesis by the replisome. After passage of the replication forks, nucleosomes composed of histones from parental nucleosomes as well as newly synthesized histones must be rapidly re-assembled on both leading and lagging-strand replication products. Many “chromatin factors” have been described that affect chromatin structure or dynamics including histone chaperones, nucleosome remodelers, and enzymes that covalently modify histone subunits (Campos and Reinberg, 2009, Swygert and Peterson, 2014). The roles of these proteins in transcription, DNA repair, and DNA damage signaling have been well studied. Nonetheless, roles for these factors in chromatin replication are still poorly defined.

It may be that the eukaryotic replisome can replicate chromatin without additional factors, since a heterologous replisome from the bacteriophage T4 can replicate through nucleosomal DNA on its own (Bonne-Andrea et al., 1990). However, there is considerable evidence that additional chromatin factors play at least some part in this process (Alabert and Groth, 2012). Two histone chaperones have been implicated in eukaryotic DNA replication fork progression in vivo. FACT (facilitates chromatin transcription) is a strong candidate based on its physical association with both the CMG helicase and the lagging-strand DNA polymerase α (Pol α) (Gambus et al., 2006, Orphanides et al., 1998, Orphanides et al., 1999, Reinberg and Sims, 2006, Wittmeyer and Formosa, 1997). Consistent with this possibility, genes encoding both FACT subunits (Pob3 and Spt16) are essential for viability in yeast and a *pob3* hypomorphic mutant exhibits hydroxyurea sensitivity (Schlesinger and Formosa, 2000). FACT is essential for replication in *Xenopus* egg extracts (Okuhara et al., 1999) and deletion of the Pob3 ortholog in chicken DT40 cells causes a reduction in replication fork rates but not origin firing (Abe et al., 2011). Another histone chaperone, Asf1, interacts with MCM via histones H3-H4 in human cells, and depletion of Asf1 inhibits replisome progression during the S phase (Groth et al., 2007). Overexpression of histones H3-H4 has similar effects to Asf1 depletion, suggesting that Asf1 plays an important role in coordinating unwinding with histone dynamics at the fork. In contrast to genes encoding FACT, the ASF1 gene is not essential in yeast. In addition to FACT and Asf1, the N terminus of the Mcm2 subunit of the replicative CMG helicase has been shown to act as a histone H3-H4 chaperone (Foltman et al., 2013, Huang et al., 2015, Ishimi et al., 1998, Richet et al., 2015, Saade et al., 2009). Similar to Asf1, mutation of this domain has relatively mild phenotypes in budding yeast (Foltman et al., 2013).

Roles for other chromatin factors in replication are less clear (Alabert and Groth, 2012). It may be that nucleosome remodelers and histone modifiers as well as the non-essential histone chaperones play little or no role in normal chromatin replication. Alternatively, they may be required for essential replication processes but may be highly redundant. These are difficult questions to address in vivo in part because of this potential redundancy and in part because chromatin factors including FACT also play key roles in gene expression, thus potentially affecting replication indirectly. Biochemical systems to address this in vitro have been lacking, so we set out to reconstitute this process with purified proteins.

## Results

### Chromatin Enforces Origin Specificity

We assembled nucleosomes on plasmid DNA with recombinant yeast histones, the histone chaperone Nap1, and the nucleosome remodeler ISW1A (Figure S1A) as previously described (Vary et al., 2004). Because histones were expressed in *Escherichia coli* (Kingston et al., 2011), they should not harbor any covalent marks. Analysis of the nucleosome arrays produced by micrococcal nuclease (MNase) digestion showed a high density of evenly spaced nucleosomes in the population (Figure S1B). A similarly dense array was obtained with either linear or circular DNA attached to magnetic beads as well as circular plasmid DNA in solution (Figures S1B–S1D).

**Figure 1:**
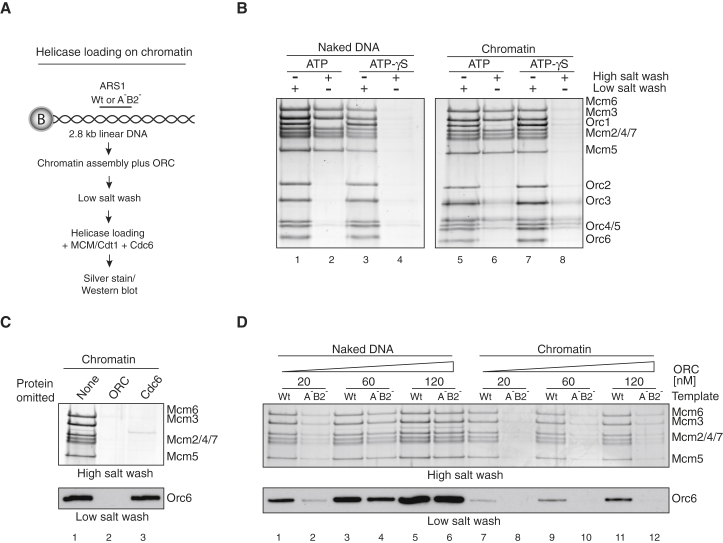
Loading of the MCM Complex on Chromatin (A) Reaction scheme for chromatin assembly and MCM loading on ARS1-containing 2.8-kb linear DNA coupled to paramagnetic beads. (B) Silver-stained gels of MCM loading reactions on naked DNA (left) compared to chromatin (right). In this and all subsequent experiments on bead-coupled DNA, ORC was added to the chromatin assembly reaction, followed by two 0.3-M K-acetate washes prior to MCM loading. Reactions were performed in the presence of 2 mM ATP or ATPγS. Loading reactions were washed either with 0.3 M K-acetate (low salt wash) or with 0.5 M NaCl (high salt wash). (C) MCM loading in the presence and absence of ORC and Cdc6. Loading reactions were conducted as shown in (A). ORC binding was assessed after two low salt washes by immunoblotting using an antibody recognizing the Orc6 subunit. (D) MCM loading and ORC binding on naked DNA and chromatin using wild-type (WT) and mutant ARS1 (A^−^B2^−^) origin DNA sequences. Reactions were performed as in (A) with indicated amounts of ORC. See also Figures S1 and S2.

To characterize this further, we assembled chromatin on a 2.8-kb fragment of yeast DNA from the *TRP1-GAL3* locus with the ARS1 replication origin at its center, which was attached at one end to magnetic beads via a biotin-streptavidin linkage (Figure 1A; Figures S1A and S1B). We then digested the chromatinized templates to completion with MNase and deep-sequenced mononucleosomal DNA. Figure S2A presents normalized read numbers across the entire sequence, showing that phased nucleosomes can be found across the region. Despite the apparent clear phasing of nucleosomes in the bulk population (Figure S1), however, there are regions, for example, in the 3′ half of the *TRP1* gene, where phasing was less precise, as previously documented (Thoma et al., 1984). Consistent with previous work, there was a gap in the nucleosome map corresponding to the origin sequence, indicating that the origin was nucleosome free (Eaton et al., 2010). This gap was visible even in the absence of the origin recognition complex (ORC), but the presence of ORC during chromatin assembly further suppressed encroachment of nucleosomes into the origin (Figures S2A and S2B). The third panel of Figure S2B shows that this suppression of nucleosome encroachment occurred even when ORC was added after chromatin assembly. In all subsequent experiments, ORC was present during chromatin assembly.

The first step in DNA replication is the loading of the MCM helicase as a double hexamer bound around double-stranded DNA by ORC, Cdc6, and Cdt1 (Evrin et al., 2009, Remus et al., 2009). Figure 1B shows that, on both naked DNA and chromatin, MCM, along with the loading factors, was bound to DNA in ATP and ATPγS after a low salt wash. MCMs were assembled into a high-salt-resistant complex, which is a hallmark of loaded MCM double hexamers, in ATP but not ATPγS. ORC was reproducibly slightly more resistant to removal from chromatin with high salt, suggesting that it may interact with nucleosomes, consistent with previous work (Hizume et al., 2013). As is the case for MCM loading on naked DNA, this high-salt-resistant MCM complex on chromatin was dependent on both ORC and Cdc6 (Figure 1C). A high salt wash after chromatin assembly removed ISWA and Nap1 (Figures S2C and S2D). MCM loading, however, was not affected (Figure S2E), indicating that ISWA and Nap1 are not required for MCM loading on chromatin.

Previous work has shown that MCM loading on naked DNA does not exhibit strong origin dependence (Remus et al., 2009). Consistent with this, Figure 1D shows that MCM loading on naked DNA containing a wild-type origin or a mutant origin that lacks high-affinity ORC binding sites (A^−^B2^−^) (Bell and Stillman, 1992, Marahrens and Stillman, 1992, Remus et al., 2009) occurred equally efficiently at high ORC concentrations (lanes 5 and 6); dependence on the functional origin was only seen at relatively low ORC concentrations (e.g., lanes 1 and 2). On chromatin, MCM loading was efficient on the wild-type origin, but loading was greatly reduced on the mutant origin over a wide range of ORC concentrations. The lower panel of Figure 1D shows that these effects are reflected in ORC binding after a low salt wash: there was less ORC bound to chromatin, and specificity for the wild-type origin was maintained at all ORC concentrations tested. From these experiments, we conclude that chromatin enforces origin specificity by suppressing non-specific ORC binding.

### Chromatin Inhibits Replisome Progression

**Figure 2:**
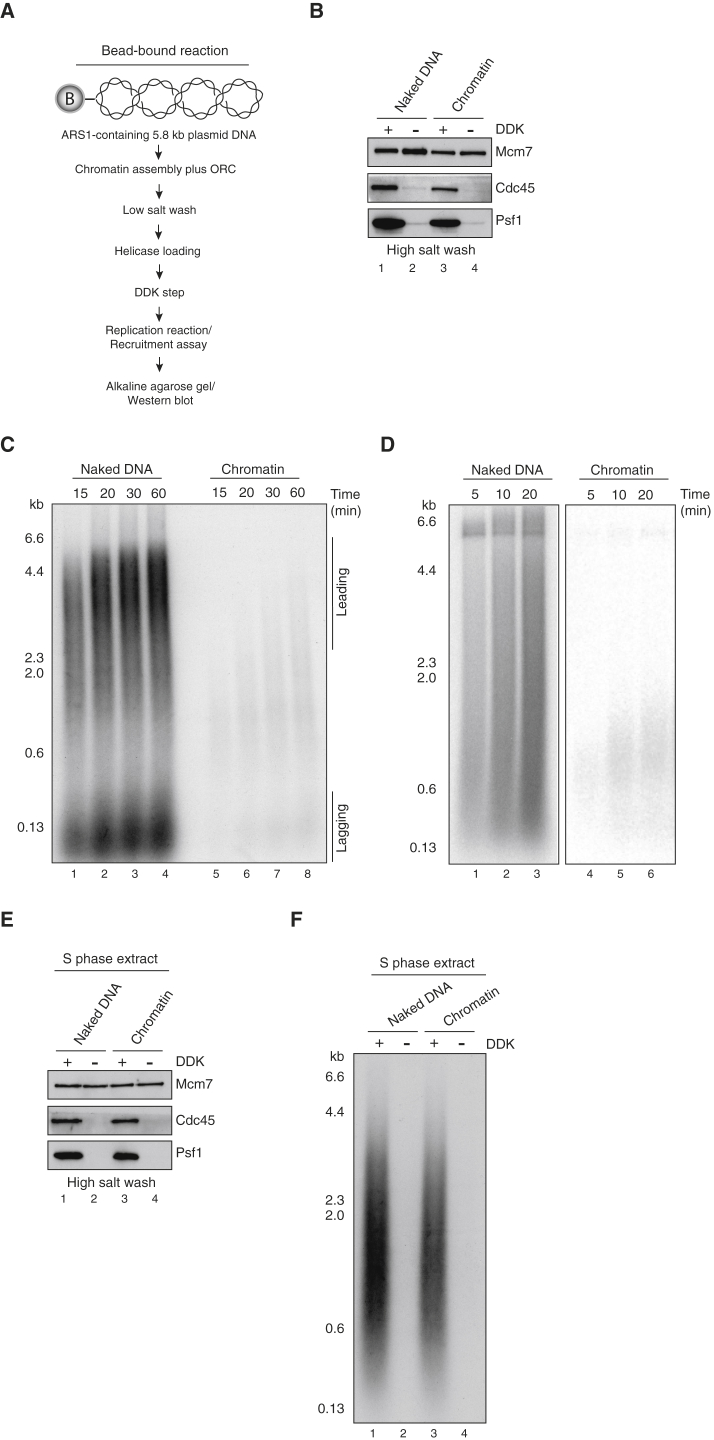
Chromatin Inhibits DNA Replication In Vitro (A) Reaction scheme for replication reactions and CMG recruitment on 5.8-kb circular bead-bound ARS1-containing templates using S phase extract or purified proteins. Chromatin assembly and MCM loading were performed as in Figure 1A. Loaded MCMs were further phosphorylated with DDK before they were added to an S phase extract or purified replication proteins. (B) CMG recruitment on naked DNA and on chromatin in the presence of purified initiation and replication factors (Sld3/7, Cdc45, Dpb11, Polε, GINS, Sld2, Mcm10, and S-CDK). Reactions were performed as in Figure 3A. Beads were collected and washed with 0.3 M KCl. Recruitment of the CMG with or without DDK was assessed by immunoblotting using antibodies recognizing Cdc45, Psf1 (GINS), and Mcm7 (MCM) subunits. (C) Replication reactions on naked DNA and on chromatin conducted as shown in (A) using the minimal replication system (Yeeles et al., 2015). In this and all subsequent replication reactions, DNA was visualized by incorporation of [α^32^P] deoxycytidine triphosphate (dCTP) into nascent DNA and products were separated through a 0.7% alkaline agarose gel. (D) Replication reactions on naked DNA and on chromatin using the complete replication system as described in Yeeles et al., 2016. (E) CMG recruitment in S phase extract on either naked DNA or chromatin in the presence and absence of DDK phosphorylation. (F) Replication reactions on naked DNA compared to chromatin using S phase extract in the presence or absence of DDK.

We next asked whether the loaded MCM complex could be efficiently converted to the CMG helicase in the context of chromatin. To do this, we assembled chromatin and loaded MCM as in the previous section, phosphorylated it with Dbf4-dependent kinase (DDK), and added the remainder of the required firing factors (Figure 2A) (Yeeles et al., 2015). The CMG is stable to high salt extraction, and Figure 2B shows that DDK-dependent CMG was formed on chromatin almost as efficiently as it was on naked DNA. However, DNA synthesis was strongly inhibited in the context of chromatin with either the minimal replisome (Yeeles et al., 2015; Figure 2C) or with the reconstituted replisome (without FACT) described in our accompanying manuscript in this issue of *Molecular Cell* (Yeeles et al., 2016) (Figure 2D). In all subsequent experiments with purified proteins, this reconstituted replisome was used. Thus, chromatin does not inhibit CMG assembly but it effectively prevents replisome progression, indicating that additional factors are required for chromatin replication.

To identify factors that might contribute to replisome progression through chromatin, we next asked whether an S phase extract (Gros et al., 2014, Heller et al., 2011, On et al., 2014) could replicate our chromatin template. As shown in Figure 2E, CMG assembly was supported on the chromatin template in extract, and this template replicated in a DDK-dependent manner almost as efficiently as naked DNA in these extracts (Figure 2F). Therefore, some factors in the extract must be acting with the replisome. To identify candidates, we performed quantitative, label-free mass spectrometry on the chromatin templates in the extracts. As described previously (On et al., 2014), we used intensity-based absolute quantification (IBAQ) (Schwanhäusser et al., 2011) to measure the relative abundance of proteins on assembled chromatin either with or without prior DDK treatment, and we plotted the log_10_ (IBAQ) scores (Figure S3). As expected, histones and MCM subunits were highly abundant and were found along the diagonal line, indicating that they were present at roughly equal levels with or without DDK. Focusing on chromatin factors, we found two lysine acetyltransferases enriched in the presence of DDK: Esa1, part of the NuA4 complex, and Gcn5, part of the SAGA complex. Although the enrichment of Gcn5 was modest, the enrichment of Esa1 was considerable (almost two orders of magnitude higher in the +DDK sample). The histone chaperones Asf1, FACT, and Nap1 were also identified. Finally, the remodelers INO80, RSC, and ISW1A were identified. Samples were not washed with high salt before they were added to the extract, so we cannot rule out that some ISW1A and/or Nap1 may have been carried over from the chromatin assembly step. None of these factors showed a significant enrichment in the +DDK sample, although most proteins were slightly above the diagonal. Nonetheless, these were the most abundant chromatin factors on our templates, so we investigated their roles in replication. The full list of proteins identified is provided in Table S3.

### FACT Is Required for Chromatin Replication

**Figure 3:**
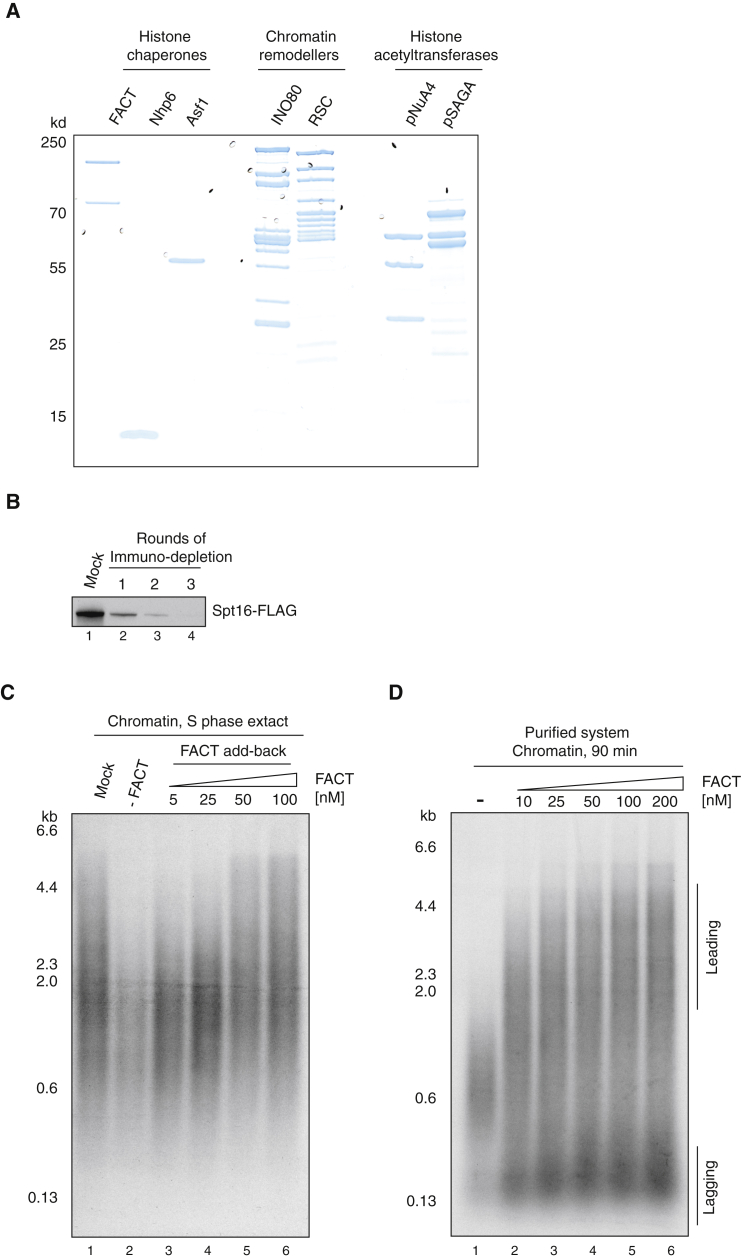
FACT Is Necessary and Sufficient for Chromatin Replication (A) Purified histone chaperones, Nhp6 protein, chromatin remodelers, and histone acetyltransferases analyzed by SDS-PAGE with Coomassie staining. (B) Immunodepletion of the Spt16 subunit of the FACT complex from an S phase extract (yCFK2) assessed by immunoblotting using an antibody recognizing the FLAG epitope. (C) Dependence of chromatin replication on in S phase extract. Spt16 was immunodepleted as in (B) and replication reactions on chromatin were performed as described in (A). Indicated amounts of purified FACT were added back to the immunodepleted extract. (D) Effect of FACT on chromatin replication using the complete replication system. See also Figures S3–S5 and Table S3.

To assess the roles of these proteins in replisome progression, we expressed and purified them all (Figure 3A; Figure S4). In addition to FACT and Asf1, we also expressed and purified Nhp6, a small HMG box-containing protein that is known to work with FACT (Brewster et al., 2001, Formosa et al., 2001, Stillman, 2010) and was identified by mass spectrometry (Figure S3). The complete INO80 complex was purified after overexpression in yeast; the endogenous RSC complex was also purified from yeast. The NuA4 and SAGA complexes each have large numbers of subunits, but sub-assemblies have been identified that contain full acetyltransferase activity (pNuA4 and pSAGA) (Barrios et al., 2007). These were expressed and purified from *E. coli*.

In the accompanying manuscript (Yeeles et al., 2016), we showed that FACT has no effect on replication of naked DNA. To begin to examine the role of FACT in chromatin replication, we depleted it from our S phase extracts (Figure 3B). As shown in Figure 3C, FACT-depleted extracts were defective in replicating a chromatinized template, and addition of purified FACT restored replication activity of these extracts. Moreover, addition of FACT to the purified replication system increased the lengths of leading-strand replication products and the overall amount of lagging-strand products with purified proteins (Figure 3D). To rule out any contribution to replication from the ISW1A remodeler and the Nap1 chaperone used to assemble chromatin, we washed chromatin with high salt before replication, which removed ISW1A and Nap1 (Figures S2C and S2D). Figure S5 shows that replication of this high-salt-washed chromatin was stimulated by FACT to a similar extent as chromatin, which was not washed with high salt. From these experiments, we conclude that FACT is necessary and, to some extent, sufficient for chromatin replication.

### Nhp6, INO80, ISW1A, and Histone Acetylation Stimulate Replication with FACT

**Figure 4:**
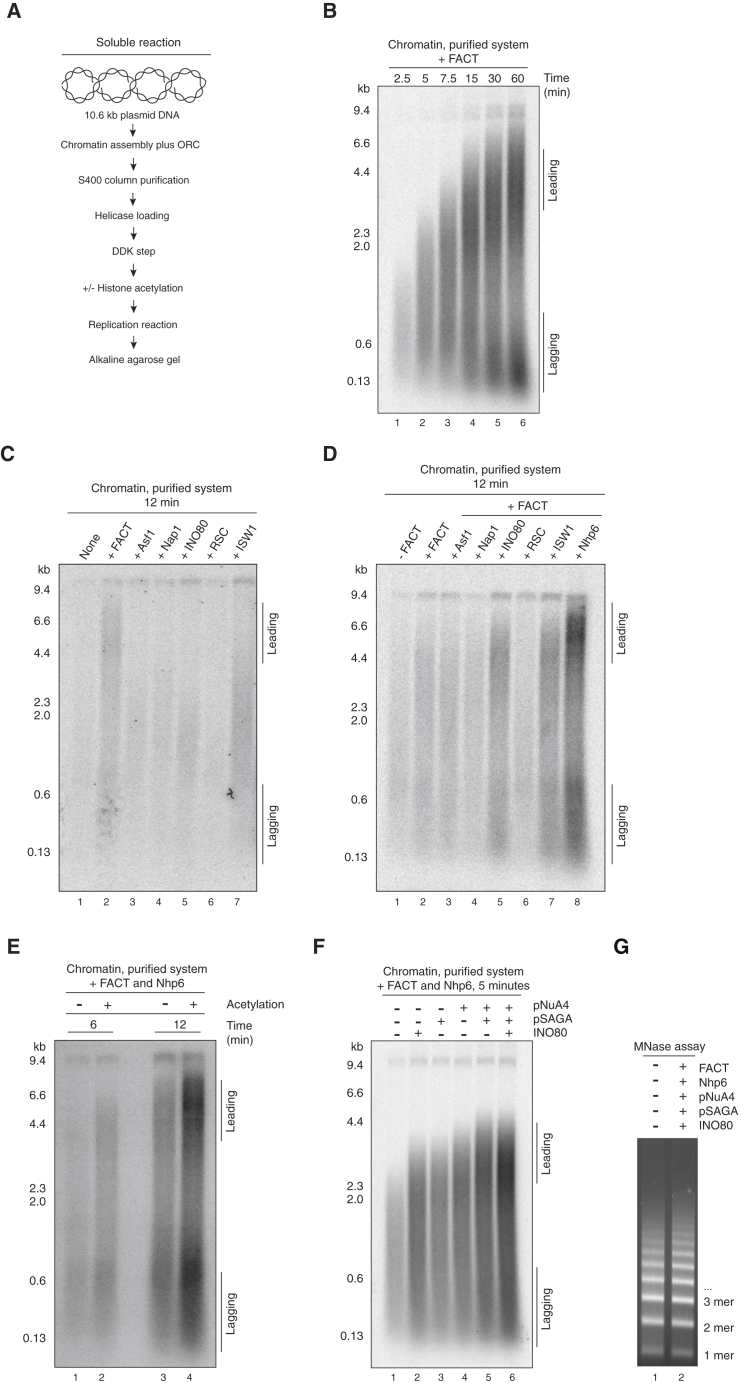
Contribution of Chromatin Remodelers and Lysine Acetyltransferases to Chromatin Replication (A) Reaction scheme for soluble replication reaction on chromatin. Chromatin was assembled on ARS1-containing 10.6-kb plasmid DNA in solution in the presence of ORC. ORC-containing chromatinized circles were then isolated for subsequent steps by gel filtration. All subsequent replication reactions were performed using the complete replication system. (B) Time course of a soluble chromatin replication reaction using the scheme shown in (A) in the presence of FACT added at the beginning of the reaction. (C) Effect of histone chaperones and chromatin remodelers on chromatin replication assessed individually. (D) Effect on chromatin replication of adding histone chaperones, chromatin remodelers, or Nhp6 together with FACT. (E) Histone acetylation by catalytic subcomplexes of NuA4 (pNuA4) and SAGA (pSAGA) stimulate chromatin replication. Reactions were performed as described in (A). After DDK treatment, nucleosomes were acetylated using purified pNuA4, pSAGA, and acetyl-coenzyme A. (F) Effect of adding of pNuA4, pSAGA, and INO80 together with FACT and Nhp6 on chromatin replication. (G) Bulk chromatin with or without FACT, Nhp6, INO80, and histone acetylation was assessed by MNase digestion following native agarose gel electrophoresis and ethidium bromide staining. See also Figures S6 and S7.

We noticed that replication was slow, even with FACT. We considered that this may be due to interference caused by interactions between chromatin and the magnetic beads. We developed a protocol to assemble and purify chromatin on soluble 10.6-kb plasmids before replicating this in the soluble replication system described in the accompanying manuscript (Yeeles et al., 2016) (Figure 4A). Replication in this soluble system produced near full-length leading-strand replication products; however, even in this soluble system, replication of chromatin with FACT alone was still relatively slow, not reaching completion until 30–60 min (Figure 4B).

We next looked at whether the other chromatin factors (Figure 3A) could stimulate replication on chromatin by looking at their effect on replication products at an early time point (12 min). Figure 4C shows that, while FACT could clearly stimulate replication at this time point, Asf1, Nap1, INO80, and RSC had no effect on replication. ISW1A had a very small effect on overall incorporation but did not significantly increase the length of the products, suggesting that it may weakly promote some step in initiation. We next asked whether any of these factors could stimulate replication with FACT, again at 12 min. Figure 4D shows that Asf1, Nap1, and RSC did not stimulate replication; in fact, RSC slightly but reproducibly inhibited synthesis. By contrast, the INO80 and ISW1A remodelers increased both the overall incorporation in the presence of FACT as well as the length of the leading-strand products, resulting in a clear distinction between leading and lagging-strand products even at this early time point. The biggest effect, however, was seen with Nhp6, which stimulated both overall incorporation and leading-strand length at this early time point more than either INO80 or ISW1A. Nhp6 had little or no effect on replication in the absence of FACT, nor did it stimulate replication with any of the other purified factors (Figure S6A), indicating that its effects are specific for FACT. In the presence of FACT and Nhp6, both ISW1A and INO80 further stimulated replication (Figure S6B).

We next investigated the effects of histone acetylation on chromatin replication. Figure S7A shows that the pNuA4 and pSAGA acetyltransferases are active and exhibit specific reactivity toward histone H4 and H3, respectively. Figure S7B shows that, in combination, they appeared to stimulate the rate of replication with FACT alone, and the combination of these acetyltransferases with FACT and Nhp6 greatly stimulated replication at early time points (Figure 4E) in an acetyl-coenzyme A-dependent manner (Figure S7C).

To assess the effects of combinations of factors, we examined replication at an even earlier time point (5 min). Figure 4F shows that, individually, INO80, pSAGA, and pNuA4 all stimulate replication with Nhp6 and FACT. Combining pSAGA and pNuA4 stimulates beyond the level of either individual acetyltransferase. The combination of INO80 with the two acetyltransferases stimulated incorporation at this early time point even further. Importantly, the full combination of FACT, Nhp6, INO80, pSAGA, and pNuA4 had no effect on the MNase digestion pattern (Figure 4G), indicating that the stimulation of replication was not due to non-specific removal of nucleosomes from the template. Multiple RSC subunits contain bromodomains, which bind acetylated histones, so we asked whether histone acetylation affected the ability of RSC to promote replication. We found that acetylation greatly stimulated the recruitment of RSC to chromatin (Figure S7D), but this recruited RSC did not stimulate replication. Instead, RSC more strongly inhibited replication after histone acetylation (Figure S7E).

Figure S7F shows that FACT was essential for chromatin replication even in the presence of the other chromatin factors, consistent with it being a key player in chromatin replication. The full complement of chromatin factors also greatly stimulated replication of a linear chromatin template (Figure S7G), indicating that the inhibition of replication is not simply due to some topological problems caused by chromatin. The presence of two discrete leading-strand products of 1.6 and 1.2 kb in this experiment also shows that most initiation occurs in or near the origin.

**Figure 5:**
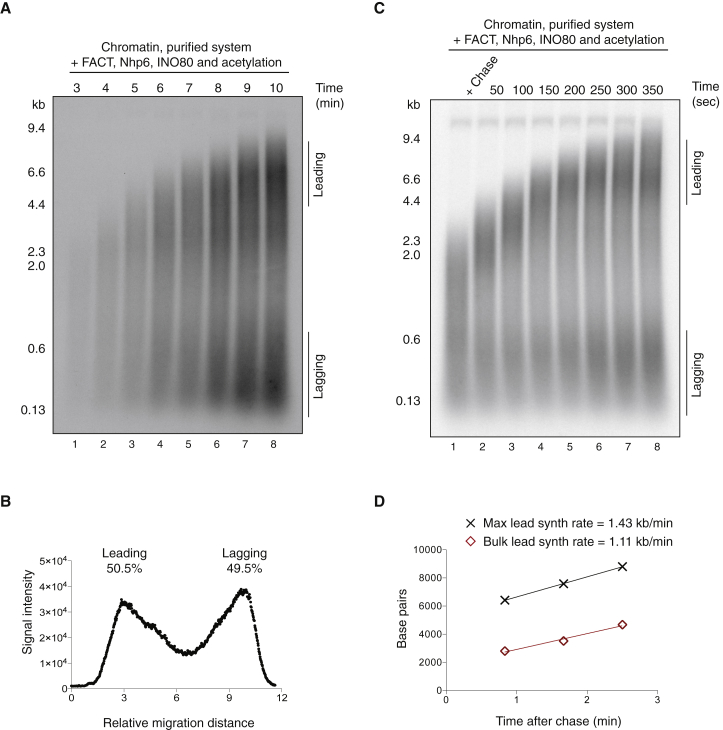
FACT, Nhp6, INO80, and Histone Acetylation Together Result in In Vivo Rates of Replisome Progression through Chromatin (A) Time course of chromatin replication reactions as conducted in Figure 4A with FACT, Nhp6, INO80, and histone acetylation. (B) Lane profile for the 10-min time point in Figure 6A. (C) Pulse chase experiment to measure replication rates with the same reaction setup as in Figure 6A. For the pulse, the dCTP concentration was reduced to 4 mM. Following a 3-min incubation, unlabeled dCTP was added to a final concentration of 150 mM, and time points were taken every 50 s. (D) Maximum (front) and peak product length for the experiment shown in (C). To derive maximum and bulk leading-strand synthesis rates, data were fitted to a linear regression.

A time course with the full combination of positive-acting chromatin factors showed the appearance of full-length products within 10 min (Figure 5A), suggesting rapid replisome progression. As with the complete replisome on naked DNA (Yeeles et al., 2016), this replication resulted in equal amounts of leading and lagging-strand synthesis (Figure 5B). To quantify the fork rate, we performed a pulse chase experiment (Figure 5C). Analysis of this experiment (Figure 5D) indicates that leading-strand replication in this system proceeds at a maximum rate of approximately 1.4 kb/min, with bulk synthesis being 1.1 kb/min.

### Chromatin Promotes Lagging-Strand Replication

**Figure 6:**
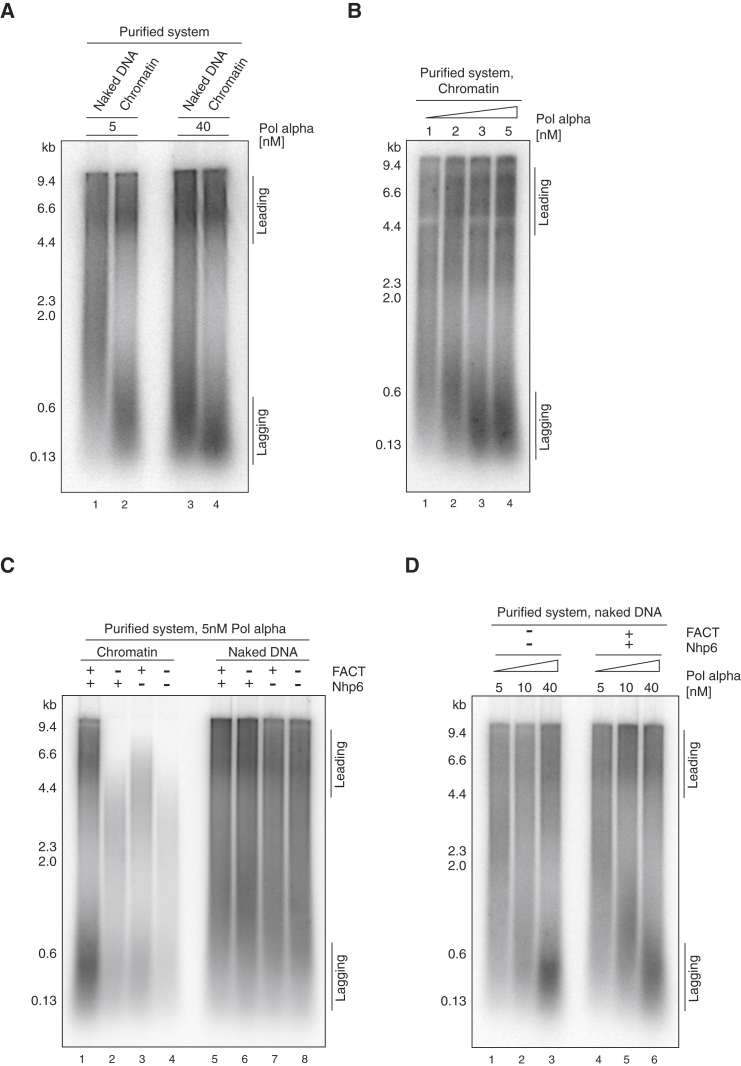
Chromatin Influences Lagging-Strand Size (A) Replication reactions on naked DNA and chromatin were conducted as in Figure 4A with the amounts of Pol α as indicated. For chromatin replication, nucleosomes were acetylated and FACT, Nhp6, and INO80 were added. (B) Replication reactions on chromatin as in (A) with indicated amounts of Pol α. (C) Replication reactions on naked DNA versus chromatin in the presence or absence of FACT and Nhp6. Nucleosomes were not acetylated and INO80 was omitted. (D) Replication reactions on naked DNA as in (A) in the presence or absence of FACT and Nhp6. Pol α was added at the indicated amounts. See also Figure S7.

In the accompanying manuscript (Yeeles et al., 2016), we show that, on a naked DNA template, the size of lagging-strand products is highly dependent on the concentration of Pol α across a wide range, indicating that Pol α acts distributively in this system. We noticed that lagging-strand products from chromatin templates, even with the full combination of factors, were considerably smaller and more discrete than those we routinely see on naked DNA. As shown in Figure 6A (lanes 1 and 3), lagging-strand products on naked DNA were considerably longer (∼2 kb) at 5 nM Pol α than at 40 nM (∼0.6 kb). By contrast, lagging-strand products from the chromatin template (lanes 2 and 4) were relatively short (0.15–0.5 kb) at both 5 and 40 nM Pol α. Further reduction of Pol α had little effect on lagging-strand length until 1 nM, at which point even leading-strand synthesis was reduced (Figure 6B). These experiments show that lagging-strand product length was relatively constant over a wide range of Pol α concentrations, indicating that Pol α no longer acts distributively in the context of chromatin. Because FACT interacts with both CMG and Pol α (Foltman et al., 2013, Wittmeyer and Formosa, 1997), we tested whether FACT alone could account for this effect. As shown in Figure 6C, the presence of FACT and/or Nhp6 did not lead to shorter leading-strand products on naked DNA at a low Pol α concentration, and Pol α still appeared to act distributively in lagging-strand synthesis across a range of Pol α concentrations (Figure 6D). Moreover, despite differential effects on overall synthesis, short lagging-strand products were seen when chromatin was used as a template in the absence of FACT and/or Nhp6 and any remodelers or acetyltransferases (Figure 6C, lanes 1–4). The presence of Ctf4 in the reaction when chromatin was used as the template made no difference in lagging-strand size at various Pol α concentrations (Figure S7H). Taken together, these experiments indicate that it is the presence of chromatin, rather than any of the chromatin factors, that functionally tethers Pol α to the replication fork.

### Nucleosomes Are Re-deposited on Nascent DNA

**Figure 7:**
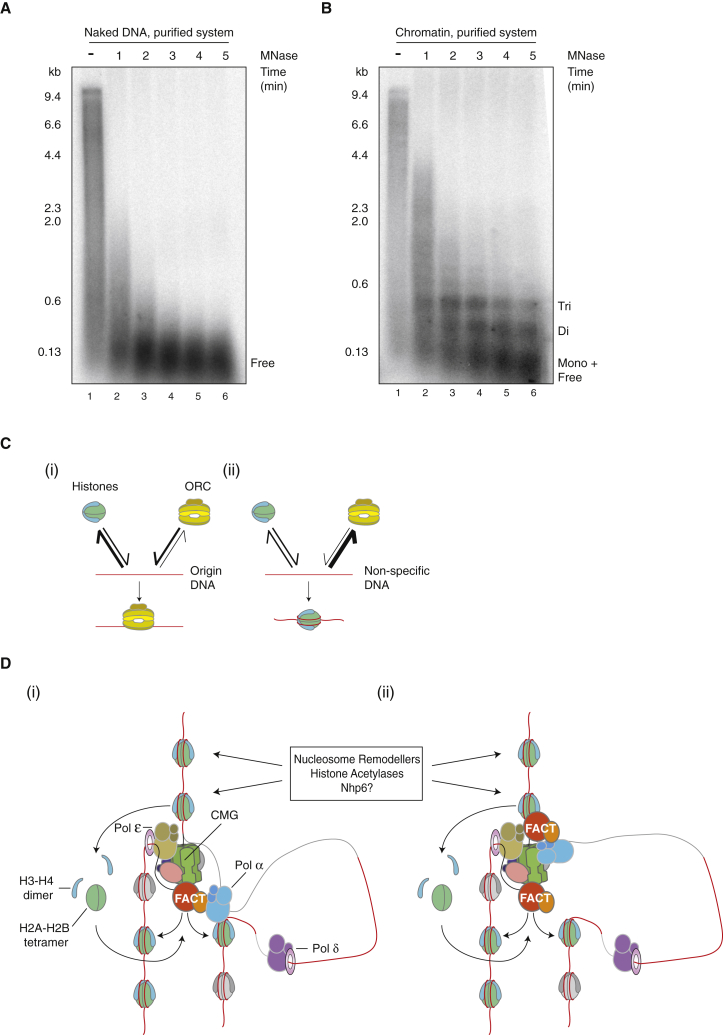
Nucleosomes Are Re-deposited on Nascent DNA (A and B) Nucleosomes are re-deposited on nascent DNA. MNase digestion of replicated products of naked DNA compared to those of chromatin. Replication products were treated with 100 U MNase and samples were taken every minute, quenched with EGTA, and analyzed on a 1.3% alkaline agarose gel. Replication products were visualized by autoradiography. (C) Model of how chromatin influences origin selection. See the Discussion for details. (D) Model of FACT-dependent replisome progression through chromatin. Parental nucleosomes are in green/light blue. Nucleosomes including newly synthesized histones are in gray/light gray. Double-stranded DNA is in red and single-stranded DNA is in gray. See the Discussion for details.

Finally, we tested whether the parental nucleosomes assembled on our chromatin templates were transferred to nascent DNA by digesting the replicated products with MNase. Figures 7A and 7B show that replication products from naked DNA were rapidly digested to a small size by MNase, while MNase digestion of replication products from chromatin revealed a characteristic ladder of repeating nucleosomes. Free histones were removed from our chromatin templates by gel filtration before they were added to the reactions, and the chromatin factors did not cause detectable displacement of histones from DNA (Figure 4G). Consequently, it is highly likely that nucleosomes assembled on nascent DNA derive from histones displaced from parental DNA during replication. To assess the efficiency of this nucleosome assembly, we quantified the amount of labeled product in the di- and tri-nucleosome bands in lane 4 of Figure 7B, relative to the total labeled product in lane 1, which showed that these nucleosomal bands accounted for approximately 34% of the total replication product. The mononucleosome band partially co-migrated with the digested free DNA, so this was not included in our analysis. If all of the parental nucleosomes displaced by the replisome were redeposited onto the nascent DNA, we would predict that the nucleosomal DNA would account for a maximum of 50% of the labeled product, because the DNA duplicates during replication. Thus, at least 68% (34 of 50) of the parental nucleosomes appear to be redeposited onto nascent DNA. This is likely to be an underestimate since it does not include the mononucleosome band.

## Discussion

We have described the reconstitution of DNA replication on chromatin templates. Fork rates measured in this system fall well within the range of fork rates measured in vivo. Results described here have allowed us to identify three crucial roles for chromatin in replication: origin selection, fork rate modulation, and priming of lagging-strand synthesis.

### Origin Selection

Previous work showed that replication origins have an inherent tendency to exclude nucleosomes even in the absence of ORC (Eaton et al., 2010). ORC reinforces this nucleosome free region (NFR), probably because its tight binding to specific DNA sequences acts as a barrier to new nucleosome assembly or sliding of adjacent nucleosomes into the NFR (Figure 7C, i). We have extended these findings by showing that chromatin also enforces origin specificity by suppressing non-specific ORC binding. ORC binding to non-specific DNA has a high off rate and we propose that as a consequence (Figure 7C, ii), nucleosomes act as a barrier to ORC binding. Metazoan ORC is a non-specific DNA binding protein (Vashee et al., 2003). We suggest that the suppression of ORC binding by chromatin described here may help restrict ORC to NFRs, a feature seen in mammalian replication origins in vivo (Cayrou et al., 2011, Lubelsky et al., 2011, MacAlpine et al., 2010).

### FACT and Replisome Progression through Chromatin

The most important chromatin factor required for replisome progression through nucleosomes is the histone chaperone FACT: although FACT does not affect replication of naked DNA (Yeeles et al., 2016; Figure 6C), loss of FACT severely inhibited chromatin replication in extracts (Figure 3C) and in the fully reconstituted system (Figure S7F), and addition of FACT to the purified replication system was sufficient for complete but slow replication (Figure 3D). None of the other chaperones, remodelers, or lysine acetyltransferases tested could substitute for FACT in chromatin replication (Figure 4C; Figure S7F).

Because FACT has no effect on bulk nucleosome occupancy or spacing, even with other chromatin factors (Figure 4G), and because FACT interacts with multiple replisome components and functions in chromatin replication at concentrations similar to those of replication factors rather than histones, we favor the idea that FACT acts with the replisome to promote replication through chromatin. FACT may act by displacing nucleosomes ahead of the fork, and/or by re-depositing nucleosomes behind the fork. In transcription, FACT destabilizes nucleosomes ahead of RNA polymerase (Hsieh et al., 2013, Orphanides et al., 1998) but also prevents release of histones and promotes rapid re-establishment of chromatin after transcription (Hainer et al., 2012, Jamai et al., 2009). Recent evidence in yeast showing genetic and physical interactions between FACT and replication-coupled nucleosome assembly (Yang et al., 2016) suggests that FACT may also promote re-establishment of chromatin after replication. This would be consistent with the very efficient re-deposition of histones from parental nucleosomes onto nascent DNA (Figures 7A and 7B). We suggest two models (Figure 7D) for how nucleosomes ahead of the replication fork are displaced. First, disruption of nucleosomes ahead of the fork may not require FACT but may simply be a consequence of DNA unwinding by CMG, similar to the way the T4 replisome can displace nucleosomes (Figure 7D, i) (Bonne-Andrea et al., 1990). In this model, FACT’s crucial role is in “accepting” these displaced histones, transferring them behind the fork into nucleosomes on the leading and lagging strands. In the absence of FACT, these displaced histones somehow interfere with the replisome, preventing normal progression. A second model (Figure 7D, ii) envisions a role for FACT in disrupting nucleosomes ahead of the fork. In this model, interaction of FACT with the CMG helicase (Foltman et al., 2013), Pol α (Wittmeyer and Formosa, 1997), or some other replisome component positions FACT to act ahead of the replication fork, where it contributes to displacing parental nucleosomes.

### Lagging-Strand Synthesis

A functional interaction between FACT, Pol α, and nucleosomes is also attractive because of the effect that chromatin has on lagging-strand synthesis. As discussed in the accompanying manuscript (Yeeles et al., 2016), Pol α, despite its interaction with Ctf4 and Mcm10, is not functionally tethered to the replisome; but on chromatin, lagging-strand product lengths are short and relatively constant over a wide range of Pol α concentrations. It may be that interactions between Pol α, FACT, and nucleosomes, either behind (Figure 7D, i) or ahead of (Figure 7D, ii) the fork, physically tether Pol α to the fork. Alternatively, chromatin may not physically tether Pol α to the replication fork; rather, pausing of the replisome at each nucleosome ahead of the fork may promote a priming event behind the fork, perhaps via some structural change in the replisome that promotes transient recruitment of Pol α.

Chromatin assembly behind the replication fork is thought to dictate the positioning of lagging-strand product junctions by restricting strand displacement synthesis by Pol δ (Smith and Whitehouse, 2012). We do not have a flap endonuclease like Fen1 or Dna2 in our reactions, so the lengths of our products reflect the effect of chromatin on synthesis, not downstream cleavage. Our results, together with those of Smith and Whitehouse (2012), suggest that chromatin regulates the length of lagging-strand products by affecting both the rate of priming and the position of flap cleavage.

The regeneration of chromatin after DNA replication in vivo involves both re-deposition of parental histones into nucleosomes and assembly of newly synthesized histones into nucleosomes. Chromatin assembly factor 1 (CAF-1), the key histone chaperone in the de novo pathway (Smith and Stillman, 1989), is not required for the efficient re-deposition of parental histones onto nascent DNA as we have described here, suggesting that these are truly two distinct pathways for nucleosome assembly on nascent DNA. It will be important to determine how these processes are coordinated. The products after MNase digestion of nascent DNA were primarily mono-, di-, and tri-nucleosomes (Figure 7B), whereas the starting templates had more extensive nucleosome arrays (Figures S1B–S1D). Presumably, the de novo pathway will be required to regenerate the full nucleosome array.

### Modulation of Replication Fork Rate by Chromatin Factors

Our data show that Nhp6, along with nucleosome remodelers and lysine acetyltransferases, acts additively to modulate the replication fork rate. INO80 has a role in removing nucleosomes from around double-strand DNA breaks (Li and Tyler, 2016, Tsukuda et al., 2005) and is also involved in resolving replication-transcription conflicts (Poli et al., 2016). Loss of both INO80 and chromatin accessibility complex (CHRAC) remodelers in vivo causes reduced nucleosome accessibility to MNase around replication forks in methyl methanesulfonate (MMS) (Lee et al., 2015), consistent with the idea that these remodelers play some role in disrupting nucleosomes during replication through damaged DNA. Our results suggest that INO80 may also play a role in normal replication. The catalytic subunit of CHRAC is the Isw1 paralog Isw2. Moreover, CHRAC shares a subunit, Dpb4, with Pol ε (Iida and Araki, 2004), suggesting that CHRAC, like ISW1A, may also be able to accelerate the fork rate. It is likely that other nucleosome remodelers and histone modifiers are also able to contribute to replisome progression. We note that RSC is recruited more efficiently to acetylated nucleosomes, consistent with the presence of bromodomain-containing subunits (Figure S7E). We speculate that some remodelers may have effects in specific regions of the genome enriched in particular histone marks, as the ACF1-ISWI complex has a role in heterochromatin replication in human cells (Collins et al., 2002). We also note that, even with histone acetylation, RSC does not stimulate replisome progression (Figure S7D); indeed, acetylation promoted even further inhibition of replication by RSC, indicating that not all remodelers can promote replication. We do not currently know whether this inhibition of replication by RSC plays any role in regulating fork rate in vivo.

Whether any of the chromatin factors we have examined other than FACT are specifically targeted to replication forks is unknown. In contrast to FACT, Nhp6 is required at much higher levels than replication factors to exert its effect on replication, suggesting that it may act distributively on chromatin. That INO80 and CHRAC contribute to changes in nucleosome accessibility specifically around replication forks suggests that they may be targeted to replication forks (Lee et al., 2015). Recent work has shown that INO80 is recruited to replication forks in human cells through interaction with ubiquitylated H2A aided by BRCA1-associated protein-1 (BAP1) (Lee et al., 2014), but it is unclear whether or how INO80 is being recruited to forks in our system. We found that recruitment of Esa1 to chromatin was enhanced by DDK, suggesting some coupling of it with the replication fork (Figure S3). Both Gcn5 and Rtt109 can acetylate histone H3K9, a mark that was recently shown to precede passage of the replication fork (Bar-Ziv et al., 2016). It was shown that the H3K9Ac, which precedes the fork, requires Rtt109, while the H3K9Ac at promoters requires Gcn5, so it may be that Rtt109 is the more relevant acetyltransferase for bulk replisome progression. Nonetheless, Gcn5 can clearly contribute to replisome progression, at least in vitro, and may aid replisome progression in specific regions like promoters in vivo.

Based on our work, we propose that replication fork rates in vivo reflect a complex interplay between different chromatin factors and histone modifications. Replication fork speed in vivo can be significantly affected by expression of a number of oncogenes during oncogene-induced replicative stress. While it is often assumed that this reflects some misregulation of the DNA replication machinery, we suggest that it may in some cases reflect changes in the availability and distribution of nucleosome remodelers and various histone marks. Some oncogenes, like c-Myc, are known to dramatically affect gene expression patterns genomewide (Zeller et al., 2006), and this may indirectly affect fork rates through chromatin factors.

The reconstitution of efficient replication through chromatin with purified proteins represents a major advance in understanding how chromosomes are duplicated and will provide novel approaches to understand how marked nucleosomes and gene expression patterns are inherited during DNA replication.

## Experimental Procedures

### Yeast Strains and Proteins

Detailed information about yeast strain construction and protein purifications can be found in the Supplemental Experimental Procedures.

### Chromatin Assembly and MCM Loading

Chromatin was assembled on different DNA templates as described previously (Vary et al., 2004). MCM loading on chromatin was conducted as described in the Supplemental Experimental Procedures.

### Nucleosome Positioning

Chromatin was assembled on 2.8-kb linear DNA and mononucleosomal DNA was generated by MNase digestion. Mononucleosomal DNA was sequenced as described in the Supplemental Experimental Procedures.

### S Phase Extracts and Mass Spectrometry Analyses

S phase extracts were prepared as described previously (On et al., 2014) and in the Supplemental Experimental Procedures. Chromatin was assembled and MCMs were loaded as described. Samples were treated with 100 nM DDK for 30 min at 30°C. S phase extract was added and incubated for another 30 min. After two washing steps (45 mM HEPES-KOH [pH 7.6], 5 mM Mg(OAc)2, 0.02% NP-40, 10% glycerol, and 0.3 M KOAc) and MNase treatment to digest DNA, chromatin-associated proteins were analyzed by mass spectrometry as described previously (On et al., 2014).

### CMG Recruitment and Replication Reactions

CMG recruitment was performed on ARS1-containing bead-coupled plasmid DNA. Replication reactions were performed on both bead-coupled and soluble plasmid DNA. Detailed information can be found in the Supplemental Experimental Procedures.

## Author Contributions

C.F.K. performed all the experiments. J.T.P.Y. provided some proteins and helped with data analyses. H.P. analyzed nucleosome positioning data. A.E. helped with design and construction of the INO80 overexpression strain. C.F.K. and J.F.X.D. designed the experiments and wrote the paper.

## Supplemental Information

Document S1. Supplemental Experimental Procedures, Figures S1–S7, and Tables S1 and S2

Table S3. Chromatin-Associated Proteins Identified by Mass Spectrometry, Related to Figure 3 and Figure S3

Document S2. Article plus Supplemental Information
