# Supplementary material for: Chromatin Controls DNA Replication Origin Selection, Lagging-Strand Synthesis, and Replication Fork Rates
Source: Mol Cell. 2017 Jan 5;65(1):117–30. doi: 10.1016/j.molcel.2016.11.016 (PMC5222724; doi:10.1016/j.molcel.2016.11.016)
Supplement: Document S1. Supplemental Experimental Procedures, Figures S1–S7, and Tables S1 and S2 [file mmc1.pdf]

Molecular Cell, Volume *65*

Supplemental Information

Chromatin Controls DNA Replication Origin Selection, Lagging-Strand Synthesis, and Replication Fork Rates

Christoph F. Kurat, Joseph T.P. Yeeles, Harshil Patel, Anne Early, and John F.X. Diffley

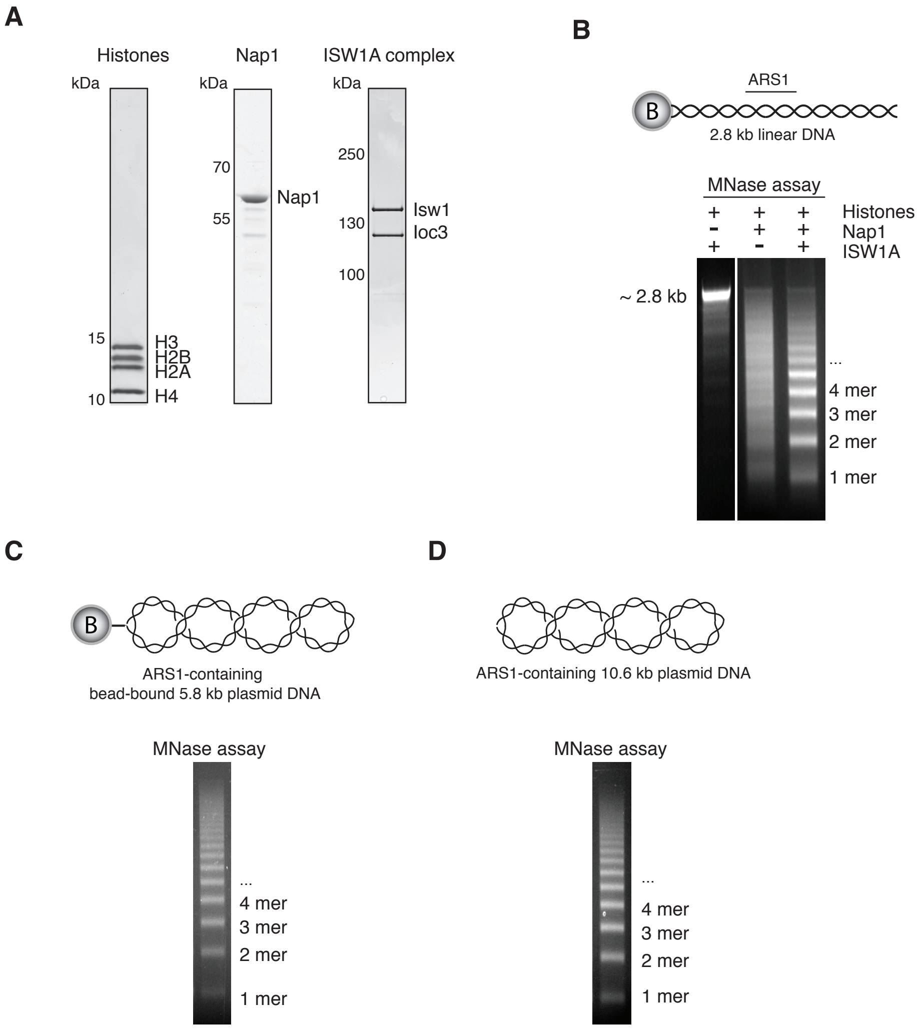
A
Histones
Nap1
ISW1A complex
kDa
15
10
H3
H2B
H2A
H4
70
55
Nap1
250
130
100
Isw1
Ioc3
B
ARS1
B
2.8 kb linear DNA
MNase assay
Histones
Nap1
ISW1A
~ 2.8 kb
...
4 mer
3 mer
2 mer
1 mer
C
B
ARS1-containing
bead-bound 5.8 kb plasmid DNA
MNase assay
...
4 mer
3 mer
2 mer
1 mer
D
ARS1-containing 10.6 kb plasmid DNA
MNase assay
...
4 mer
3 mer
2 mer
1 mer

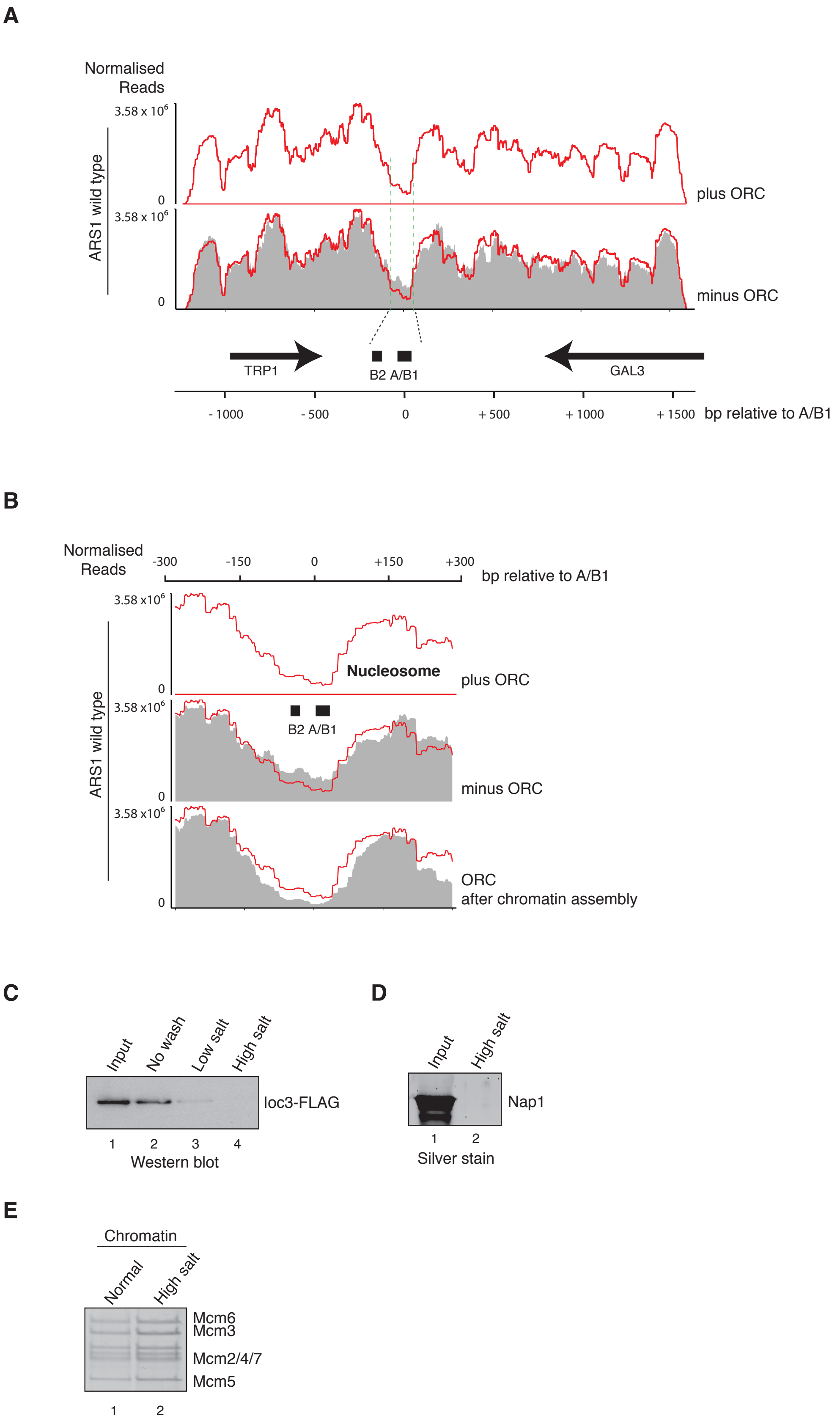
A
Normalised Reads
3.58 x 10^6
0
3.58 x 10^6
0
ARS1 wild type
plus ORC
minus ORC
TRP1
B2 A/B1
GAL3
- 1000
- 500
0
+ 500
+ 1000
+ 1500
bp relative to A/B1
B
Normalised Reads
-300
-150
0
+150
+300
bp relative to A/B1
3.58 x10^6
0
Nucleosome
plus ORC
3.58 x10^6
0
B2 A/B1
minus ORC
3.58 x10^6
0
ORC
after chromatin assembly
ARS1 wild type
C
Input
No wash
Low salt
High salt
Ioc3-FLAG
1
2
3
4
Western blot
D
Input
High salt
Nap1
1
2
Silver stain
E
Chromatin
Normal
High salt
Mcm6
Mcm3
Mcm2/4/7
Mcm5
1
2

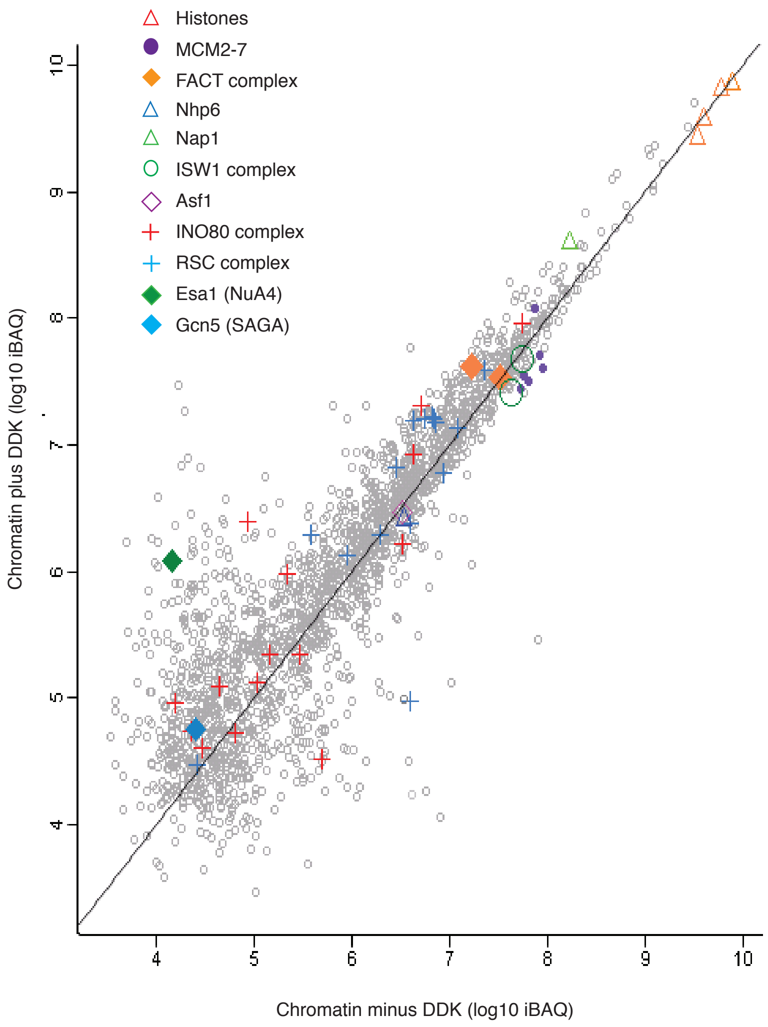
Histones
MCM2-7
FACT complex
Nhp6
Nap1
ISW1 complex
Asf1
INO80 complex
RSC complex
Esa1 (NuA4)
Gcn5 (SAGA)
Chromatin plus DDK (log10 iBAQ)
Chromatin minus DDK (log10 iBAQ)
4
5
6
7
8
9
10

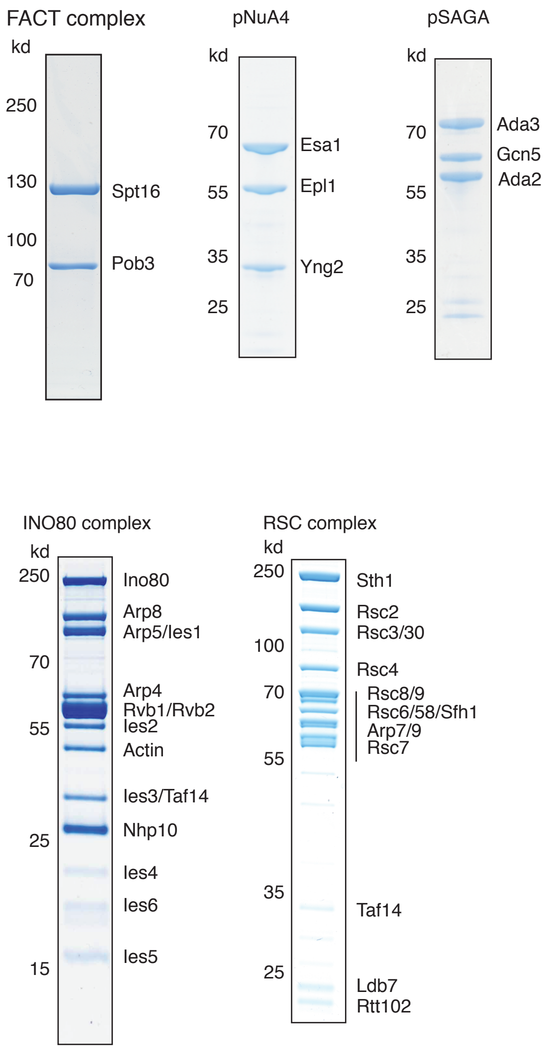
FACT complex
kd
250
130
100
70
Spt16
Pob3
pNuA4
kd
70
55
35
25
Esa1
Epl1
Yng2
pSAGA
kd
70
55
35
25
Ada3
Gcn5
Ada2
INO80 complex
kd
250
70
55
25
15
Ino80
Arp8
Arp5/Ies1
Arp4
Rvb1/Rvb2
Ies2
Actin
Ies3/Taf14
Nhp10
Ies4
Ies6
Ies5
RSC complex
kd
250
100
70
55
35
25
Sth1
Rsc2
Rsc3/30
Rsc4
Rsc8/9
Rsc6/58/Sfh1
Arp7/9
Rsc7
Taf14
Ldb7
Rtt102

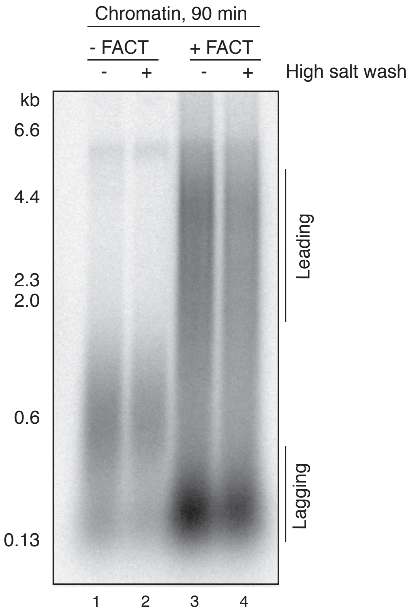

Chromatin, 90 min
- FACT
+ FACT
- + - +
High salt wash
kb
6.6
4.4
2.3
2.0
0.6
0.13
Leading
Lagging
1 2 3 4

A

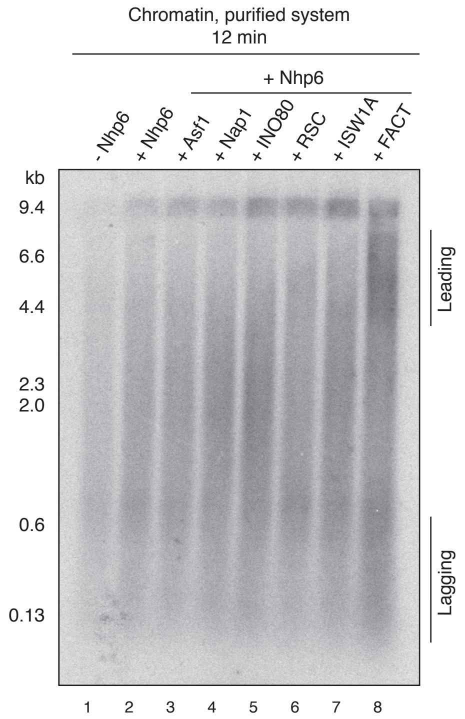
Chromatin, purified system
12 min
+ Nhp6
- Nhp6
+ Nhp6
+ Asf1
+ Nap1
+ INO80
+ RSC
+ ISW1A
+ FACT
kb
9.4
6.6
4.4
2.3
2.0
0.6
0.13
Leading
Lagging
1
2
3
4
5
6
7
8

B

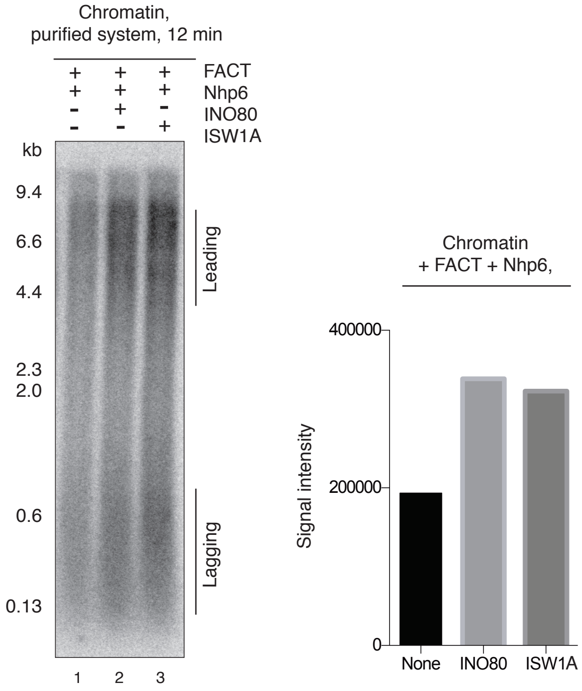
Chromatin,
purified system, 12 min
FACT
Nhp6
INO80
ISW1A
kb
9.4
6.6
4.4
2.3
2.0
0.6
0.13
Leading
Lagging
1
2
3
Chromatin
+ FACT + Nhp6,
Signal intensity
400000
200000
0
None
INO80
ISW1A

Kurat et al. Figure S7

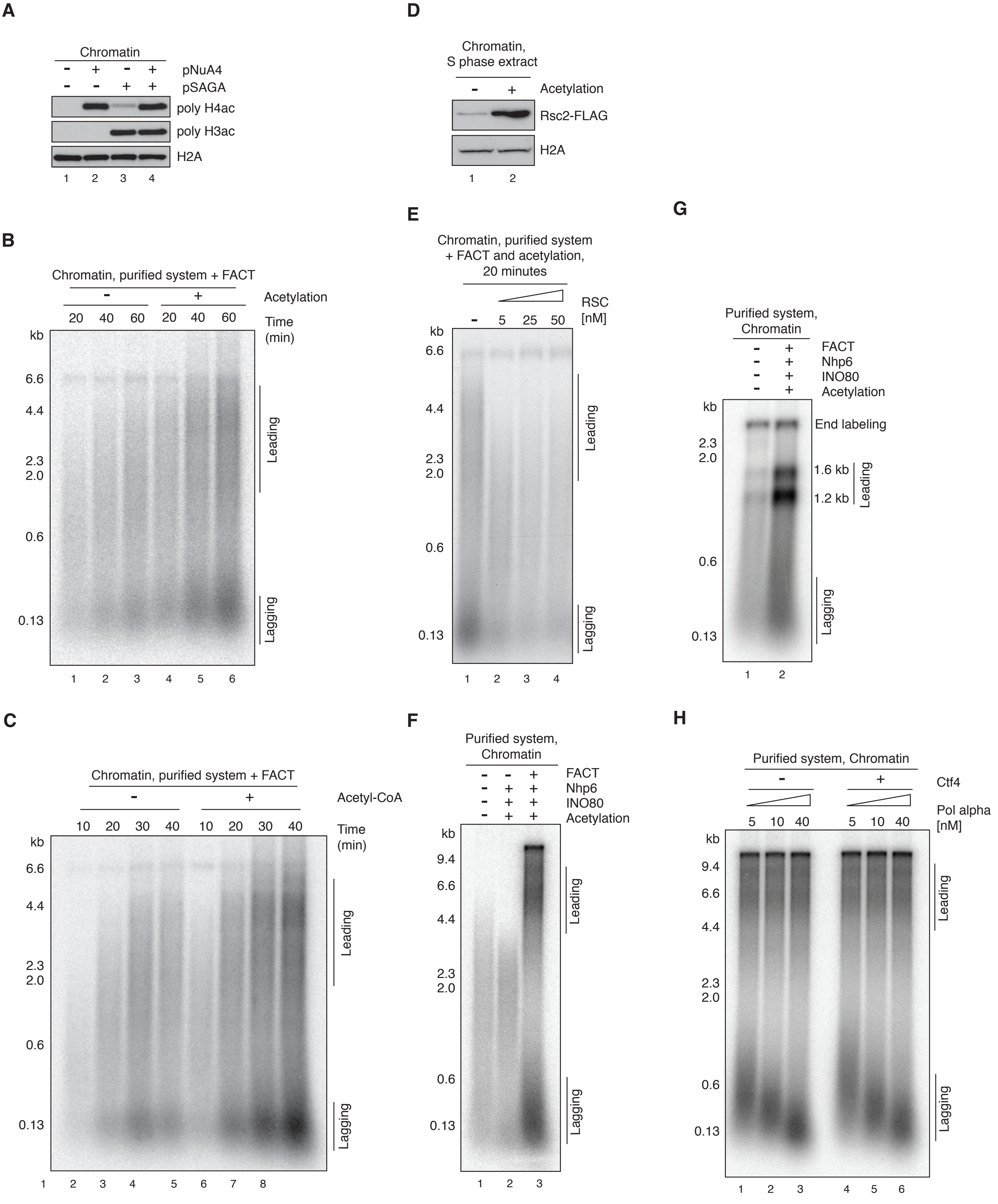

Supplemental Figures Legends

Figure S1. Related to all Figures. Chromatin assembly using different DNA substrates

(A) Purified yeast core histones, histone chaperone Nap1 and chromatin remodeler ISW1A were analyzed by SDS-PAGE and Coomassie (core histones and Nap1) or silver staining (ISW1A).

(B) Chromatin assembly on ARS1-containing 2.8 kb linear DNA coupled to paramagnetic beads. Reactions were performed in the presence or absence of Nap1 or ISW1A and treated with micrococcal nuclease (MNase) to examine efficiency of chromatin assembly.

(C) MNase assay showing chromatin assembly on a ARS1-containing 5.8 kb plasmid coupled to beads.

(D) Chromatin assembly on a 10.6 kb ARS1 plasmid in solution.

Figure S2. Related to Figure 1. Nucleosome profiles in the presence or absence of ORC

(A) Nucleosome positioning maps of the full-length ARS1-containing 2.8 kb fragment in the presence or absence of ORC are compared. Chromatin was assembled and MNase resistant nucleosomal DNA was prepared and sequenced as described in Experimental Procedures. Black bars show the relative location of the A/B1 and B2 elements. The red line is an overlay of the ARS1 wild type plus ORC experiment (first panel). Green lines indicate the relative position of the A1/B1 element of ARS1. The ARS1-flanking genes *TRP1* and *GAL3* are shown as black arrows.

(B) The top and middle panels show ARS1-adjacent nucleosomes from the experiment described in (A). The third panel shows an experiment where ORC was added after chromatin assembly. The red line is an overlay of the ARS1 wild type plus ORC experiment (first panel).

(C) Chromatin was assembled as in Figure S1 and ISW1A binding to chromatin was analyzed by immunoblotting after different washes (0.3 M K-acetate: low salt wash; 0.5 M NaCl: high salt wash). An antibody recognizing the FLAG epitope tag was used.

(D) Nap1 binding to chromatin was assessed by silver staining after a high salt wash (0.5 M NaCl).

(E) MCM loading on chromatin under standard conditions and after a high salt wash. Chromatin was assembled on 2.8 kb linear DNA in the presence of ORC and MCM was loaded as in Figure 1A (panel 1). Chromatin was assembled plus ORC and treated with a high salt wash (0.5 M NaCl) to remove ISW1 and Nap1 (Figure S5 A and B). After a low salt wash (0.3 M K-acetate), ORC, Cdc6 and MCM were added and loading reactions were performed as in Figure 1A.

Figure S3. Related to Figure 3 and Table S3. Mass spectrometry analyses of protein composition on chromatin

MCMs were loaded after chromatin assembly and either treated with DDK or left untreated before adding to an S phase extract. Histone chaperones (FACT, Nap1 and Asf1), Nhp6, chromatin remodelers (INO80, RSC and ISW1A), catalytic subunits of lysine acetyltransferases NuA4 (Esa1) and SAGA (Gcn5), the MCM complex and core histones are highlighted. Note that X- and the Y-axis are $\log_{10}$ transformed so that each unit represents a 10-fold difference in abundance. Protein quantification was performed using intensity-based quantification (iBAQ). For a complete list of all proteins identified see Table S3.

Figure S4. Related to Figure 3. Coomassie-stained SDS-PAGE analysis of multi-subunit complexes used in this study

Individual protein subunits from the polyacrylamide gel in Figure 3A are shown.

Figure S5. Related to Figure 3. Chromatin replication reaction after high salt wash

Replication reactions on chromatin in the presence or absence of the FACT complex plus and minus a high salt wash were performed as in Figure 2A.

Figure S6. Related to Figure 4. Stimulatory effect of Nhp6 on FACT

(A) Effects of histone chaperones and chromatin remodelers on chromatin replication in conjunction with Nhp6. Soluble replication reactions on chromatin were conducted as described in Figure 4A.

(B) Left panel - chromatin replication reactions showing the stimulatory effect of the INO80 and ISW1A together with FACT and Nhp6. Right panel – quantification of the replication reactions.

Figure S7. Related to Figure 4. Effects of histone acetylation and other factors on chromatin replication

(A) Chromatin was assembled on bead-coupled 5.8 kb plasmid as shown in Figure S1. Purified pNuA4, pSAGA (Figure 3A, Figure S4) and acetyl-CoA were added and acetylation was assessed using antibodies recognizing polyH3 and polyH4 acetylation. Detection of H2A was used as a loading control.

(B) Chromatin replication reactions on a bead-bound 5.8 kb plasmid were performed as shown in Figure 2A. Nucleosomes were acetylated with pNuA4 and pSAGA and replication reactions in the presence of FACT are shown at the time points indicated.

(C) Stimulatory effect of histone acetylation is dependent on acetyl-CoA. Chromatin was incubated with pSAGA and pNuA4 in the presence or absence of acetyl-CoA. Time courses of chromatin replication reactions were conducted as in (B).

(D) Recruitment of RSC in the presence and absence of histone acetylation using S phase extract. RSC recruitment was assessed using anti-FLAG antibody. Detection of H2A was used as a loading control.

(E) Chromatin replication reactions as shown in (B) with indicated amounts of RSC in the reaction.

(F) FACT is the major factor in chromatin replication. Soluble replication reactions on a 10.6 kb DNA template were performed as shown in Figure 4A with the indicated factors.

(G) Replication reactions on chromatin assembled on linear 2.8 kb DNA in the presence or absence of FACT, Nhp6, INO80 and histone acetylation. Because of the position of ARS1, two distinct leading strand products (1.2 and 1.6 kb) were observed.

(H) Ctf4 does not affect lagging strand size. Chromatin replication reactions were performed as shown in (F) in the presence or absence of Ctf4 and the indicated amounts of Pol α.

Supplemental Tables

Table S1. Related to all Figures. Yeast strains.

Strain	Genotype	Reference
yCFK1	*MATa ade2-1 ura3-1 his3-11,15 trp1-1 leu2-3,112 can1-100*<br>*bar1::Hyg*<br>*pep4::KanMX*<br>*IOC3*-3xFLAG-*NAT*	This study
yCFK2	*MATa ade2-1 ura3-1 his3-11,15 trp1-1 leu2-3,112 can1-100, cdc7-4,*<br>*his3::HIS3pRS303/SLD3-13MYC,*<br>*trp1::TRP1pRS304/SLD2,*<br>*leu2::LEU2pRS305/ SLD7,CDC45*	This study

	ura3::URA3pRS306/ DPB11<br>*Spt16-3xFLAG-NAT*	
yCFK3	*MATa ade2-1 ura3-1 his3-11,15 trp1-1 leu2-3,112 can1-100, cdc7-4,*<br>*his3::HIS3pRS303/SLD3-13MYC,*<br>*trp1::TRP1pRS304/SLD2,*<br>*leu2::LEU2pRS305/ SLD7,CDC45*<br>*ura3::URA3pRS306/ DPB11*<br>*Rsc2-3xFLAG-NAT*	This study
BCY211	Wittmeyer et al., 2004; Rsc2 tagged with a TAP tag at the C terminus	Kornberg lab
yAE84	*MAT**a** ade2-1 ura3-1 his3-11,15 trp1-1 leu2-3,112 can1-100*<br>*bar1::Hyg*<br>*pep4::KanMX*<br>*his3::HIS3pRS303/Ino80-TEV-3XFLAG, Nhp10*<br>*trp1::TRP1pRS304/Ies3, Ies4*<br>*leu2::LEU2pRS305/Ies5, Ies6*<br>*ura3::URA3pRS306/Ies1, Ies2*	This study
yAE85	*MATalpha ade2-1 ura3-1 his3-11,15 trp1-1 leu2-3,112 can1-100*<br>*bar1::Hyg*<br>*pep4::KanMX*<br>*his3::HIS3pRS303/Gal4, Taf14*<br>*trp1::TRP1pRS304/Rvb1, Rvb2*<br>*leu2::LEU2pRS305/Arp5, Arp8*<br>*ura3::URA3pRS306/Act1, Arp4*	This study

yAE86	*ade2-1 ura3-1 his3-11,15 trp1-1 leu2-3,112 can1-100*<br>*bar1::Hyg*<br>*pep4::KanMX*<br>*his3::HIS3pRS303/Ino80-TEV-3XFLAG, Nhp10*<br>*his3::HIS3pRS303/Gal4,Taf14*<br>*ura3::URA3pRS306/Ies1, Ies2*<br>*ura3::URA3pRS306/Act1,Arp4*<br>*leu2::LEU2pRS305/Ies5, Ies6*<br>*leu2::LEU2pRS305/Arp5, Arp8*<br>*trp1::TRP1pRS304/Ies3, Ies4*<br>*trp1::TRP1pRS304/Rvb1, Rvb2*	This study
yJH7	*MAT***a** *ade2-1 ura3-1 his3-11,15 trp1-1 leu2-3,112 can1-100*<br>*bar1::Hyg*<br>*pep4::KanMX*<br>*his3::HIS3pRS303/Asf1-CBP,Gal4*	This study

Table S2. Related to all Figures. Plasmids.

Plasmid	Plasmid construction	Reference
pCFK1	*NAP1* was cloned as a 5'-BamHI and 3'-NotI fragment into pGEX-6p-1 (a gift from Tim Hunt)	This study
pRS303-Ino80A	Ino80-Tev-3xFLAG-Gal-Nhp10 (starting plasmid yJF2 (for all yJF plasmids see Coster et al., 2014; Frigola et al., 2013)).<br>Ino80 was cloned with SgrAI and NotI, Nhp6 was cloned with AscI and XhoI. All subsequent genes left	This study

	to the GAL promoter were cloned with SgrAI and NotI and all genes right to GAL with AscI and XhoI.	
pRS303-Ino80B	Taf14-Gal-Gal4 (starting plasmid yJF2).	This study
pRS304-Ino80A	Ies3-Gal-Ies4 (starting plasmid pJF3).	This study
pRS304-Ino80B	Rvb2-Gal-Rvb1 (starting plasmid pJF3).	This study
pRS305-Ino80A	Ies5-Gal-Ies6 (starting plasmid pJF4)	This study
pRS305-Ino80B	Arp5-Gal-Arp8 (starting plasmid pJF4)	This study
pRS306-Ino80A	Ies1-Gal-Ies2 (starting plasmid pJF5)	This study
pRS306-Ino80B	Act1-Gal-Arp4 (starting plasmid pJF5)	This study
pRS303-Asf1	Asf1 cloned between SgrAI and NotI sites	This study
pBP83	Frigola et al., 2013	Diffley lab
pCDFduet.H2A-H2B	Kingston et al., 2011	Singleton lab
pETduet.H3-H4	Kingston et al., 2011	Singleton lab
pRJ1228-Nhp6	Ruone et al., 2003	Formosa lab
pTF175	Biswas et al., 2005	Formosa lab

pJW22	Biswas et al., 2005	Formosa lab
pST44-yAda3D2HIS-yAda3D1-Gcn5	Barrios et al., 2007	Tan lab
pST44-HISyEsa1-yEpl1D3-Yng2D2	Barrios et al., 2007	Tan lab

Table S3. Related to Figures 3 and S3. Chromatin associated proteins identified by mass spectrometry.

Supplemental Experimental Procedures

Yeast strains

Strains used in this study were constructed by transforming strain yJF1 with linearized plasmids or PCR products using standard genetic methods (details on strains and vectors see Table S 1 and 2). For generation of ISW1A expression strain, a 3xFLAG tag was chromosomally inserted at the C-terminus of Ioc3 in yJF1 using pBP83 as a template.

For INO80, codon optimized versions of the 15 subunits of INO80 were cloned in pairs into one of four versions of a yeast expression vector containing a bidirectional GAL promoter (pJF2-5, see Table S2 for details). The exception was Taf14, which was paired with Gal4. Ino80 was tagged at the C-terminus with a codon-optimized version of the 3xFLAG tag. For each of the four markers, two expression plasmids were created, which were transformed sequentially either into a MAT alpha or MAT a version of yJF1. The two final haploid strains, each expressing eight proteins, were

mated to produce the diploid INO80 expression strain. The synthetic constructs were codon optimised and synthesized as described in the accompanying manuscript.

Proteins

All proteins purified in this study, except yeast core histones and Nhp6, had affinity tags, which were used for the first step of purification. Nap1, Nhp6, pNuA4 and pSAGA were expressed in in *E. coli* BL-21 CodonPlus (DE3)-RIL. ISW1A and RSC were expressed from yeast under control of the endogenous promoters. INO80 and FACT subunits as well as Asf1 were overexpressed from a *GAL1-10* promoter in a yJF1 background. For FACT, yJF1 harbouring 2 plasmids for expression of the two subunits of FACT (see Table S 2) were grown in yeast minimal medium containing 2% sucrose as the carbon source. Expression was induced by the addition of 2 % galactose for 16 hours.

ORC, Cdc6, Cdt1-Mcm2-7, DDK, Sld2, Sld3/7, GINS, Cdc45, Dpb11, Ctf4, RPA, Topo I, Pol ε, Pol α, S-CDK, RSC, pNuA4 and pSAGA were expressed and purified as described previously (Barrios et al., 2007; Wittmeyer et al., 2004; Yeeles et al., 2015b). Mrc1, Csm3-Tof1, Pol δ, RFC and PCNA were purified as described in the accompanying manuscript.

Protein purifications

ORC, Cdc6, Cdt1-Mcm2-7, DDK, Sld2, Sld3/7, GINS, Cdc45, Dpb11, Ctf4, RPA, Topo I, Pol ε, Pol α, S-CDK, RSC, pNuA4 and pSAGA were expressed and purified as described previously (Barrios et al., 2007; Wittmeyer et al., 2004; Yeeles et al., 2015). Mrc1, Csm3-Tof1, Pol δ, RFC and PCNA were purified as described in the accompanying manuscript.

Histone purification.

Yeast core histones were expressed and purified as described previously (Kingston et al., 2011) with modifications. Pellets were slowly thawed on ice before adding 0.5 M NaCl, 20 mM Tris-HCl pH 8, 0.1 mM EDTA, 10 mM β-Mercaptoethanol and EDTA-free Protease Inhibitor Tablets (cOmplete, Roche). Cells were broken by sonication (2 minutes, 40 % output) and insoluble material was collected by centrifugation (235.000g, 4˚C, 45 minutes). Supernatant was passed over a 5ml HiTrap Heparin column (GE Healthcare) and eluted with 20 column volumes (CV) of a 0 M to 2 M NaCl gradient (20 mM Tris-HCl pH 8, 0.1 mM EDTA, 10 mM β-Mercaptoethanol, no protease inhibitors). Peak fractions were collected, concentrated and passed over a Superdex 200 column (GE Healthcare) using 2 M NaCl buffer ((20 mM Tris-HCl pH 8, 0.1 mM EDTA, 10 mM β-Mercaptoethanol, no protease inhibitors). Peak fractions were pooled and concentrated.

Nap1 purification.

Cell pellets were slowly thawed on ice before adding an equal amount of Nap1 lysis buffer (50 mM $KxPO_4$ pH 7.6, 150 mM KOAc, 5 mM $MgCl_2$, 1% Triton, 1 mM DTT and EDTA-free Protease Inhibitor Tablets (cOmplete, Roche, 1 tablet per 50 ml lysis buffer)). Cells were broken and centrifuged as described for histone purification. 1 ml glutathione agarose slurry was washed with Nap1 lysis buffer, before adding the cleared lysate for 2 hours at 4 ˚C followed by transfer into a disposable column. Beads were washed extensively with Nap1 lysis buffer without protease inhibitors. An equal amount of lysis buffer was added to beads to generate 50% slurry. To cleave Nap1 from GST, 50 ml Prescission protease (GE Healthcare) was added for 2 hours at 4 ˚C. Eluate was collected, dialyzed into 20 mM Tris-HCl ph 7.5, 100 mM NaCl, 0.5 mM EDTA, 10 % glycerol, 1 mM DTT and 0.1 mM PMSF) and Nap1 was further purified using a 1 ml MonoQ column (GE Healthcare). Nap1 was eluted using a 20

CV gradient from 0.1 M to 1 M NaCl (20 mM Tris-HCl ph 7.5, 100 mM NaCl, 0.5 mM EDTA, 10 % glycerol and 1 mM DTT). Peak fractions were pooled and concentrated.

ISW1A purification.

ISW1A was purified as previously described with modifications (Vary et al., 2004). Cell pellets were resuspended in an equal volume of lysis buffer (25 mM HEPES pH 7.6, 0.1 mM EDTA, 2 mM $MgCl_2$, 20 % glycerol, 0.02 % NP40, 1 mM DTT and 300 mM KCl) with 1x protease inhibitors (0.3 mM PMSF, 7.5 mM benzamidine, 0.5 mM AEBSF, 1 mM pepstatin A, 1 mg/ml aprotinin (Sigma) and 1 mM leupeptin (Merck)). Insoluble material was cleared as above (235.000g, 4˚C, 45 minutes) and Ioc3-FLAG was bound to pre-washed anti-FLAG M2 affinity gel (Sigma) in batch 1 hour at 4 ˚C. After transferring into a disposable column, beads were washed with 20 CV of lysis buffer containing 300 mM KCl followed by 20 CV of lysis buffer containing 100 mM KCl. ISW1 was eluted in 1 CV of lysis buffer containing 100 mM KCl with 0.5 mg/ml 3x FLAG peptide, followed by 2 CV of lysis buffer containing 100 mM KCl with 0.25 mg/ml 3x FLAG peptide.

The eluates were pooled and applied to a 1 ml MonoQ column. ISW1 was eluted with a 15 CV gradient from 200 mM KCl to 600 mM KCl in lysis buffer. Peak fractions were pooled and concentrated.

Asf1 purification.

For purification of Asf1-CBP, frozen cell pellets were thawed slowly on ice. An equal amount of CBP lysis buffer (30 mM HEPES pH 7.6, 100 mM KCl, 10% glycerol, 5 mM DTT, 0.1% NP-40 and EDTA-free Protease Inhibitor Tablets) was added and insoluble material was removed by centrifugation (235.000 g, 4˚C, 1 hour). Supernatant was added to pre-washed Calmodulin affinity resin (Agilent) together with $CaCl_2$ to 2 mM and rotated for 2 hours at 4 ˚C. After transferring into a 20 ml disposable column, beads were washed extensively with wash buffer (30 mM

HEPES pH 7.6, 100 mM KCl, 10% glycerol, 5 mM DTT, 0.1% NP-40 and 2 mM $CaCl_2$). Asf1-CBP was eluted with 10x 1ml buffer containing 30 mM HEPES pH 7.6, 100 mM KCl, 10% glycerol, 5 mM DTT, 0.1% NP-40, 2 mM EDTA and 2 mM EGTA. The eluates were pooled and applied to a 1 ml MonoQ column. Asf1-CBP was eluted with a 30 CV gradient from 0 to 1 M KCl (0/1 M KCl, 30 mM HEPES pH 7.6, 10% glycerol, 1 mM DTT and 0.5 mM EDTA). Peak fractions were pooled and dialyzed into buffer containing 30 mM HEPES pH 7.6, 100 mM KCl, 10% glycerol, 5 mM DTT and 0.1% NP-40.

FACT purification.

FACT was purified as previously described (Biswas et al., 2005) with modifications. 16 g of cell pellets were resuspendend in 8 ml buffer containing 20 mM Tris-HCl pH 8 and 5 mM Imidazole and dropped into liquid nitrogen to make popcorn. Popcorn was then manually ground in a pre-cooled mortar with constant addition of liquid nitrogen. Cell powder was collected, thawed on ice (24 ml) and 16 ml buffer (20 mM Tris-HCl pH 8, 5 mM Imidazole, 0.5 mM NaCl and EDTA-free Protease Inhibitor Tablet) was added to give a total volume of 40ml. Because there was no NaCl in the cell-resuspension buffer, 2.4 ml of 5 M NaCl was added to give a final concentration of 0.5 M NaCl. After a first centrifugation step (12.000 g, 10 minutes, 4 ˚C), supernatant was collected and insoluble material was removed by a second centrifugation step (235.000 g, 4˚C, 30 minutes). The supernatant was added to 8 ml of pre-washed Talon Metal Affinity resin (Clontech). Beads were gently rotated in batch for 75 minutes at 4˚C. Beads were then washed with 2x 50 ml buffer (20 mM Tris-HCl pH 8, 5 mM Imidazole and 0.5 mM NaCl) before transferring into a 20 ml disposable column. Beads were washed with additional 40 ml buffer (20 mM Tris-HCl pH 8, 0.5 M NaCl and 20 mM Imidazole) before FACT was eluted with 8 x 1ml buffer E1 (20 mM Tris-HCl pH 8, 0.5 M NaCl and 100 mM Imidazole), 2 x 1ml buffer E2 (20 mM Tris-HCl pH 8, 0.5 M NaCl and 500 mM Imidazole) and 2 x 1 ml buffer E3 (20 mM

Tris-HCl pH 8, 0.5 M NaCl and 1 M Imidazole). Desired fractions were concentrated and loaded onto Superdex S200 column, which was equilibrated with buffer containing 20 mM Tris-HCl pH 7.5, 200 mM NaCl, 10 % glycerol and 1 mM β-Mercaptoethanol.

Nhp6 purification.

Nhp6 was purified as previously described with modifications (Ruone et al., 2003). Cell pellets were thawed on ice and an equal amount of lysis buffer was added (20 mM Tris-HCl pH 7.5, 500 mM NaCl, 2 mM EDTA, 10% glycerol and 1 mM β-Mercaptoethanol). Cells were lysed by sonication (2 minutes, 40 % output) and supernatant was collected by centrifugation (12.000 g, 10 minutes, 4 ˚C) before pouring the supernatant into an Erlenmeyer flask with a magnetic stir bar. The volume of 50% trichloroacetic acid (TCA) to add was determined by multiplying the volume of the supernatant by 0.0417 (2 % final concentration of TCA). TCA was added slowly while stirring to prevent precipitation of Nhp6 (unlike Nhp6, most other proteins precipitate at 2 % TCA). Stirring was continued for 30 minutes at 4 ˚C. The solution was distributed to polypropylene centrifuge tubes and centrifuged (39.000 g, 30 minutes, 4˚C). The supernatant was pooled into a graduated cylinder to get an accurate measurement of the volume before pouring into an Erlenmeyer flask with a magnetic stir bar. TCA was added to a final concentration of 10 % to precipitate Nhp6. Stirring was continued for 30 minutes at 4 ˚C. Precipitated material was collected by a centrifugation step (39.000 g, 30 minutes, 4˚C). The pellet was washed with acetone, vortexed and centrifuged for 5 minutes (30.000 g, 4 ˚C). Pellets were dried under vacuum and dissolved in buffer containing 20 mM Tris-HCl pH 7.5, 300 mM NaCl, 1 mM EDTA, 10 % glycerol and 1 mM β-Mercaptoethanol and dialyzed against the same buffer twice for at least 3 hours each time. After filtering through a 0.2mM syringe filter, the dialyzed sample was applied to a MonoS column

(GE Healthcare) and eluted with a 35 ml gradient from 300 to 1400 mM NaCl (20 mM Tris-HCl pH 7.5, 1 mM EDTA, 10 % glycerol and 1 mM β-Mercaptoethanol). Desired fractions were pooled and dialyzed against 600 ml of buffer containing 200 mM KOAc, 1 mM β-Mercaptoethanol, 25 mM HEPES pH 7.6 and 10 % glycerol.

INO80 purification.

INO80 was purified as previously described with modifications (Shen, 2004). Pellets were thawed slowly on ice before adding an equal amount of lysis buffer (25 mM HEPES pH 7.6, 500 mM KCl, 10 % glycerol, 0.05% NP-40, 1 mM EDTA, 1 mM DTT, 4 mM $MgCl_2$ and protease inhibitors (0.3 mM PMSF, 7.5 mM benzamidine, 0.5 mM AEBSF, 1 mM leupeptin, 1 mM pepstatin A and 1 mg/ml aprotinin)). Soluble material was collected by centrifugation (80.000 g, 2 hours, 4˚C). An equal amount of lysis buffer plus protease inhibitors was added to the supernatant and applied to anti-FLAG M2 affinity gel (Sigma) in batch for 1 hour at 4 ˚C. Beads were washed extensively with 25 mM HEPES pH 7.6, 500 mM KCl, 10 % glycerol, 0.05% NP-40, 1 mM EDTA, 1 mM DTT, 4 mM $MgCl_2$, and then washed with 25 mM HEPES pH 7.6, 200 mM KCl, 10 % glycerol, 0.05% NP-40, 1 mM EDTA, 1 mM DTT, 4 mM $MgCl_2$. INO80 was eluted by 1 CV of the same buffer with 0.5 mg/ml 3x FLAG peptide, followed by 2 CV of buffer with 0.25 mg/ml 3x FLAG peptide. The elutate was then dialyzed into 200 mM KOAc, 25 mM Tris-HCl pH 7.2, 10 % glycerol, 1 mM DTT, 0.05 % NP-40 and 4 mM $MgCl_2$ before applying to a MonoQ column, which was equilibrated in a buffer containing 100 mM KCl, 25 mM Tris-HCl pH 7.2, 10 % glycerol, 1 mM DTT, 0.05 % NP-40 and 4 mM $MgCl_2$. After running a gradient from 100 mM to 300 mM KCl for 10 CV, INO80 was stepped-off with a short gradient of 2 CV from 300 to 600 mM KCl.

INO80 was then dialyzed into buffer containing 100 mM NaCl, 25 mM Tris-HCl pH 7.2, 1 mM DTT, 10 % glycerol and 1 mM EDTA.

Chromatin assembly and MCM loading on bead-coupled linear DNA

Chromatin assembly was carried out as described previously (Vary et al., 2004). For chromatin assembly and MCM loading on bead-coupled linear DNA, a 2.8 kb fragment of yeast DNA containing ARS1 wild type and ars1 $A^{-}B2^{-}$ mutant was prepared as described (Yeeles et al., 2015). Highly saturated chromatin was assembled in buffer containing 10 mM HEPES pH 7.6, 50 mM KCl, 5 mM $MgCl_2$, 0.5 mM EGTA, 10 % glycerol and 0.1 mg/ml BSA. Yeast histones (370 nM), Nap1 (3.5 μM) and ISW1 (7 nM) were combined and left on ice for 30 minutes. Then, creatine phosphate (40 mM), ATP (3mM), creatine phosphate kinase (0.6 ml of a 14 mg/ml stock solution) and bead coupled DNA (500 ng) were added and incubated at 30 ˚C with shaking (1250 rpm). After 10 minutes, ORC (20 nM, unless stated otherwise) was added and the reaction was incubated for additional 3 hours and 50 minutes. Naked DNA was treated exactly as chromatin, without addition of histones, Nap1 and ISW1. To get origin specificity, beads were washed twice with 200 ml of low salt buffer (45 mM HEPES-KOH pH 7.6, 5 mM $Mg(OAc)_2$, 0.02 % NP-40, 10 % glycerol and 0.3 M KOAc). Loading buffer (25 mM HEPES-KOH pH 7.6, 10 mM $Mg(OAc)_2$, 90 mM KOAc, 1 mM DTT, 0.1 % NP-40 and 5 % glycerol), Cdt1-Mcm2-7 (50 nM), Cdc6 (80 nM) and ATP (2 mM) were added to the reaction and incubated for 30 minutes at 30 ˚C with shaking. Beads were washed with high (as low salt buffer, just 0.5 M NaCl instead of 0.3 M KOAc) or low salt buffer, naked DNA and chromatin bound fractions were released from beads with addition of MNase (NEB, 2000 units, 37 ˚C, 5 minutes) and analysed by immunoblotting or silver staining.

Nucleosome positioning

Chromatin was assembled plus and minus ORC (30 nM). MNase sequencing was carried out on the Illumina HiSeq 2500 platform and typically generated ~40 million 51bp paired-end reads per sample. Alignments were performed using bwa (version

0.7.7-r441; (Li and Durbin, 2009)) with the following parameters; -l=51 -k=2 -n=2. Alignments were filtered to include concordantly mapped read pairs with an insert size between 110-160bp. Per base sample coverage was calculated by treating each pair of reads as a single fragment, including the inferred insert portion. A median mapped library size of 40 million read pairs was used to normalise the coverage for cross-sample comparison.

S phase extracts

Spt16-FLAG (FACT complex) was depleted from an yCFK2 extract by 3 rounds (60 minutes each) of incubation at 4˚C with a 20 % volume of anti-FLAG M2 magnetic beads (Sigma). A mock sample was incubated for the same time with shaking at 4 ˚C. Total protein concentrations of both samples were measured using Bradford and the mock sample was diluted using 50 mM HEPES-KOH pH 7.6, 5 mM $Mg(OAc)_2$, 300 mM K-glutamate, 1 mM EDTA, 1 mM EGTA, 10 % glycerol and 3 mM DTT to adjust total protein concentrations. Recruitment of RSC to chromatin was detected by incubation with an S phase extract harbouring Rsc2-FLAG (yCFK3). Chromatin was acetylated as described below.

CMG recruitment assays

For CMG recruitment reactions, ARS1 containing circular plasmids coupled to beads were generated as described previously (Yeeles et al., 2015a). Chromatin assembly and MCM loading were executed as described before using 500 ng bead-coupled DNA. On bead bound circular plasmids, MCM loading on chromatin was less than on naked DNA, presumably because MCM loading on naked DNA was not origin specific. Therefore MCM levels had to be adjusted. For all subsequent experiments (recruitment and replication assays) involving naked DNA 15 nM Cdt1-Mcm2-7 (instead of 50 nM for chromatin) were used for the loading step. Loaded MCMs were then incubated with or without 100 nM DDK at 25 ˚C for 30 minutes. For reactions

using S phase extract, 10 ml of loaded MCMs, 10 ml of S phase extract and 15 ml of buffer containing 13 mM $MgCl_2$, 60 mM HEPES-KOH pH 7.6, 7 mM ATP, 4 nM DTT, 60 mM creatine phosphate, 1 ml creatine phospho kinase (14 mg/ml stock solution), 40 μM dATP, dCTP, dGTP, dTTP and 100 μM CTP, GTP, UTP were added. Reactions were incubated at 30 ˚C for 30 minutes with shaking. Beads were washed twice in TE buffer pH 8 and then twice with high salt buffer containing 45 mM HEPES-KOH pH 7.6, 5 mM $Mg(OAc)_2$, 0.02 % NP-40, 10 % glycerol and 0.3 M KCl. Naked DNA and chromatin bound fractions were released from beads by the addition of MNase and analysed by immunoblotting. For recruitment assays using purified proteins, MCM loading and DDK treatment were executed as described above. Beads were then collected, washed with low salt buffer and reactions were started by adding a master mix containing 40 mM HEPES-KOH pH 7.8, 250 mM K-glutamate, 5 % glycerol, 5 mM ATP, 10 mM $Mg(OAc)_2$, 2 mM DTT, 400 mg/ml BSA, 0.02 % NP-40, 40 nM Sld3/7, 60 nM Sld2, 40 nM Cdc45, 30 nM Dpb11, 30 nM Pol ε, 210 nM GINS, 50 nM S-CDK and 5 nM Mcm10. Reactions were incubated at 30 ˚C with shaking for 10 minutes before treated as described above. CMG recruitment was assessed by immunoblotting.

Replication assays on bead bound plasmid DNA templates

Replication assays using bead coupled circular ARS1 plasmid (Yeeles et al., 2015a) and S phase extract were identical to CMG recruitment assays with the exception of adding 80 nM of [a-^{32}P] -dCTP to the reaction. Replication reactions were terminated by removing the supernatant and washing the twice beads in TE buffer pH 8. Beads were resuspended in 5 mM EDTA, and NaOH (50 mM) and sucrose (1 % w/v) were then added. Beads were incubated at 30 ˚C for 30 minutes with shaking. Replication products were separated through 0.8 % alkaline agarose gels in 30 mM NaOH and 2 mM EDTA for 16 hours at 26 V. After fixing with 5 % cold trichloroacetic acid for 20

minutes twice, gels were dried onto Whatman papers and autoradiographed with Amersham Hyperfilm-MP (GE Healthcare) or scanned using a Typhoon phosphoimager (GE Healthcare). Quantification was executed using ImageQuant software.

For replication reactions using bead bound plasmid DNA and purified proteins, chromatin assembly, MCM loading and DDK phosphorylation were conducted as described above. After collecting the beads and removing the supernatant, beads were washed twice in buffer containing 45 mM HEPES-KOH pH 7.6, 5 mM $Mg(OAc)_2$, 0.02 % NP-40, 10 % glycerol and 0.3 M KOAc. Replication was initiated by adding a replication mix containing 25 mM Hepes KOH pH 7.6, 200 mM K-glutamate, 10 mM $Mg(OAc)_2$, 100 μg/ml BSA, 1 mM DTT, 0.01% NP-40, 3 mM ATP, 200 μM CTP, GTP, UTP, 40 μM dCTP, dGTP, dATP, dTTP, 60 nM [α^{32}P]-dCTP, 20 nM Ctf4, 30 nM Dpb11, 210 nM GINS, 40 nM Cdc45, 20 nM Pol ε, 5 nM Mcm10, 50 nM Sld2, 25 nM Sld3/7, 20 nM Pol α, 10 nM Topo I, 100 nM RPA, 20 nM S-CDK, 20 nM Csm3/Tof1, 10 nM Mrc1, 10 nM Pol δ, 20 nM RFC and 20 nM PCNA. For chromatin replication reactions, FACT was added at 40 nM unless stated otherwise. For chromatin replication reactions using acetylated nucleosomes as a template, 20 mM acetyl CoA, 300 nM pNuA4 and 300 nM SAGA were added after the DDK step and incubated for 30 minutes at 30 ˚C with shaking.

Samples were treated and analysed as described above.

Soluble replication assays

All reactions were incubated at 30 ˚C without shaking. ARS1 containing 10.6 kb plasmids were generated as described in the accompanying manuscript. Chromatin assembly reactions in the presence of ORC were performed as described before with 1.3 nM plasmid DNA as the template and in a buffer containing 25 mM HEPES-KOH pH 7.6, 10 mM $Mg(OAc)_2$, 100 mM KOAc, 0.1 % NP-40, 5 % glycerol and 0.1 mg/ml

BSA. Prior to MCM loading, ORC containing chromatinised circles were then put over a gel filtration column (MicroSpin S-400 HR Column, GE Healthcare), which was equilibrated three times with 250 ml buffer containing 100 mM K-glutamate, 25 mM HEPES, 10 mM $Mg(OAc)_2$, 0.02 % NP-40 and 1 mM DTT. For MCM loading, 2 mM ATP, 50 mM Cdt1-Mcm2-7 and 80 mM Cdc6 were added and incubated at 30 ˚C for 30 minutes. After a DDK step (50 nM, 30 ˚C for 30 minutes), chromatin was acetylated if indicated (20 mM acetyl CoA, 300 nM pNuA4 and 300 nM SAGA; 30 ˚C for 30 minutes). For replication, 10 ml of a master-mix containing 300 mM K-glutamate, 25 mM Hepes KOH pH 7.6, 10 mM $Mg(OAc)_2$, 100 μg/ml BSA, 1 mM DTT, 0.01% NP-40, 3 mM ATP, 400 μM CTP, GTP, UTP, 80 μM dCTP, dGTP, dATP, dTTP, 60 nM [$\alpha^{32}P$]-dCTP, 20 nM Ctf4, 30 nM Dpb11, 210 nM GINS, 40 nM Cdc45, 20 nM Pol ε, 5 nM Mcm10, 50 nM Sld2, 25 nM Sld3/7, 20 nM Pol α (except Pol α titration experiments), 10 nM Topo I, 100 nM RPA, 20 nM S-CDK, 20 nM Csm3/Tof1, 10 nM Mrc1, 10 nM Pol δ, 20 nM RFC and 20 nM PCNA was added to 10 ml of a MCM loading reaction. This resulted in final concentrations of 200 mM K-glutamate, 200 μM CTP, GTP, UTP and 40 μM dCTP, dGTP, dATP, dTTP. For chromatin replication reactions unless stated otherwise, FACT was added at 40 nM and Nhp6 was added at 400 nM. When indicated, 40 nM INO80, 40 nM ISW1A, 30 nM Asf1, 30 nM Nap1 and 40 nM RSC were added. To stop the reactions, 30 mM ETDA was added and unincorporated nucleotides were removed using Illustra MicroSpin G-50 columns (GE Healthcare). Samples were separated through 0.8% alkaline agarose gels as described before.

For soluble reactions using naked DNA, MCMs were loaded in a buffer containing 1.3 nM ARS1 10 kb plasmid DNA, 15 nM Cdt1-Mcm2-7, 20 nM ORC, 80 nM Cdc6, 100 mM K-glutamate, 25 mM HEPES-KOH pH 7.6, 10 mM $Mg(OAc)_2$, 5 mM ATP, 0.02 % NP-40 and 1 mM DTT. Reactions were incubated for 30 minutes at 30 ˚C before DDK (50 nM) was added and incubated for another 30 minutes at 30˚C.

Replication reactions were started by adding the same master-mix as described for soluble reactions on chromatin, the only exception being 400 mM K-glutamate in the buffer, which resulted in a final concentration of 250 mM after combining with loaded MCMs. When indicated, 40 nM FACT and 400 nM Nhp6 were added to the reaction. Reactions were treated as described above. For MNase assays in the presence of chromatin modifying proteins, chromatin was made as described, purified over a gel filtration column. When indicated, chromatin was acetylated as described and incubated with indicated factors for 7 minutes at 30 ˚C. MNase assay was executed as described before.

Data analyses

All gels were scanned using a typhoon phosphorimager. For pulse-chase experiments the positions of the peaks were assigned manually. To obtain replication rates, data were processed as described in the accompanying manuscript.

Antibodies

Spt16-FLAG and Ioc3-FLAG were visualised using anti-FLAG M2 peroxidase (Sigma). Psf1 antibody was a gift from K. Labib. Antibody against Cdc45 was described (On et al., 2014). Anti-Mcm7 was from Santa Cruz (yN-19, sc 6688) and anti-ORC6 from S. Bell (SB49). To detect acetylation, anti-acetyl histone H3 and H4 were used respectively (Millipore 06-599 and 06-866) were used. Anti H2A was used as a loading control (Active Motif, 39235).

DNA primers

Ioc3-F:

ACGACAATGATTCTTCTTTTGATGATGGTAGAGTTAAAAGGCAGCGCACTCTGG AAGTGCTGTTTCAGGGCCCGCGTACGCTGCAGGTCGAC

Ioc3-R:

GCCTGTAAGGAGTTTCACAATCTTCACGTTCGTTGAAAGCTAGTTGTCTAATCGA

TGAATTCGAGCTCG

Spt16-F:

AATTAGAGAAAAAGGCTGCTAGGGCTGATAGGGGTGCAAACTTTAGAGATCGTA

CGCTGCAGGTCGAC

Spt16-R:

TTCTGTCAGATCAAGGTCTTGCTGGTGAAACCCAGTAAGTGTTATAAAGTATCGA

TGAATTCGAGCTCG

Rsc2-F:

AGTTCACGGCGCACAGACTCTCTATGCTGCGGCCTCCTTCGTCGTCTTCACGTA

CGCTGCAGGTCGAC

Rsc2-R:

ATGCGCAATGGGAAGATATTATGCTGCCATTGCTTTTACAATAAAGGTGAATCGA

TGAATTCGAGCTCG

Nap1_BamHI-F: CGATGGATCCTCAGACCCTATCAGAACGAAACC

Nap1_NotI-R: CGATGCGGCCGCTTATGACTGCTTGCATTCAGGAG

Supplemental References

Barrios, A., Selleck, W., Hnatkovich, B., Kramer, R., Sermwittayawong, D., and Tan, S. (2007). Expression and purification of recombinant yeast Ada2/Ada3/Gcn5 and Piccolo NuA4 histone acetyltransferase complexes. Methods *41*, 271-277.

Biswas, D., Yu, Y., Prall, M., Formosa, T., and Stillman, D.J. (2005). The yeast FACT complex has a role in transcriptional initiation. Mol Cell Biol *25*, 5812-5822.

Kingston, I.J., Yung, J.S., and Singleton, M.R. (2011). Biophysical characterization of the centromere-specific nucleosome from budding yeast. J Biol Chem *286*, 4021-4026.

Li, H., and Durbin, R. (2009). Fast and accurate short read alignment with Burrows-Wheeler transform. Bioinformatics *25*, 1754-1760.

Ruone, S., Rhoades, A.R., and Formosa, T. (2003). Multiple Nhp6 molecules are required to recruit Spt16-Pob3 to form yFACT complexes and to reorganize nucleosomes. J Biol Chem *278*, 45288-45295.

Shen, X. (2004). Preparation and analysis of the INO80 complex. Methods Enzymol *377*, 401-412.

Vary, J.C., Jr., Fazzio, T.G., and Tsukiyama, T. (2004). Assembly of yeast chromatin using ISWI complexes. Methods Enzymol *375*, 88-102.
Wittmeyer, J., Saha, A., and Cairns, B. (2004). DNA translocation and nucleosome remodeling assays by the RSC chromatin remodeling complex. Methods Enzymol *377*, 322-343.
Yeeles, J.T., Deegan, T.D., Janska, A., Early, A., and Diffley, J.F. (2015). Regulated eukaryotic DNA replication origin firing with purified proteins. Nature *519*, 431-435.
