# Supplementary material for: Chromatin Controls DNA Replication Origin Selection, Lagging-Strand Synthesis, and Replication Fork Rates
Source: Mol Cell. 2017 Jan 5;65(1):117–30. doi: 10.1016/j.molcel.2016.11.016 (PMC5222724; doi:10.1016/j.molcel.2016.11.016)
Supplement: Document S2. Article plus Supplemental Information [file mmc3.pdf]

Molecular Cell

Article

Chromatin Controls DNA Replication Origin Selection, Lagging-Strand Synthesis, and Replication Fork Rates

Graphical Abstract

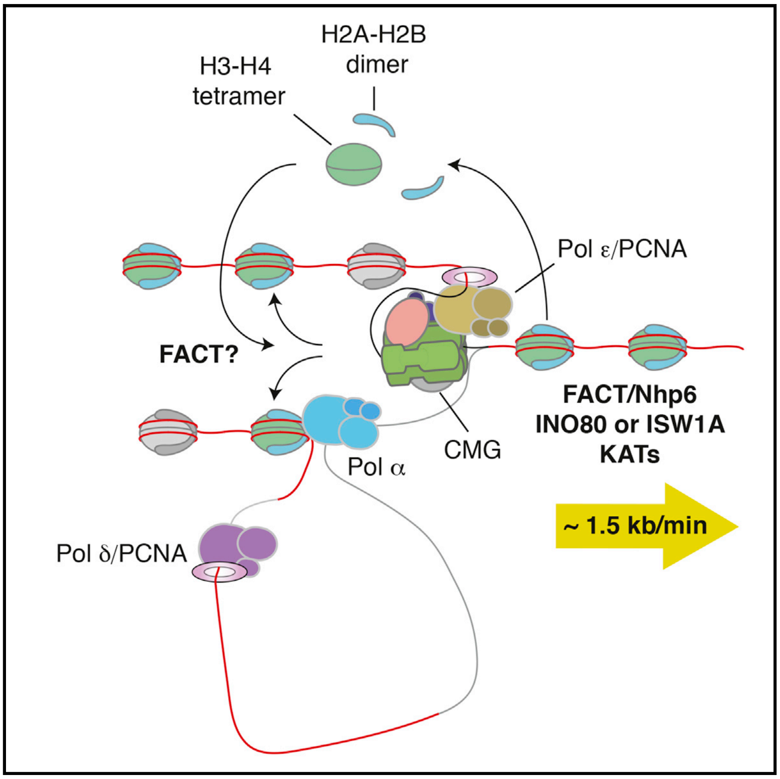

Highlights

- Reconstitution of eukaryotic chromatin replication with purified proteins
- Chromatin enforces origin-specific MCM loading
- FACT is essential for chromatin replication
- Nucleosomes are efficiently repositioned behind the replication fork

Authors

Christoph F. Kurat, Joseph T.P. Yeeles, Harshil Patel, Anne Early, John F.X. Diffley

Correspondence

john.diffley@crick.ac.uk

In Brief

By reconstituting chromatin replication with purified proteins, Kurat et al. show that timely progression of the replisome through chromatin requires a complex interplay between FACT, Nhp6, chromatin remodelers, and lysine acetyltransferases. Parental nucleosomes are efficiently re-assembled in the back of the replisome and positively influence lagging-strand synthesis.

Kurat et al., 2017, Molecular Cell *65*, 117–130
January 5, 2017 © 2017 The Authors. Published by Elsevier Inc.
http://dx.doi.org/10.1016/j.molcel.2016.11.016

Molecular Cell
Article

CellPress

Chromatin Controls DNA Replication Origin Selection, Lagging-Strand Synthesis, and Replication Fork Rates

Christoph F. Kurat,[1] Joseph T.P. Yeeles,[1,3] Harshil Patel,[2] Anne Early,[1] and John F.X. Diffley[1,4,*]
[1]Clare Hall Laboratory, Francis Crick Institute, South Mimms, Hertfordshire EN6 3LD, UK
[2]Lincoln's Inn Fields Laboratory, Francis Crick Institute, London NW1 1AT, UK
[3]Present address: Medical Research Council Laboratory of Molecular Biology, Cambridge CB2 OQH, UK
[4]Lead Contact
*Correspondence: john.diffley@crick.ac.uk
http://dx.doi.org/10.1016/j.molcel.2016.11.016

SUMMARY

The integrity of eukaryotic genomes requires rapid and regulated chromatin replication. How this is accomplished is still poorly understood. Using purified yeast replication proteins and fully chromatinized templates, we have reconstituted this process in vitro. We show that chromatin enforces DNA replication origin specificity by preventing non-specific MCM helicase loading. Helicase activation occurs efficiently in the context of chromatin, but subsequent replisome progression requires the histone chaperone FACT (facilitates chromatin transcription). The FACT-associated Nhp6 protein, the nucleosome remodelers INO80 or ISW1A, and the lysine acetyltransferases Gcn5 and Esa1 each contribute separately to maximum DNA synthesis rates. Chromatin promotes the regular priming of lagging-strand DNA synthesis by facilitating DNA polymerase α function at replication forks. Finally, nucleosomes disrupted during replication are efficiently re-assembled into regular arrays on nascent DNA. Our work defines the minimum requirements for chromatin replication in vitro and shows how multiple chromatin factors might modulate replication fork rates in vivo.

INTRODUCTION

Eukaryotic genomes are packaged into nucleosomes comprising 147 bp of duplex DNA wrapped around a histone octamer containing two copies each of the four core histones (H2A, H2B, H3, and H4) (Luger et al., 1997). Histones are highly basic proteins and nucleosomes are therefore very stable structures, requiring, for example, high salt concentrations for their removal from chromatin. Within this context, the replication machinery must define sites of replication initiation (origins), load the MCM replicative helicase, and activate it by converting it to the CMG (Cdc45-MCM-GINS) complex. Each and every nucleosome then must be transiently disrupted to allow duplex unwinding and DNA synthesis by the replisome. After passage of the replication forks, nucleosomes composed of histones from parental nucleosomes as well as newly synthesized histones must be rapidly re-assembled on both leading and lagging-strand replication products. Many "chromatin factors" have been described that affect chromatin structure or dynamics including histone chaperones, nucleosome remodelers, and enzymes that covalently modify histone subunits (Campos and Reinberg, 2009; Swygert and Peterson, 2014). The roles of these proteins in transcription, DNA repair, and DNA damage signaling have been well studied. Nonetheless, roles for these factors in chromatin replication are still poorly defined.

It may be that the eukaryotic replisome can replicate chromatin without additional factors, since a heterologous replisome from the bacteriophage T4 can replicate through nucleosomal DNA on its own (Bonne-Andrea et al., 1990). However, there is considerable evidence that additional chromatin factors play at least some part in this process (Alabert and Groth, 2012). Two histone chaperones have been implicated in eukaryotic DNA replication fork progression in vivo. FACT (facilitates chromatin transcription) is a strong candidate based on its physical association with both the CMG helicase and the lagging-strand DNA polymerase α (Pol α) (Gambus et al., 2006; Orphanides et al., 1998, 1999; Reinberg and Sims, 2006; Wittmeyer and Formosa, 1997). Consistent with this possibility, genes encoding both FACT subunits (Pob3 and Spt16) are essential for viability in yeast and a *pob3* hypomorphic mutant exhibits hydroxyurea sensitivity (Schlesinger and Formosa, 2000). FACT is essential for replication in *Xenopus* egg extracts (Okuhara et al., 1999) and deletion of the Pob3 ortholog in chicken DT40 cells causes a reduction in replication fork rates but not origin firing (Abe et al., 2011). Another histone chaperone, Asf1, interacts with MCM via histones H3-H4 in human cells, and depletion of Asf1 inhibits replisome progression during the S phase (Groth et al., 2007). Overexpression of histones H3-H4 has similar effects to Asf1 depletion, suggesting that Asf1 plays an important role in coordinating unwinding with histone dynamics at the fork. In contrast to genes encoding FACT, the ASF1 gene is not essential in yeast. In addition to FACT and Asf1, the N terminus of the Mcm2 subunit of the replicative CMG helicase has been shown to act as a histone H3-H4 chaperone (Foltman et al., 2013; Huang et al., 2015; Ishimi et al., 1998; Richet et al., 2015; Saade

CrossMark

This is an open access article under the CC BY license (http://creativecommons.org/licenses/by/4.0/).

et al., 2009). Similar to Asf1, mutation of this domain has relatively mild phenotypes in budding yeast (Foltman et al., 2013).

Roles for other chromatin factors in replication are less clear (Alabert and Groth, 2012). It may be that nucleosome remodelers and histone modifiers as well as the non-essential histone chaperones play little or no role in normal chromatin replication. Alternatively, they may be required for essential replication processes but may be highly redundant. These are difficult questions to address in vivo in part because of this potential redundancy and in part because chromatin factors including FACT also play key roles in gene expression, thus potentially affecting replication indirectly. Biochemical systems to address this in vitro have been lacking, so we set out to reconstitute this process with purified proteins.

RESULTS

Chromatin Enforces Origin Specificity

We assembled nucleosomes on plasmid DNA with recombinant yeast histones, the histone chaperone Nap1, and the nucleosome remodeler ISW1A (Figure S1A) as previously described (Vary et al., 2004). Because histones were expressed in *Escherichia coli* (Kingston et al., 2011), they should not harbor any covalent marks. Analysis of the nucleosome arrays produced by micrococcal nuclease (MNase) digestion showed a high density of evenly spaced nucleosomes in the population (Figure S1B). A similarly dense array was obtained with either linear or circular DNA attached to magnetic beads as well as circular plasmid DNA in solution (Figures S1B–S1D).

To characterize this further, we assembled chromatin on a 2.8-kb fragment of yeast DNA from the *TRP1-GAL3* locus with the ARS1 replication origin at its center, which was attached at one end to magnetic beads via a biotin-streptavidin linkage (Figure 1A; Figures S1A and S1B). We then digested the chromatinized templates to completion with MNase and deep-sequenced mononucleosomal DNA. Figure S2A presents normalized read numbers across the entire sequence, showing that phased nucleosomes can be found across the region. Despite the apparent clear phasing of nucleosomes in the bulk population (Figure S1), however, there are regions, for example, in the 3′ half of the *TRP1* gene, where phasing was less precise, as previously documented (Thoma et al., 1984). Consistent with previous work, there was a gap in the nucleosome map corresponding to the origin sequence, indicating that the origin was nucleosome free (Eaton et al., 2010). This gap was visible even in the absence of the origin recognition complex (ORC), but the presence of ORC during chromatin assembly further suppressed encroachment of nucleosomes into the origin (Figures S2A and S2B). The third panel of Figure S2B shows that this suppression of nucleosome encroachment occurred even when ORC was added after chromatin assembly. In all subsequent experiments, ORC was present during chromatin assembly.

The first step in DNA replication is the loading of the MCM helicase as a double hexamer bound around double-stranded DNA by ORC, Cdc6, and Cdt1 (Evrin et al., 2009; Remus et al., 2009). Figure 1B shows that, on both naked DNA and chromatin, MCM, along with the loading factors, was bound to DNA in ATP and ATPγS after a low salt wash. MCMs were assembled into a high-salt-resistant complex, which is a hallmark of loaded MCM double hexamers, in ATP but not ATPγS. ORC was reproducibly slightly more resistant to removal from chromatin with high salt, suggesting that it may interact with nucleosomes, consistent with previous work (Hizume et al., 2013). As is the case for MCM loading on naked DNA, this high-salt-resistant MCM complex on chromatin was dependent on both ORC and Cdc6 (Figure 1C). A high salt wash after chromatin assembly removed ISWA and Nap1 (Figures S2C and S2D). MCM loading, however, was not affected (Figure S2E), indicating that ISWA and Nap1 are not required for MCM loading on chromatin.

Previous work has shown that MCM loading on naked DNA does not exhibit strong origin dependence (Remus et al., 2009). Consistent with this, Figure 1D shows that MCM loading on naked DNA containing a wild-type origin or a mutant origin that lacks high-affinity ORC binding sites ($A^{-}B2^{-}$) (Bell and Stillman, 1992; Marahrens and Stillman, 1992; Remus et al., 2009) occurred equally efficiently at high ORC concentrations (lanes 5 and 6); dependence on the functional origin was only seen at relatively low ORC concentrations (e.g., lanes 1 and 2). On chromatin, MCM loading was efficient on the wild-type origin, but loading was greatly reduced on the mutant origin over a wide range of ORC concentrations. The lower panel of Figure 1D shows that these effects are reflected in ORC binding after a low salt wash: there was less ORC bound to chromatin, and specificity for the wild-type origin was maintained at all ORC concentrations tested. From these experiments, we conclude that chromatin enforces origin specificity by suppressing nonspecific ORC binding.

Chromatin Inhibits Replisome Progression

We next asked whether the loaded MCM complex could be efficiently converted to the CMG helicase in the context of chromatin. To do this, we assembled chromatin and loaded MCM as in the previous section, phosphorylated it with Dbf4-dependent kinase (DDK), and added the remainder of the required firing factors (Figure 2A) (Yeeles et al., 2015). The CMG is stable to high salt extraction, and Figure 2B shows that DDK-dependent CMG was formed on chromatin almost as efficiently as it was on naked DNA. However, DNA synthesis was strongly inhibited in the context of chromatin with either the minimal replisome (Yeeles et al., 2015; Figure 2C) or with the reconstituted replisome (without FACT) described in our accompanying manuscript in this issue of *Molecular Cell* (Yeeles et al., 2016) (Figure 2D). In all subsequent experiments with purified proteins, this reconstituted replisome was used. Thus, chromatin does not inhibit CMG assembly but it effectively prevents replisome progression, indicating that additional factors are required for chromatin replication.

To identify factors that might contribute to replisome progression through chromatin, we next asked whether an S phase extract (Gros et al., 2014; Heller et al., 2011; On et al., 2014) could replicate our chromatin template. As shown in Figure 2E, CMG assembly was supported on the chromatin template in extract, and this template replicated in a DDK-dependent manner almost as efficiently as naked DNA in these extracts (Figure 2F). Therefore, some factors in the extract must be acting with the replisome. To identify candidates, we performed quantitative,

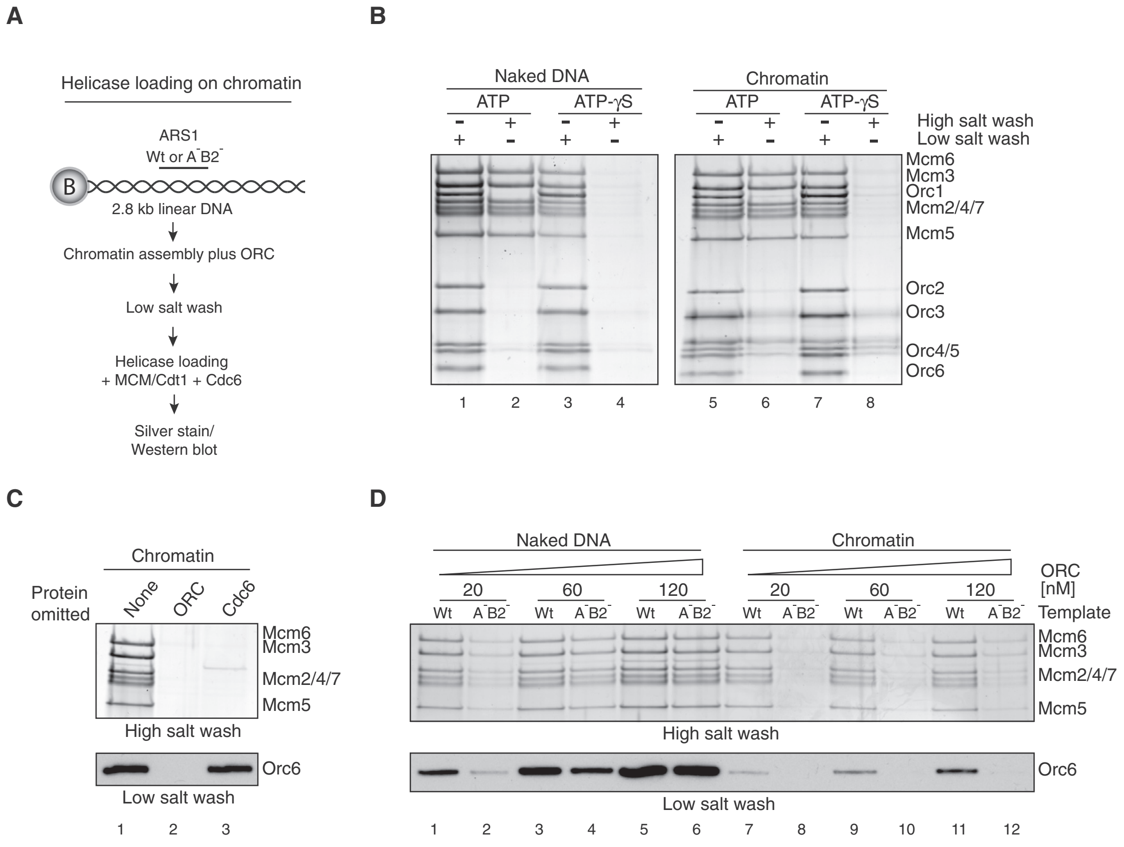

Figure 1. Loading of the MCM Complex on Chromatin

(A) Reaction scheme for chromatin assembly and MCM loading on ARS1-containing 2.8-kb linear DNA coupled to paramagnetic beads.

(B) Silver-stained gels of MCM loading reactions on naked DNA (left) compared to chromatin (right). In this and all subsequent experiments on bead-coupled DNA, ORC was added to the chromatin assembly reaction, followed by two 0.3-M K-acetate washes prior to MCM loading. Reactions were performed in the presence of 2 mM ATP or ATPγS. Loading reactions were washed either with 0.3 M K-acetate (low salt wash) or with 0.5 M NaCl (high salt wash).

(C) MCM loading in the presence and absence of ORC and Cdc6. Loading reactions were conducted as shown in (A). ORC binding was assessed after two low salt washes by immunoblotting using an antibody recognizing the Orc6 subunit.

(D) MCM loading and ORC binding on naked DNA and chromatin using wild-type (WT) and mutant ARS1 (A⁻B2⁻) origin DNA sequences. Reactions were performed as in (A) with indicated amounts of ORC.

See also Figures S1 and S2.

label-free mass spectrometry on the chromatin templates in the extracts. As described previously (On et al., 2014), we used intensity-based absolute quantification (IBAQ) (Schwanhäusser et al., 2011) to measure the relative abundance of proteins on assembled chromatin either with or without prior DDK treatment, and we plotted the $\log_{10}$ (IBAQ) scores (Figure S3). As expected, histones and MCM subunits were highly abundant and were found along the diagonal line, indicating that they were present at roughly equal levels with or without DDK. Focusing on chromatin factors, we found two lysine acetyltransferases enriched in the presence of DDK: Esa1, part of the NuA4 complex, and Gcn5, part of the SAGA complex. Although the enrichment of Gcn5 was modest, the enrichment of Esa1 was considerable (almost two orders of magnitude higher in the +DDK sample). The histone chaperones Asf1, FACT, and Nap1 were also identified. Finally, the remodelers INO80, RSC, and ISW1A were identified. Samples were not washed with high salt before they were added to the extract, so we cannot rule out that some ISW1A and/or Nap1 may have been carried over from the chromatin assembly step. None of these factors showed a significant enrichment in the +DDK sample, although most proteins were slightly above the diagonal. Nonetheless, these were the most abundant chromatin factors on our templates, so we investigated their roles in replication. The full list of proteins identified is provided in Table S3.

FACT Is Required for Chromatin Replication

To assess the roles of these proteins in replisome progression, we expressed and purified them all (Figure 3A; Figure S4). In addition to FACT and Asf1, we also expressed and purified Nhp6, a small HMG box-containing protein that is known to work with FACT (Brewster et al., 2001; Formosa et al., 2001; Stillman, 2010) and was identified by mass spectrometry (Figure S3). The complete INO80 complex was purified after overexpression in yeast; the endogenous RSC complex was also purified from yeast. The NuA4 and SAGA complexes each have large numbers

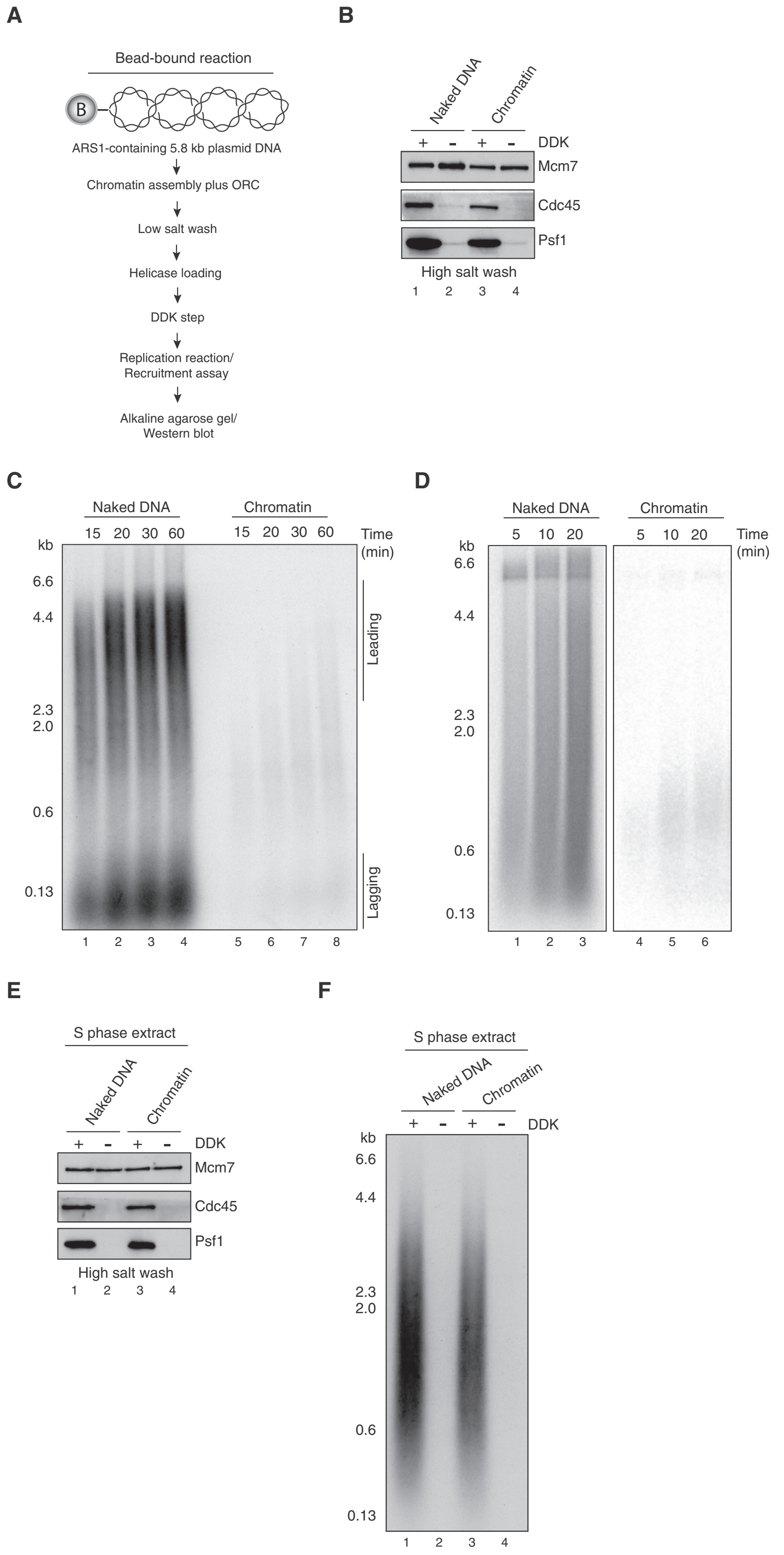

Figure 2. Chromatin Inhibits DNA Replication In Vitro

(A) Reaction scheme for replication reactions and CMG recruitment on 5.8-kb circular bead-bound ARS1-containing templates using S phase extract or purified proteins. Chromatin assembly and MCM loading were performed as in Figure 1A. Loaded MCMs were further phosphorylated with DDK before they were added to an S phase extract or purified replication proteins.

(B) CMG recruitment on naked DNA and on chromatin in the presence of purified initiation and replication factors (Sld3/7, Cdc45, Dpb11, Polε, GINS, Sld2, Mcm10, and S-CDK). Reactions were performed as in Figure 3A. Beads were collected and washed with 0.3 M KCl. Recruitment of the CMG with or without DDK was assessed by immunoblotting using antibodies recognizing Cdc45, Psf1 (GINS), and Mcm7 (MCM) subunits.

(C) Replication reactions on naked DNA and on chromatin conducted as shown in (A) using the minimal replication system (Yeeles et al., 2015). In this and all subsequent replication reactions, DNA was visualized by incorporation of [α^{32}P] deoxycytidine triphosphate (dCTP) into nascent DNA and products were separated through a 0.7% alkaline agarose gel.

(D) Replication reactions on naked DNA and on chromatin using the complete replication system as described in Yeeles et al., 2016.

(E) CMG recruitment in S phase extract on either naked DNA or chromatin in the presence and absence of DDK phosphorylation.

(F) Replication reactions on naked DNA compared to chromatin using S phase extract in the presence or absence of DDK.

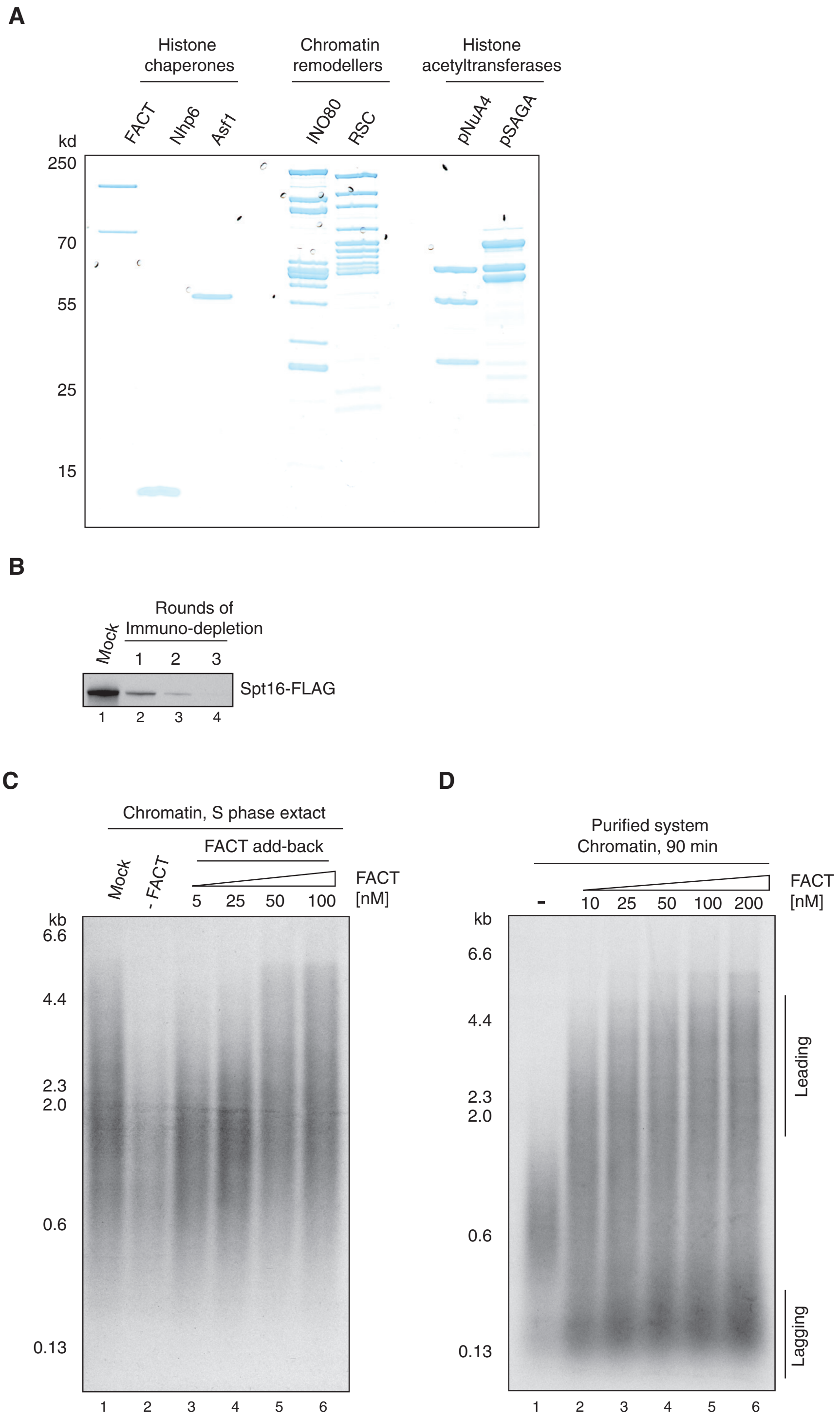

Figure 3. FACT Is Necessary and Sufficient for Chromatin Replication

(A) Purified histone chaperones, Nhp6 protein, chromatin remodelers, and histone acetyltransferases analyzed by SDS-PAGE with Coomassie staining.

(B) Immunodepletion of the Spt16 subunit of the FACT complex from an S phase extract (yCFK2) assessed by immunoblotting using an antibody recognizing the FLAG epitope.

(C) Dependence of chromatin replication on in S phase extract. Spt16 was immunodepleted as in (B) and replication reactions on chromatin were performed as described in (A). Indicated amounts of purified FACT were added back to the immunodepleted extract.

(D) Effect of FACT on chromatin replication using the complete replication system.

See also Figures S3–S5 and Table S3.

of subunits, but sub-assemblies have been identified that contain full acetyltransferase activity (pNuA4 and pSAGA) (Barrios et al., 2007). These were expressed and purified from *E. coli*.

In the accompanying manuscript (Yeeles et al., 2016), we showed that FACT has no effect on replication of naked DNA. To begin to examine the role of FACT in chromatin replication, we depleted it from our S phase extracts (Figure 3B). As shown in Figure 3C, FACT-depleted extracts were defective in replicating a chromatinized template, and addition of purified FACT restored replication activity of these extracts. Moreover, addition of FACT to the purified replication system increased the lengths of leading-strand replication products and the overall amount of lagging-strand products with purified proteins (Figure 3D). To rule out any contribution to replication from the ISW1A remodeler and the Nap1 chaperone used to assemble chromatin, we washed chromatin with high salt before replication, which removed ISW1A and Nap1 (Figures S2C and S2D). Figure S5 shows that replication of this high-salt-washed chromatin was stimulated by FACT to a similar extent as chromatin, which was not washed with high salt. From these experiments, we conclude that FACT is necessary and, to some extent, sufficient for chromatin replication.

Nhp6, INO80, ISW1A, and Histone Acetylation Stimulate Replication with FACT

We noticed that replication was slow, even with FACT. We considered that this may be due to interference caused by interactions between chromatin and the magnetic beads. We developed a protocol to assemble and purify chromatin on soluble 10.6-kb plasmids before replicating this in the soluble replication system described in the accompanying manuscript (Yeeles et al., 2016) (Figure 4A). Replication in this soluble system produced near full-length leading-strand replication

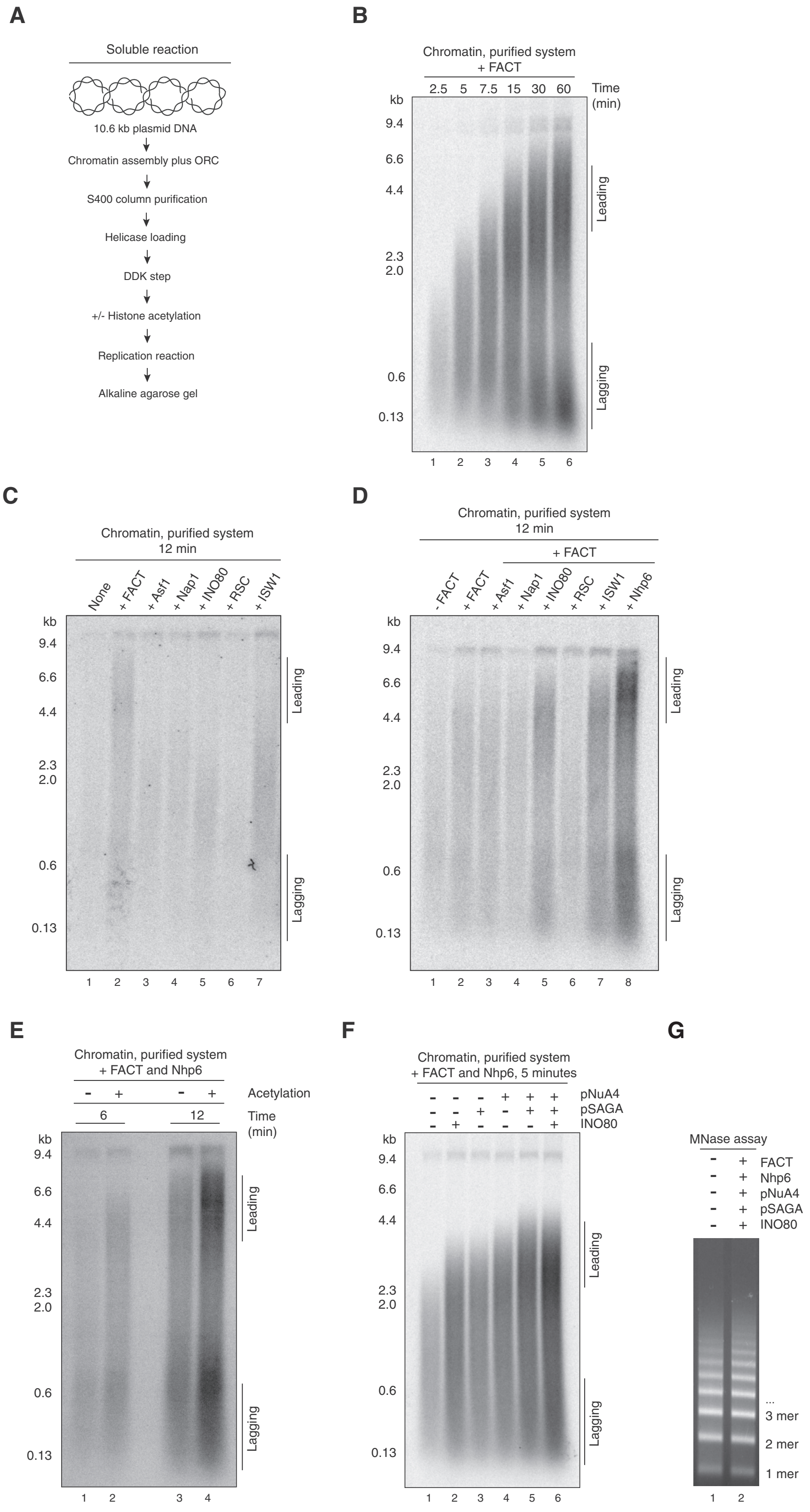

Figure 4. Contribution of Chromatin Remodelers and Lysine Acetyltransferases to Chromatin Replication

(A) Reaction scheme for soluble replication reaction on chromatin. Chromatin was assembled on ARS1-containing 10.6-kb plasmid DNA in solution in the presence of ORC. ORC-containing chromatinized circles were then isolated for subsequent steps by gel filtration. All subsequent replication reactions were performed using the complete replication system.

(B) Time course of a soluble chromatin replication reaction using the scheme shown in (A) in the presence of FACT added at the beginning of the reaction.

(C) Effect of histone chaperones and chromatin remodelers on chromatin replication assessed individually.

(D) Effect on chromatin replication of adding histone chaperones, chromatin remodelers, or Nhp6 together with FACT.

(E) Histone acetylation by catalytic subcomplexes of NuA4 (pNuA4) and SAGA (pSAGA) stimulate chromatin replication. Reactions were performed as described in (A). After DDK treatment, nucleosomes were acetylated using purified pNuA4, pSAGA, and acetyl-coenzyme A.

(F) Effect of adding of pNuA4, pSAGA, and INO80 together with FACT and Nhp6 on chromatin replication.

(G) Bulk chromatin with or without FACT, Nhp6, INO80, and histone acetylation was assessed by MNase digestion following native agarose gel electrophoresis and ethidium bromide staining.

See also Figures S6 and S7.

products; however, even in this soluble system, replication of chromatin with FACT alone was still relatively slow, not reaching completion until 30–60 min (Figure 4B).

We next looked at whether the other chromatin factors (Figure 3A) could stimulate replication on chromatin by looking at their effect on replication products at an early time point (12 min). Figure 4C shows that, while FACT could clearly stimulate replication at this time point, Asf1, Nap1, INO80, and RSC had no effect on replication. ISW1A had a very small effect on overall incorporation but did not significantly increase the length of the products, suggesting that it may weakly promote some step in initiation. We next asked whether any of these factors could stimulate replication with FACT, again at 12 min. Figure 4D shows that Asf1, Nap1, and RSC did not stimulate replication; in fact, RSC slightly but reproducibly inhibited synthesis. By contrast, the INO80 and ISW1A remodelers increased both the overall incorporation in the presence of FACT as well as the length of the leading-strand products, resulting in a clear distinction between leading and lagging-strand products even at this early time point. The biggest effect, however, was seen with Nhp6, which stimulated both overall incorporation and leading-strand length at this early time point more than either INO80 or ISW1A. Nhp6 had little or no effect on replication in the absence of FACT, nor did it stimulate replication with any of the other purified factors (Figure S6A), indicating that its effects are specific for FACT. In the presence of FACT and Nhp6, both ISW1A and INO80 further stimulated replication (Figure S6B).

We next investigated the effects of histone acetylation on chromatin replication. Figure S7A shows that the pNuA4 and pSAGA acetyltransferases are active and exhibit specific reactivity toward histone H4 and H3, respectively. Figure S7B shows that, in combination, they appeared to stimulate the rate of replication with FACT alone, and the combination of these acetyltransferases with FACT and Nhp6 greatly stimulated replication at early time points (Figure 4E) in an acetyl-coenzyme A-dependent manner (Figure S7C).

To assess the effects of combinations of factors, we examined replication at an even earlier time point (5 min). Figure 4F shows that, individually, INO80, pSAGA, and pNuA4 all stimulate replication with Nhp6 and FACT. Combining pSAGA and pNuA4 stimulates beyond the level of either individual acetyltransferase. The combination of INO80 with the two acetyltransferases stimulated incorporation at this early time point even further. Importantly, the full combination of FACT, Nhp6, INO80, pSAGA, and pNuA4 had no effect on the MNase digestion pattern (Figure 4G), indicating that the stimulation of replication was not due to non-specific removal of nucleosomes from the template. Multiple RSC subunits contain bromodomains, which bind acetylated histones, so we asked whether histone acetylation affected the ability of RSC to promote replication. We found that acetylation greatly stimulated the recruitment of RSC to chromatin (Figure S7D), but this recruited RSC did not stimulate replication. Instead, RSC more strongly inhibited replication after histone acetylation (Figure S7E).

Figure S7F shows that FACT was essential for chromatin replication even in the presence of the other chromatin factors, consistent with it being a key player in chromatin replication. The full complement of chromatin factors also greatly stimulated replication of a linear chromatin template (Figure S7G), indicating that the inhibition of replication is not simply due to some topological problems caused by chromatin. The presence of two discrete leading-strand products of 1.6 and 1.2 kb in this experiment also shows that most initiation occurs in or near the origin.

A time course with the full combination of positive-acting chromatin factors showed the appearance of full-length products within 10 min (Figure 5A), suggesting rapid replisome progression. As with the complete replisome on naked DNA (Yeeles et al., 2016), this replication resulted in equal amounts of leading and lagging-strand synthesis (Figure 5B). To quantify the fork rate, we performed a pulse chase experiment (Figure 5C). Analysis of this experiment (Figure 5D) indicates that leading-strand replication in this system proceeds at a maximum rate of approximately 1.4 kb/min, with bulk synthesis being 1.1 kb/min.

Chromatin Promotes Lagging-Strand Replication

In the accompanying manuscript (Yeeles et al., 2016), we show that, on a naked DNA template, the size of lagging-strand products is highly dependent on the concentration of Pol α across a wide range, indicating that Pol α acts distributively in this system. We noticed that lagging-strand products from chromatin templates, even with the full combination of factors, were considerably smaller and more discrete than those we routinely see on naked DNA. As shown in Figure 6A (lanes 1 and 3), lagging-strand products on naked DNA were considerably longer ($\sim$2 kb) at 5 nM Pol α than at 40 nM ($\sim$0.6 kb). By contrast, lagging-strand products from the chromatin template (lanes 2 and 4) were relatively short (0.15–0.5 kb) at both 5 and 40 nM Pol α. Further reduction of Pol α had little effect on lagging-strand length until 1 nM, at which point even leading-strand synthesis was reduced (Figure 6B). These experiments show that lagging-strand product length was relatively constant over a wide range of Pol α concentrations, indicating that Pol α no longer acts distributively in the context of chromatin. Because FACT interacts with both CMG and Pol α (Foltman et al., 2013; Wittmeyer and Formosa, 1997), we tested whether FACT alone could account for this effect. As shown in Figure 6C, the presence of FACT and/or Nhp6 did not lead to shorter leading-strand products on naked DNA at a low Pol α concentration, and Pol α still appeared to act distributively in lagging-strand synthesis across a range of Pol α concentrations (Figure 6D). Moreover, despite differential effects on overall synthesis, short lagging-strand products were seen when chromatin was used as a template in the absence of FACT and/or Nhp6 and any remodelers or acetyltransferases (Figure 6C, lanes 1–4). The presence of Ctf4 in the reaction when chromatin was used as the template made no difference in lagging-strand size at various Pol α concentrations (Figure S7H). Taken together, these experiments indicate that it is the presence of chromatin, rather than any of the chromatin factors, that functionally tethers Pol α to the replication fork.

Nucleosomes Are Re-deposited on Nascent DNA

Finally, we tested whether the parental nucleosomes assembled on our chromatin templates were transferred to nascent DNA by digesting the replicated products with MNase. Figures 7A and 7B show that replication products from naked DNA were rapidly digested to a small size by MNase, while MNase digestion of

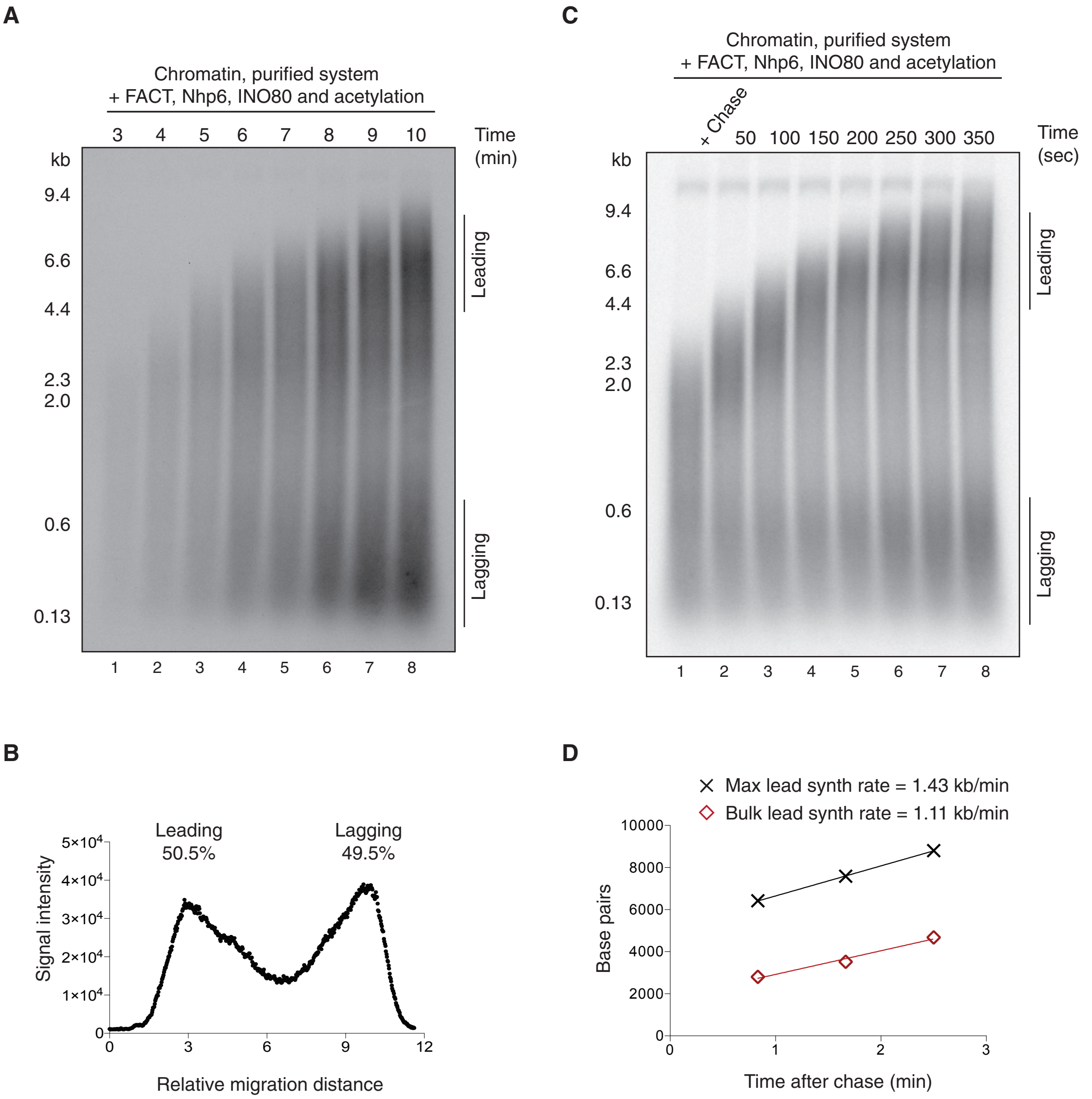

Figure 5. FACT, Nhp6, INO80, and Histone Acetylation Together Result in In Vivo Rates of Replisome Progression through Chromatin

(A) Time course of chromatin replication reactions as conducted in Figure 4A with FACT, Nhp6, INO80, and histone acetylation.

(B) Lane profile for the 10-min time point in Figure 6A.

(C) Pulse chase experiment to measure replication rates with the same reaction setup as in Figure 6A. For the pulse, the dCTP concentration was reduced to 4 mM. Following a 3-min incubation, unlabeled dCTP was added to a final concentration of 150 mM, and time points were taken every 50 s.

(D) Maximum (front) and peak product length for the experiment shown in (C). To derive maximum and bulk leading-strand synthesis rates, data were fitted to a linear regression.

replication products from chromatin revealed a characteristic ladder of repeating nucleosomes. Free histones were removed from our chromatin templates by gel filtration before they were added to the reactions, and the chromatin factors did not cause detectable displacement of histones from DNA (Figure 4G). Consequently, it is highly likely that nucleosomes assembled on nascent DNA derive from histones displaced from parental DNA during replication. To assess the efficiency of this nucleosome assembly, we quantified the amount of labeled product in the di- and tri-nucleosome bands in lane 4 of Figure 7B, relative to the total labeled product in lane 1, which showed that these nucleosomal bands accounted for approximately 34% of the total replication product. The mononucleosome band partially co-migrated with the digested free DNA, so this was not included in our analysis. If all of the parental nucleosomes displaced by the replisome were redeposited onto the nascent DNA, we would predict that the nucleosomal DNA would account for a maximum of 50% of the labeled product, because the DNA duplicates during replication. Thus, at least 68% (34 of 50) of the parental nucleosomes appear to be redeposited onto nascent DNA. This is likely to be an underestimate since it does not include the mononucleosome band.

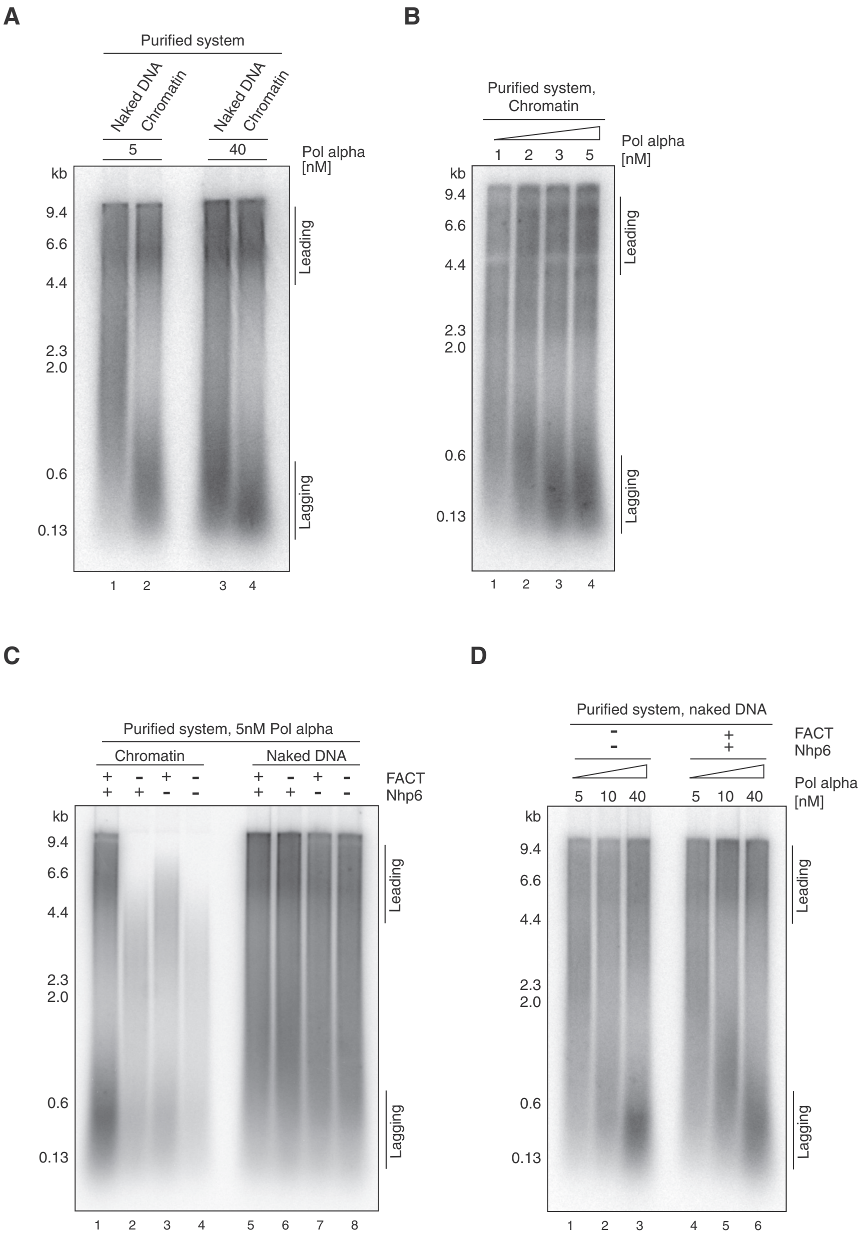

Figure 6. Chromatin Influences Lagging-Strand Size

(A) Replication reactions on naked DNA and chromatin were conducted as in Figure 4A with the amounts of Pol α as indicated. For chromatin replication, nucleosomes were acetylated and FACT, Nhp6, and INO80 were added.

(B) Replication reactions on chromatin as in (A) with indicated amounts of Pol α.

(C) Replication reactions on naked DNA versus chromatin in the presence or absence of FACT and Nhp6. Nucleosomes were not acetylated and INO80 was omitted.

(D) Replication reactions on naked DNA as in (A) in the presence or absence of FACT and Nhp6. Pol α was added at the indicated amounts.

See also Figure S7.

DISCUSSION

We have described the reconstitution of DNA replication on chromatin templates. Fork rates measured in this system fall well within the range of fork rates measured in vivo. Results described here have allowed us to identify three crucial roles for chromatin in replication: origin selection, fork rate modulation, and priming of lagging-strand synthesis.

Origin Selection

Previous work showed that replication origins have an inherent tendency to exclude nucleosomes even in the absence of ORC (Eaton et al., 2010). ORC reinforces this nucleosome free region (NFR), probably because its tight binding to specific DNA sequences acts as a barrier to new nucleosome assembly or sliding of adjacent nucleosomes into the NFR (Figure 7C, i). We have extended these findings by showing that chromatin also enforces origin specificity by suppressing non-specific ORC binding. ORC binding to non-specific DNA has a high off rate and we propose that as a consequence (Figure 7C, ii), nucleosomes act as a barrier to ORC binding. Metazoan ORC is a non-specific DNA binding protein (Vashee et al., 2003). We suggest that the suppression of ORC binding by chromatin described here may help restrict ORC to NFRs, a feature seen in mammalian replication origins in vivo (Cayrou et al., 2011; Lubelsky et al., 2011; MacAlpine et al., 2010).

FACT and Replisome Progression through Chromatin

The most important chromatin factor required for replisome progression through nucleosomes is the histone chaperone FACT: although FACT does not affect replication of naked DNA (Yeeles et al., 2016; Figure 6C), loss of FACT severely inhibited chromatin replication in extracts (Figure 3C) and in the fully reconstituted system (Figure S7F), and addition of FACT to the purified replication system was sufficient for complete but slow replication (Figure 3D). None of the other chaperones, remodelers, or lysine acetyltransferases tested could substitute for FACT in chromatin replication (Figure 4C; Figure S7F).

Because FACT has no effect on bulk nucleosome occupancy or spacing, even with other chromatin factors (Figure 4G), and because FACT interacts with multiple replisome components and functions in chromatin replication at concentrations similar to those of replication factors rather than histones, we favor the idea that FACT acts with the replisome to promote replication

CellPress

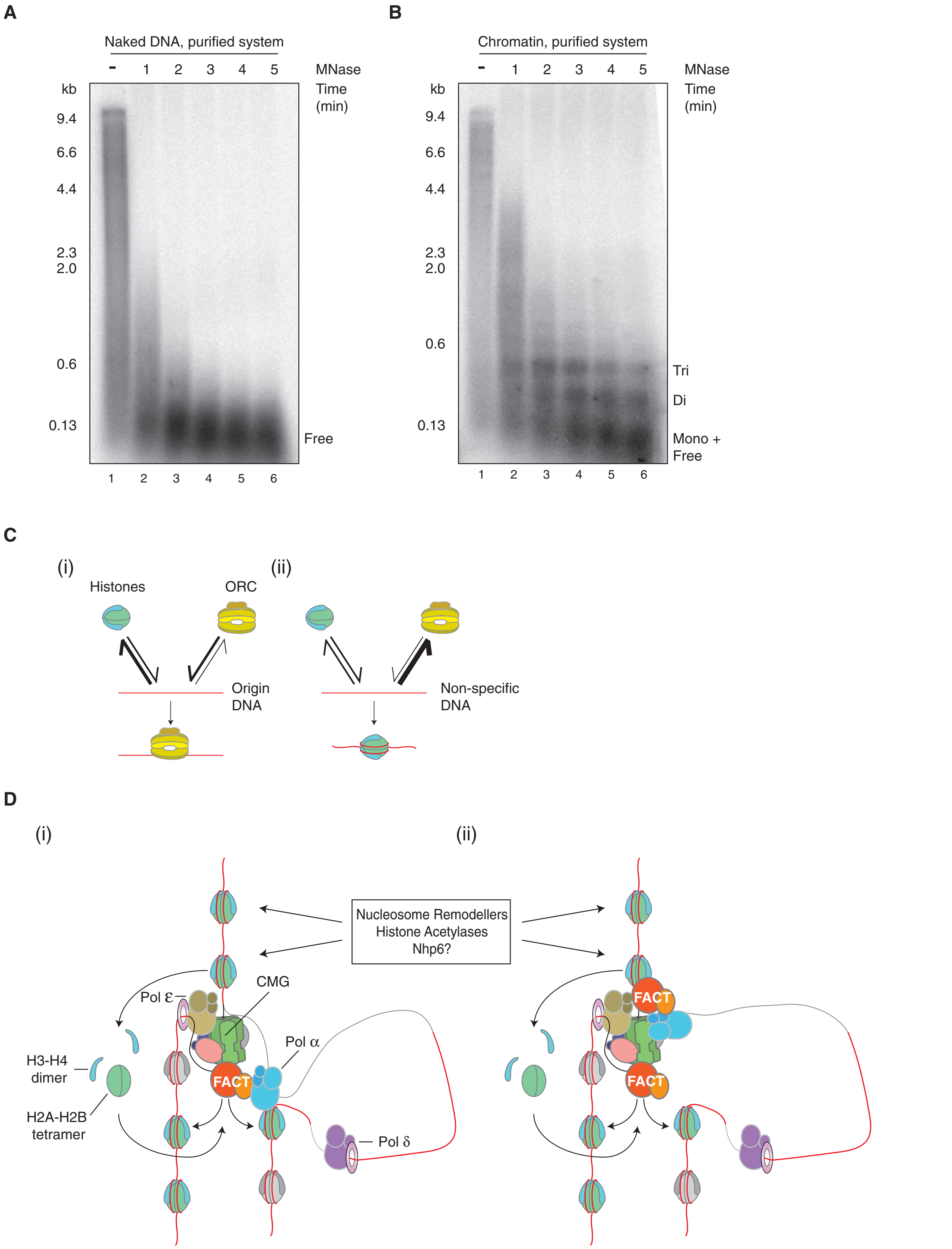

(legend on next page)

through chromatin. FACT may act by displacing nucleosomes ahead of the fork, and/or by re-depositing nucleosomes behind the fork. In transcription, FACT destabilizes nucleosomes ahead of RNA polymerase (Hsieh et al., 2013; Orphanides et al., 1998) but also prevents release of histones and promotes rapid re-establishment of chromatin after transcription (Hainer et al., 2012; Jamai et al., 2009). Recent evidence in yeast showing genetic and physical interactions between FACT and replication-coupled nucleosome assembly (Yang et al., 2016) suggests that FACT may also promote re-establishment of chromatin after replication. This would be consistent with the very efficient re-deposition of histones from parental nucleosomes onto nascent DNA (Figures 7A and 7B). We suggest two models (Figure 7D) for how nucleosomes ahead of the replication fork are displaced. First, disruption of nucleosomes ahead of the fork may not require FACT but may simply be a consequence of DNA unwinding by CMG, similar to the way the T4 replisome can displace nucleosomes (Figure 7D, i) (Bonne-Andrea et al., 1990). In this model, FACT's crucial role is in "accepting" these displaced histones, transferring them behind the fork into nucleosomes on the leading and lagging strands. In the absence of FACT, these displaced histones somehow interfere with the replisome, preventing normal progression. A second model (Figure 7D, ii) envisions a role for FACT in disrupting nucleosomes ahead of the fork. In this model, interaction of FACT with the CMG helicase (Foltman et al., 2013), Pol α (Wittmeyer and Formosa, 1997), or some other replisome component positions FACT to act ahead of the replication fork, where it contributes to displacing parental nucleosomes.

Lagging-Strand Synthesis

A functional interaction between FACT, Pol α, and nucleosomes is also attractive because of the effect that chromatin has on lagging-strand synthesis. As discussed in the accompanying manuscript (Yeeles et al., 2016), Pol α, despite its interaction with Ctf4 and Mcm10, is not functionally tethered to the replisome; but on chromatin, lagging-strand product lengths are short and relatively constant over a wide range of Pol α concentrations. It may be that interactions between Pol α, FACT, and nucleosomes, either behind (Figure 7D, i) or ahead of (Figure 7D, ii) the fork, physically tether Pol α to the fork. Alternatively, chromatin may not physically tether Pol α to the replication fork; rather, pausing of the replisome at each nucleosome ahead of the fork may promote a priming event behind the fork, perhaps via some structural change in the replisome that promotes transient recruitment of Pol α.

Chromatin assembly behind the replication fork is thought to dictate the positioning of lagging-strand product junctions by restricting strand displacement synthesis by Pol δ (Smith and Whitehouse, 2012). We do not have a flap endonuclease like Fen1 or Dna2 in our reactions, so the lengths of our products reflect the effect of chromatin on synthesis, not downstream cleavage. Our results, together with those of Smith and Whitehouse (2012), suggest that chromatin regulates the length of lagging-strand products by affecting both the rate of priming and the position of flap cleavage.

The regeneration of chromatin after DNA replication in vivo involves both re-deposition of parental histones into nucleosomes and assembly of newly synthesized histones into nucleosomes. Chromatin assembly factor 1 (CAF-1), the key histone chaperone in the de novo pathway (Smith and Stillman, 1989), is not required for the efficient re-deposition of parental histones onto nascent DNA as we have described here, suggesting that these are truly two distinct pathways for nucleosome assembly on nascent DNA. It will be important to determine how these processes are coordinated. The products after MNase digestion of nascent DNA were primarily mono-, di-, and tri-nucleosomes (Figure 7B), whereas the starting templates had more extensive nucleosome arrays (Figures S1B–S1D). Presumably, the de novo pathway will be required to regenerate the full nucleosome array.

Modulation of Replication Fork Rate by Chromatin Factors

Our data show that Nhp6, along with nucleosome remodelers and lysine acetyltransferases, acts additively to modulate the replication fork rate. INO80 has a role in removing nucleosomes from around double-strand DNA breaks (Li and Tyler, 2016; Tsukuda et al., 2005) and is also involved in resolving replication-transcription conflicts (Poli et al., 2016). Loss of both INO80 and chromatin accessibility complex (CHRAC) remodelers in vivo causes reduced nucleosome accessibility to MNase around replication forks in methyl methanesulfonate (MMS) (Lee et al., 2015), consistent with the idea that these remodelers play some role in disrupting nucleosomes during replication through damaged DNA. Our results suggest that INO80 may also play a role in normal replication. The catalytic subunit of CHRAC is the Isw1 paralog Isw2. Moreover, CHRAC shares a subunit, Dpb4, with Pol ε (Iida and Araki, 2004), suggesting that CHRAC, like ISW1A, may also be able to accelerate the fork rate. It is likely that other nucleosome remodelers and histone modifiers are also able to contribute to replisome progression. We note that RSC is recruited more efficiently to acetylated nucleosomes, consistent with the presence of bromodomain-containing subunits (Figure S7E). We speculate that some remodelers may have effects in specific regions of the genome enriched in particular histone marks, as the ACF1-ISWI complex has a role in heterochromatin replication in human cells (Collins et al., 2002). We also note that, even with histone acetylation, RSC does not stimulate replisome progression (Figure S7D); indeed, acetylation

Figure 7. Nucleosomes Are Re-deposited on Nascent DNA

(A and B) Nucleosomes are re-deposited on nascent DNA. MNase digestion of replicated products of naked DNA compared to those of chromatin. Replication products were treated with 100 U MNase and samples were taken every minute, quenched with EGTA, and analyzed on a 1.3% alkaline agarose gel. Replication products were visualized by autoradiography.

(C) Model of how chromatin influences origin selection. See the Discussion for details.

(D) Model of FACT-dependent replisome progression through chromatin. Parental nucleosomes are in green/light blue. Nucleosomes including newly synthesized histones are in gray/light gray. Double-stranded DNA is in red and single-stranded DNA is in gray. See the Discussion for details.

promoted even further inhibition of replication by RSC, indicating that not all remodelers can promote replication. We do not currently know whether this inhibition of replication by RSC plays any role in regulating fork rate in vivo.

Whether any of the chromatin factors we have examined other than FACT are specifically targeted to replication forks is unknown. In contrast to FACT, Nhp6 is required at much higher levels than replication factors to exert its effect on replication, suggesting that it may act distributively on chromatin. That INO80 and CHRAC contribute to changes in nucleosome accessibility specifically around replication forks suggests that they may be targeted to replication forks (Lee et al., 2015). Recent work has shown that INO80 is recruited to replication forks in human cells through interaction with ubiquitylated H2A aided by BRCA1-associated protein-1 (BAP1) (Lee et al., 2014), but it is unclear whether or how INO80 is being recruited to forks in our system. We found that recruitment of Esa1 to chromatin was enhanced by DDK, suggesting some coupling of it with the replication fork (Figure S3). Both Gcn5 and Rtt109 can acetylate histone H3K9, a mark that was recently shown to precede passage of the replication fork (Bar-Ziv et al., 2016). It was shown that the H3K9Ac, which precedes the fork, requires Rtt109, while the H3K9Ac at promoters requires Gcn5, so it may be that Rtt109 is the more relevant acetyltransferase for bulk replisome progression. Nonetheless, Gcn5 can clearly contribute to replisome progression, at least in vitro, and may aid replisome progression in specific regions like promoters in vivo.

Based on our work, we propose that replication fork rates in vivo reflect a complex interplay between different chromatin factors and histone modifications. Replication fork speed in vivo can be significantly affected by expression of a number of oncogenes during oncogene-induced replicative stress. While it is often assumed that this reflects some misregulation of the DNA replication machinery, we suggest that it may in some cases reflect changes in the availability and distribution of nucleosome remodelers and various histone marks. Some oncogenes, like c-Myc, are known to dramatically affect gene expression patterns genomewide (Zeller et al., 2006), and this may indirectly affect fork rates through chromatin factors.

The reconstitution of efficient replication through chromatin with purified proteins represents a major advance in understanding how chromosomes are duplicated and will provide novel approaches to understand how marked nucleosomes and gene expression patterns are inherited during DNA replication.

EXPERIMENTAL PROCEDURES

Yeast Strains and Proteins

Detailed information about yeast strain construction and protein purifications can be found in the Supplemental Experimental Procedures.

Chromatin Assembly and MCM Loading

Chromatin was assembled on different DNA templates as described previously (Vary et al., 2004). MCM loading on chromatin was conducted as described in the Supplemental Experimental Procedures.

Nucleosome Positioning

Chromatin was assembled on 2.8-kb linear DNA and mononucleosomal DNA was generated by MNase digestion. Mononucleosomal DNA was sequenced as described in the Supplemental Experimental Procedures.

S Phase Extracts and Mass Spectrometry Analyses

S phase extracts were prepared as described previously (On et al., 2014) and in the Supplemental Experimental Procedures. Chromatin was assembled and MCMs were loaded as described. Samples were treated with 100 nM DDK for 30 min at 30°C. S phase extract was added and incubated for another 30 min. After two washing steps (45 mM HEPES-KOH [pH 7.6], 5 mM Mg(OAc)2, 0.02% NP-40, 10% glycerol, and 0.3 M KOAc) and MNase treatment to digest DNA, chromatin-associated proteins were analyzed by mass spectrometry as described previously (On et al., 2014).

CMG Recruitment and Replication Reactions

CMG recruitment was performed on ARS1-containing bead-coupled plasmid DNA. Replication reactions were performed on both bead-coupled and soluble plasmid DNA. Detailed information can be found in the Supplemental Experimental Procedures.

SUPPLEMENTAL INFORMATION

Supplemental Information includes Supplemental Experimental Procedures, seven figures, and three tables and can be found with this article online at http://dx.doi.org/10.1016/j.molcel.2016.11.016.

AUTHOR CONTRIBUTIONS

C.F.K. performed all the experiments. J.T.P.Y. provided some proteins and helped with data analyses. H.P. analyzed nucleosome positioning data. A.E. helped with design and construction of the INO80 overexpression strain. C.F.K. and J.F.X.D. designed the experiments and wrote the paper.

ACKNOWLEDGMENTS

We thank T. Formosa, R. Kornberg, S. Tan, M. Webster, and M. Singleton for plasmids, strains, and helpful advice. We also thank J. Hill for Asf1 protein, K. Labib for Psf1 antibody, A. Alidoust and N. Patel for yeast and bacterial culture growth, and B. Snijders and D. Frith for mass spectrometry analyses. This work was supported by the Francis Crick Institute, which receives its core funding from Cancer Research UK (FC001066), the UK Medical Research Council (FC001066), and the Wellcome Trust (FC001066). This work was also funded by a Wellcome Trust Senior Investigator Award (106252/Z/14/Z) and a European Research Council Advanced Grant (669424-CHROMOREP) to J.F.X.D. C.F.K. was supported by a European Commission Marie Curie fellowship (FP7-PEOPLE-2012-IEF), and J.T.P.Y. was supported by a Federation of European Biochemical Societies Return-to-Europe fellowship.

Received: July 18, 2016
Revised: October 17, 2016
Accepted: November 7, 2016
Published: December 15, 2016

REFERENCES

Abe, T., Sugimura, K., Hosono, Y., Takami, Y., Akita, M., Yoshimura, A., Tada, S., Nakayama, T., Murofushi, H., Okumura, K., et al. (2011). The histone chaperone facilitates chromatin transcription (FACT) protein maintains normal replication fork rates. J. Biol. Chem. *286*, 30504–30512.

Alabert, C., and Groth, A. (2012). Chromatin replication and epigenome maintenance. Nat. Rev. Mol. Cell Biol. *13*, 153–167.

Bar-Ziv, R., Voichek, Y., and Barkai, N. (2016). Chromatin dynamics during DNA replication. Genome Res. *26*, 1245–1256.

Barrios, A., Selleck, W., Hnatkovich, B., Kramer, R., Sermwittayawong, D., and Tan, S. (2007). Expression and purification of recombinant yeast Ada2/Ada3/Gcn5 and Piccolo NuA4 histone acetyltransferase complexes. Methods *41*, 271–277.

Bell, S.P., and Stillman, B. (1992). ATP-dependent recognition of eukaryotic origins of DNA replication by a multiprotein complex. Nature *357*, 128–134.

Bonne-Andrea, C., Wong, M.L., and Alberts, B.M. (1990). In vitro replication through nucleosomes without histone displacement. Nature *343*, 719–726.

Brewster, N.K., Johnston, G.C., and Singer, R.A. (2001). A bipartite yeast SSRP1 analog comprised of Pob3 and Nhp6 proteins modulates transcription. Mol. Cell. Biol. *21*, 3491–3502.

Campos, E.I., and Reinberg, D. (2009). Histones: annotating chromatin. Annu. Rev. Genet. *43*, 559–599.

Cayrou, C., Coulombe, P., Vigneron, A., Stanojcic, S., Ganier, O., Peiffer, I., Rivals, E., Puy, A., Laurent-Chabalier, S., Desprat, R., and Méchali, M. (2011). Genome-scale analysis of metazoan replication origins reveals their organization in specific but flexible sites defined by conserved features. Genome Res. *21*, 1438–1449.

Collins, N., Poot, R.A., Kukimoto, I., García-Jiménez, C., Dellaire, G., and Varga-Weisz, P.D. (2002). An ACF1-ISWI chromatin-remodeling complex is required for DNA replication through heterochromatin. Nat. Genet. *32*, 627–632.

Eaton, M.L., Galani, K., Kang, S., Bell, S.P., and MacAlpine, D.M. (2010). Conserved nucleosome positioning defines replication origins. Genes Dev. *24*, 748–753.

Evrin, C., Clarke, P., Zech, J., Lurz, R., Sun, J., Uhle, S., Li, H., Stillman, B., and Speck, C. (2009). A double-hexameric MCM2-7 complex is loaded onto origin DNA during licensing of eukaryotic DNA replication. Proc. Natl. Acad. Sci. USA *106*, 20240–20245.

Foltman, M., Evrin, C., De Piccoli, G., Jones, R.C., Edmondson, R.D., Katou, Y., Nakato, R., Shirahige, K., and Labib, K. (2013). Eukaryotic replisome components cooperate to process histones during chromosome replication. Cell Rep. *3*, 892–904.

Formosa, T., Eriksson, P., Wittmeyer, J., Ginn, J., Yu, Y., and Stillman, D.J. (2001). Spt16-Pob3 and the HMG protein Nhp6 combine to form the nucleosome-binding factor SPN. EMBO J. *20*, 3506–3517.

Gambus, A., Jones, R.C., Sanchez-Diaz, A., Kanemaki, M., van Deursen, F., Edmondson, R.D., and Labib, K. (2006). GINS maintains association of Cdc45 with MCM in replisome progression complexes at eukaryotic DNA replication forks. Nat. Cell Biol. *8*, 358–366.

Gros, J., Devbhandari, S., and Remus, D. (2014). Origin plasticity during budding yeast DNA replication in vitro. EMBO J. *33*, 621–636.

Groth, A., Corpet, A., Cook, A.J., Roche, D., Bartek, J., Lukas, J., and Almouzni, G. (2007). Regulation of replication fork progression through histone supply and demand. Science *318*, 1928–1931.

Hainer, S.J., Charsar, B.A., Cohen, S.B., and Martens, J.A. (2012). Identification of mutant versions of the Spt16 histone chaperone that are defective for transcription-coupled nucleosome occupancy in Saccharomyces cerevisiae. G3 (Bethesda) *2*, 555–567.

Heller, R.C., Kang, S., Lam, W.M., Chen, S., Chan, C.S., and Bell, S.P. (2011). Eukaryotic origin-dependent DNA replication in vitro reveals sequential action of DDK and S-CDK kinases. Cell *146*, 80–91.

Hizume, K., Yagura, M., and Araki, H. (2013). Concerted interaction between origin recognition complex (ORC), nucleosomes and replication origin DNA ensures stable ORC-origin binding. Genes Cells *18*, 764–779.

Hsieh, F.K., Kulaeva, O.I., Patel, S.S., Dyer, P.N., Luger, K., Reinberg, D., and Studitsky, V.M. (2013). Histone chaperone FACT action during transcription through chromatin by RNA polymerase II. Proc. Natl. Acad. Sci. USA *110*, 7654–7659.

Huang, H., Strømme, C.B., Saredi, G., Hödl, M., Strandsby, A., González-Aguilera, C., Chen, S., Groth, A., and Patel, D.J. (2015). A unique binding mode enables MCM2 to chaperone histones H3-H4 at replication forks. Nat. Struct. Mol. Biol. *22*, 618–626.

Iida, T., and Araki, H. (2004). Noncompetitive counteractions of DNA polymerase epsilon and ISW2/yCHRAC for epigenetic inheritance of telomere position effect in Saccharomyces cerevisiae. Mol. Cell. Biol. *24*, 217–227.

Ishimi, Y., Komamura, Y., You, Z., and Kimura, H. (1998). Biochemical function of mouse minichromosome maintenance 2 protein. J. Biol. Chem. *273*, 8369–8375.

Jamai, A., Puglisi, A., and Strubin, M. (2009). Histone chaperone Spt16 promotes redeposition of the original H3-H4 histones evicted by elongating RNA polymerase. Mol. Cell *35*, 377–383.

Kingston, I.J., Yung, J.S., and Singleton, M.R. (2011). Biophysical characterization of the centromere-specific nucleosome from budding yeast. J. Biol. Chem. *286*, 4021–4026.

Lee, H.S., Lee, S.A., Hur, S.K., Seo, J.W., and Kwon, J. (2014). Stabilization and targeting of INO80 to replication forks by BAP1 during normal DNA synthesis. Nat. Commun. *5*, 5128.

Lee, L., Rodriguez, J., and Tsukiyama, T. (2015). Chromatin remodeling factors Isw2 and Ino80 regulate checkpoint activity and chromatin structure in S phase. Genetics *199*, 1077–1091.

Li, X., and Tyler, J.K. (2016). Nucleosome disassembly during human non-homologous end joining followed by concerted HIRA- and CAF-1-dependent reassembly. eLife *5*, e15129.

Lubelsky, Y., Sasaki, T., Kuipers, M.A., Lucas, I., Le Beau, M.M., Carignon, S., Debatisse, M., Prinz, J.A., Dennis, J.H., and Gilbert, D.M. (2011). Pre-replication complex proteins assemble at regions of low nucleosome occupancy within the Chinese hamster dihydrofolate reductase initiation zone. Nucleic Acids Res. *39*, 3141–3155.

Luger, K., Mäder, A.W., Richmond, R.K., Sargent, D.F., and Richmond, T.J. (1997). Crystal structure of the nucleosome core particle at 2.8 A resolution. Nature *389*, 251–260.

MacAlpine, H.K., Gordân, R., Powell, S.K., Hartemink, A.J., and MacAlpine, D.M. (2010). Drosophila ORC localizes to open chromatin and marks sites of cohesin complex loading. Genome Res. *20*, 201–211.

Marahrens, Y., and Stillman, B. (1992). A yeast chromosomal origin of DNA replication defined by multiple functional elements. Science *255*, 817–823.

Okuhara, K., Ohta, K., Seo, H., Shioda, M., Yamada, T., Tanaka, Y., Dohmae, N., Seyama, Y., Shibata, T., and Murofushi, H. (1999). A DNA unwinding factor involved in DNA replication in cell-free extracts of Xenopus eggs. Curr. Biol. *9*, 341–350.

On, K.F., Beuron, F., Frith, D., Snijders, A.P., Morris, E.P., and Diffley, J.F.X. (2014). Prereplicative complexes assembled in vitro support origin-dependent and independent DNA replication. EMBO J. *33*, 605–620.

Orphanides, G., LeRoy, G., Chang, C.H., Luse, D.S., and Reinberg, D. (1998). FACT, a factor that facilitates transcript elongation through nucleosomes. Cell *92*, 105–116.

Orphanides, G., Wu, W.H., Lane, W.S., Hampsey, M., and Reinberg, D. (1999). The chromatin-specific transcription elongation factor FACT comprises human SPT16 and SSRP1 proteins. Nature *400*, 284–288.

Poli, J., Gerhold, C.B., Tosi, A., Hustedt, N., Seeber, A., Sack, R., Herzog, F., Pasero, P., Shimada, K., Hopfner, K.P., and Gasser, S.M. (2016). Mec1, INO80, and the PAF1 complex cooperate to limit transcription replication conflicts through RNAPII removal during replication stress. Genes Dev. *30*, 337–354.

Reinberg, D., and Sims, R.J., 3rd (2006). de FACTo nucleosome dynamics. J. Biol. Chem. *281*, 23297–23301.

Remus, D., Beuron, F., Tolun, G., Griffith, J.D., Morris, E.P., and Diffley, J.F.X. (2009). Concerted loading of Mcm2-7 double hexamers around DNA during DNA replication origin licensing. Cell *139*, 719–730.

Richet, N., Liu, D., Legrand, P., Velours, C., Corpet, A., Gaubert, A., Bakail, M., Moal-Raisin, G., Guerois, R., Compper, C., et al. (2015). Structural insight into how the human helicase subunit MCM2 may act as a histone chaperone together with ASF1 at the replication fork. Nucleic Acids Res. *43*, 1905–1917.

Saade, E., Mechold, U., Kulyyassov, A., Vertut, D., Lipinski, M., and Ogryzko, V. (2009). Analysis of interaction partners of H4 histone by a new proteomics approach. Proteomics *9*, 4934–4943.

Schlesinger, M.B., and Formosa, T. (2000). POB3 is required for both transcription and replication in the yeast Saccharomyces cerevisiae. Genetics *155*, 1593–1606.

Schwanhäusser, B., Busse, D., Li, N., Dittmar, G., Schuchhardt, J., Wolf, J., Chen, W., and Selbach, M. (2011). Global quantification of mammalian gene expression control. Nature *473*, 337–342.

Smith, S., and Stillman, B. (1989). Purification and characterization of CAF-I, a human cell factor required for chromatin assembly during DNA replication in vitro. Cell *58*, 15–25.

Smith, D.J., and Whitehouse, I. (2012). Intrinsic coupling of lagging-strand synthesis to chromatin assembly. Nature *483*, 434–438.

Stillman, D.J. (2010). Nhp6: a small but powerful effector of chromatin structure in Saccharomyces cerevisiae. Biochim. Biophys. Acta *1799*, 175–180.

Swygert, S.G., and Peterson, C.L. (2014). Chromatin dynamics: interplay between remodeling enzymes and histone modifications. Biochim. Biophys. Acta *1839*, 728–736.

Thoma, F., Bergman, L.W., and Simpson, R.T. (1984). Nuclease digestion of circular TRP1ARS1 chromatin reveals positioned nucleosomes separated by nuclease-sensitive regions. J. Mol. Biol. *177*, 715–733.

Tsukuda, T., Fleming, A.B., Nickoloff, J.A., and Osley, M.A. (2005). Chromatin remodelling at a DNA double-strand break site in Saccharomyces cerevisiae. Nature *438*, 379–383.

Vary, J.C., Jr., Fazzio, T.G., and Tsukiyama, T. (2004). Assembly of yeast chromatin using ISWI complexes. Methods Enzymol. *375*, 88–102.

Vashee, S., Cvetic, C., Lu, W., Simancek, P., Kelly, T.J., and Walter, J.C. (2003). Sequence-independent DNA binding and replication initiation by the human origin recognition complex. Genes Dev. *17*, 1894–1908.

Wittmeyer, J., and Formosa, T. (1997). The Saccharomyces cerevisiae DNA polymerase alpha catalytic subunit interacts with Cdc68/Spt16 and with Pob3, a protein similar to an HMG1-like protein. Mol. Cell. Biol. *17*, 4178–4190.

Yang, J., Zhang, X., Feng, J., Leng, H., Li, S., Xiao, J., Liu, S., Xu, Z., Xu, J., Li, D., et al. (2016). The Histone Chaperone FACT Contributes to DNA Replication-Coupled Nucleosome Assembly. Cell Rep. *14*, 1128–1141.

Yeeles, J.T., Deegan, T.D., Janska, A., Early, A., and Diffley, J.F.X. (2015). Regulated eukaryotic DNA replication origin firing with purified proteins. Nature *519*, 431–435.

Yeeles, J.T.P., Janska, A., Early, A., and Diffley, J.F.X. (2016). How the Eukaryotic Replisome Achieves Rapid and Efficient DNA Replication. Mol Cell. *65*, this issue, 105–116.

Zeller, K.I., Zhao, X., Lee, C.W., Chiu, K.P., Yao, F., Yustein, J.T., Ooi, H.S., Orlov, Y.L., Shahab, A., Yong, H.C., et al. (2006). Global mapping of c-Myc binding sites and target gene networks in human B cells. Proc. Natl. Acad. Sci. USA *103*, 17834–17839.

Molecular Cell, Volume *65*

Supplemental Information

Chromatin Controls DNA Replication Origin Selection, Lagging-Strand Synthesis, and Replication Fork Rates

Christoph F. Kurat, Joseph T.P. Yeeles, Harshil Patel, Anne Early, and John F.X. Diffley

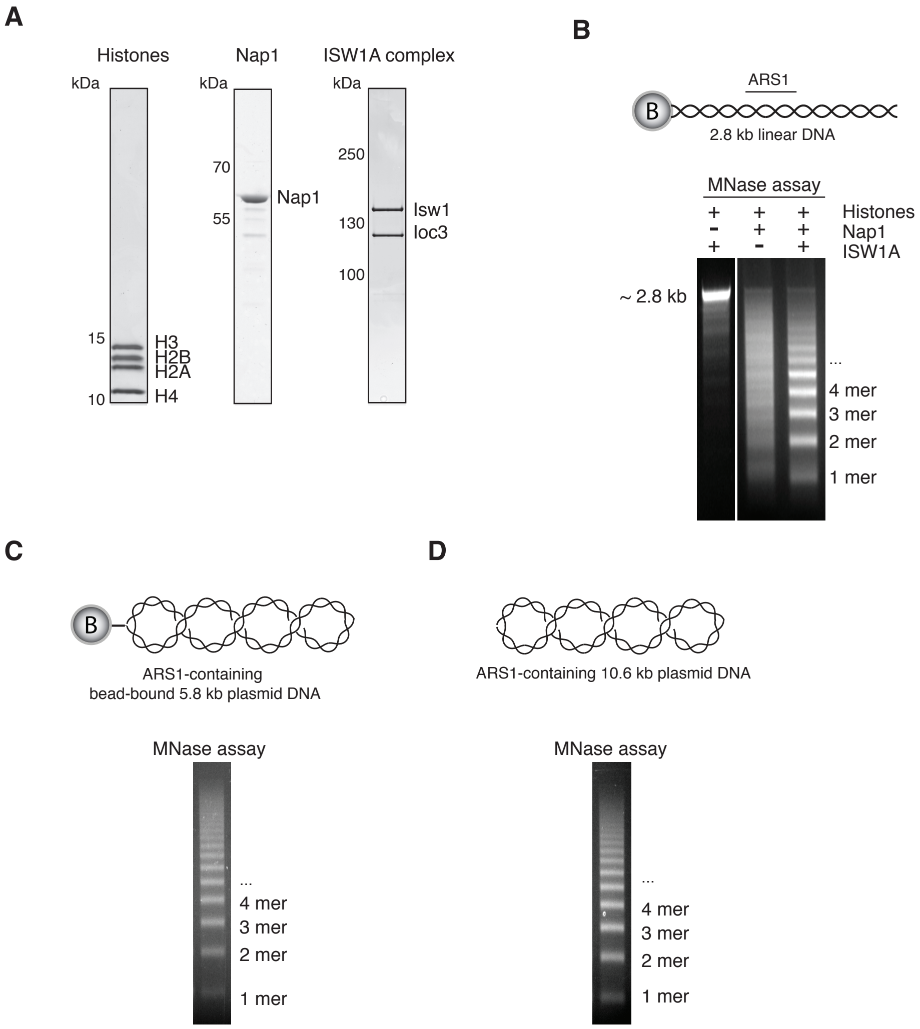
A
Histones
Nap1
ISW1A complex
kDa
15
10
H3
H2B
H2A
H4
kDa
70
55
Nap1
kDa
250
130
100
Isw1
Ioc3
B
ARS1
B
2.8 kb linear DNA
MNase assay
+ + + Histones
- + + Nap1
+ - + ISW1A
~ 2.8 kb
...
4 mer
3 mer
2 mer
1 mer
C
B
ARS1-containing
bead-bound 5.8 kb plasmid DNA
MNase assay
...
4 mer
3 mer
2 mer
1 mer
D
ARS1-containing 10.6 kb plasmid DNA
MNase assay
...
4 mer
3 mer
2 mer
1 mer

A

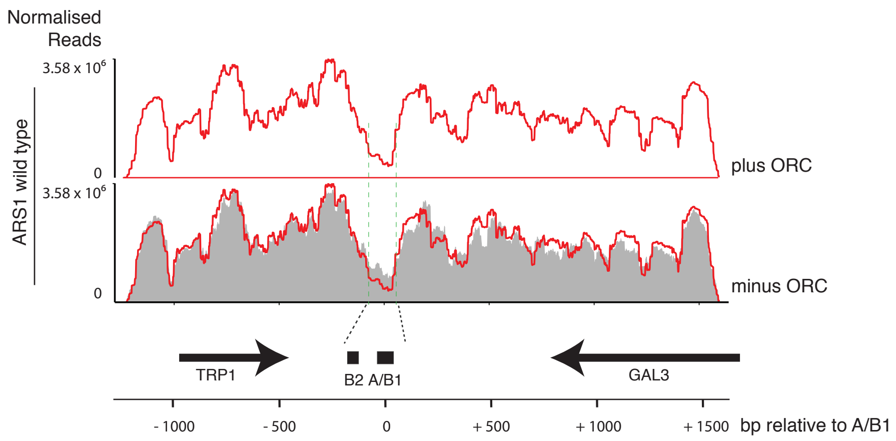
Normalised Reads
3.58 x 10^6
0
plus ORC
minus ORC
ARS1 wild type
TRP1
B2 A/B1
GAL3
- 1000
- 500
0
+ 500
+ 1000
+ 1500
bp relative to A/B1

B

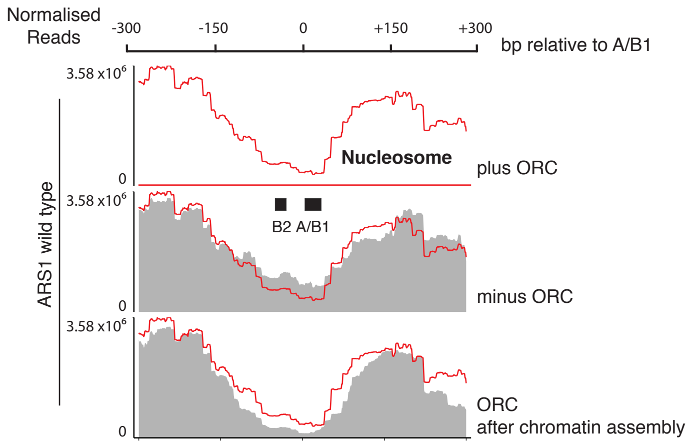
Normalised Reads
-300
-150
0
+150
+300
bp relative to A/B1
3.58 x10^6
0
Nucleosome
plus ORC
B2 A/B1
minus ORC
ORC
after chromatin assembly
ARS1 wild type

C

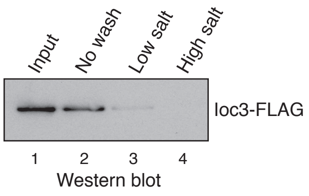
Input
No wash
Low salt
High salt
Ioc3-FLAG
1
2
3
4
Western blot

D

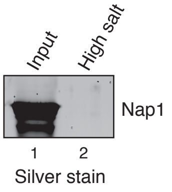
Input
High salt
Nap1
1
2
Silver stain

E

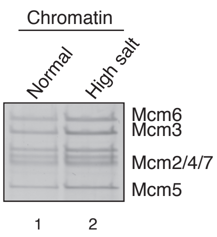
Chromatin
Normal
High salt
Mcm6
Mcm3
Mcm2/4/7
Mcm5
1
2

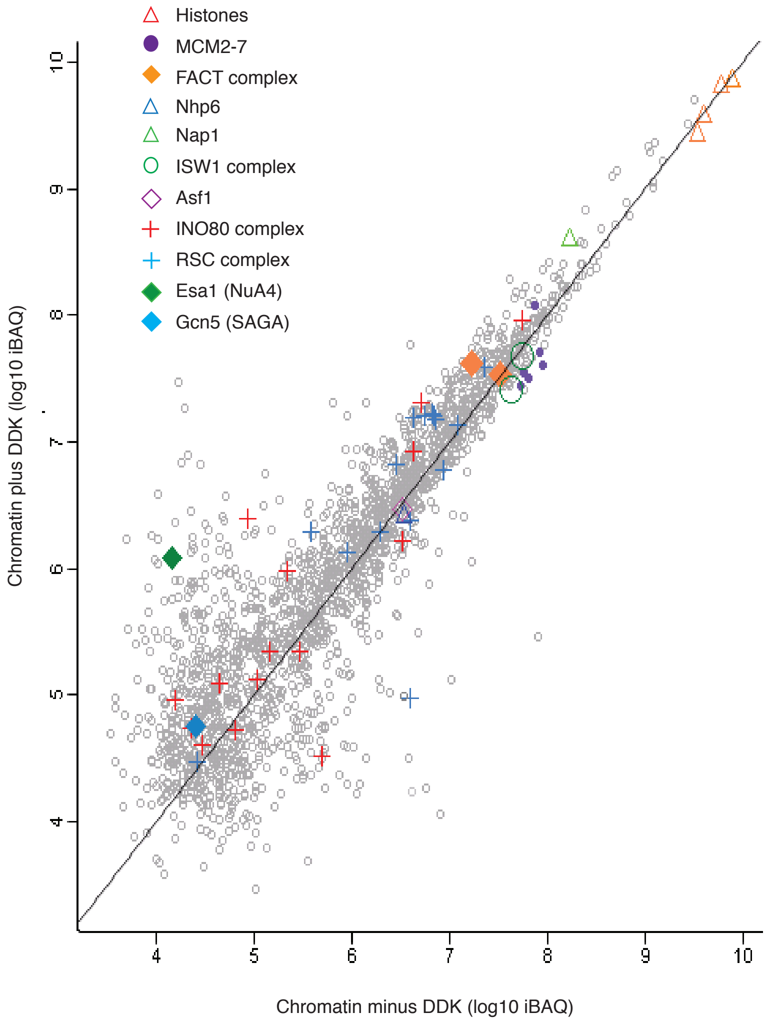
Histones
MCM2-7
FACT complex
Nhp6
Nap1
ISW1 complex
Asf1
INO80 complex
RSC complex
Esa1 (NuA4)
Gcn5 (SAGA)
Chromatin plus DDK (log10 iBAQ)
Chromatin minus DDK (log10 iBAQ)
4
5
6
7
8
9
10

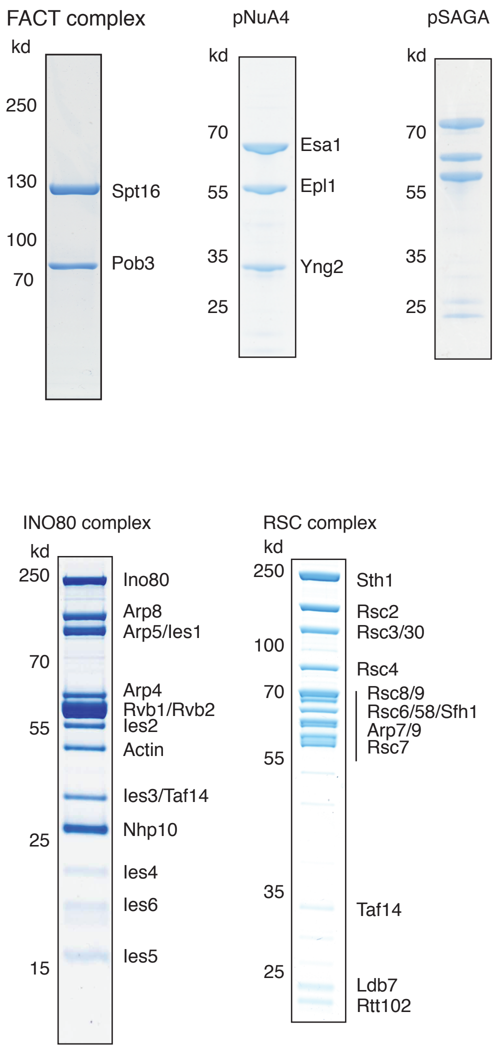
FACT complex
kd
250
130
100
70
Spt16
Pob3
pNuA4
kd
70
55
35
25
Esa1
Epl1
Yng2
pSAGA
kd
70
55
35
25
Ada3
Gcn5
Ada2
INO80 complex
kd
250
70
55
25
15
Ino80
Arp8
Arp5/Ies1
Arp4
Rvb1/Rvb2
Ies2
Actin
Ies3/Taf14
Nhp10
Ies4
Ies6
Ies5
RSC complex
kd
250
100
70
55
35
25
Sth1
Rsc2
Rsc3/30
Rsc4
Rsc8/9
Rsc6/58/Sfh1
Arp7/9
Rsc7
Taf14
Ldb7
Rtt102

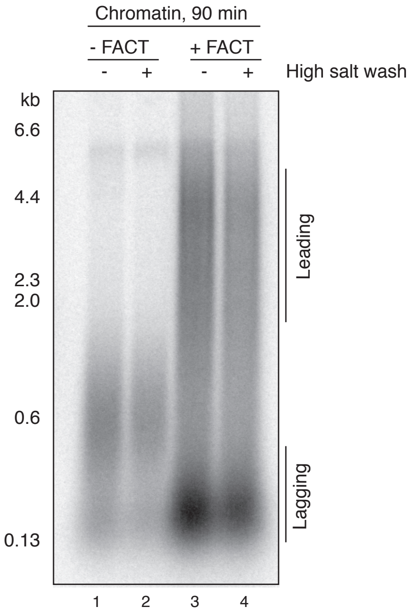
Chromatin, 90 min
- FACT
+ FACT
- + - +
High salt wash
kb
6.6
4.4
2.3
2.0
0.6
0.13
Leading
Lagging
1 2 3 4

A

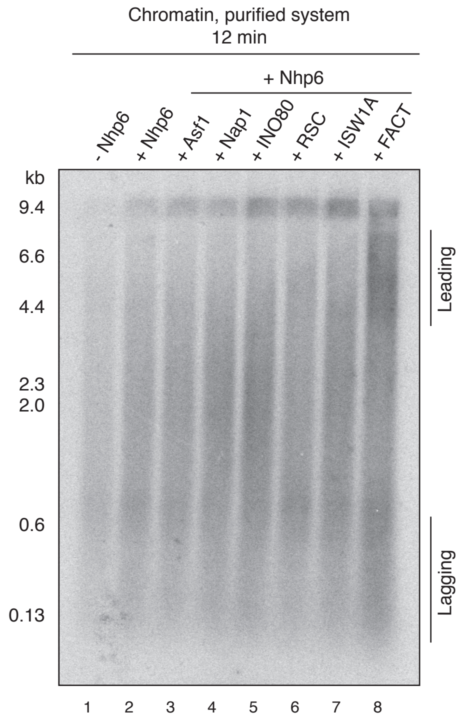
Chromatin, purified system
12 min
+ Nhp6
- Nhp6
+ Nhp6
+ Asf1
+ Nap1
+ INO80
+ RSC
+ ISW1A
+ FACT
kb
9.4
6.6
4.4
2.3
2.0
0.6
0.13
Leading
Lagging
1
2
3
4
5
6
7
8

B

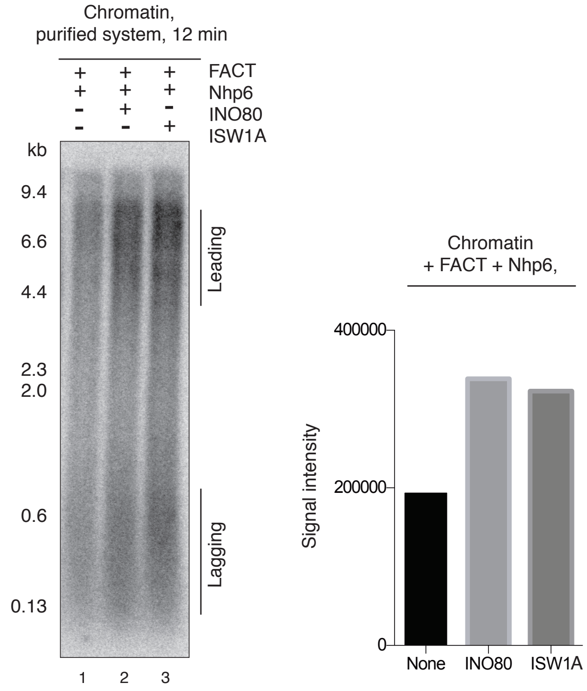
Chromatin,
purified system, 12 min
+ + + FACT
+ + + Nhp6
- + - INO80
- - + ISW1A
kb
9.4
6.6
4.4
2.3
2.0
0.6
0.13
Leading
Lagging
1
2
3
Chromatin
+ FACT + Nhp6,
Signal intensity
400000
200000
0
None
INO80
ISW1A

Kurat et al. Figure S7

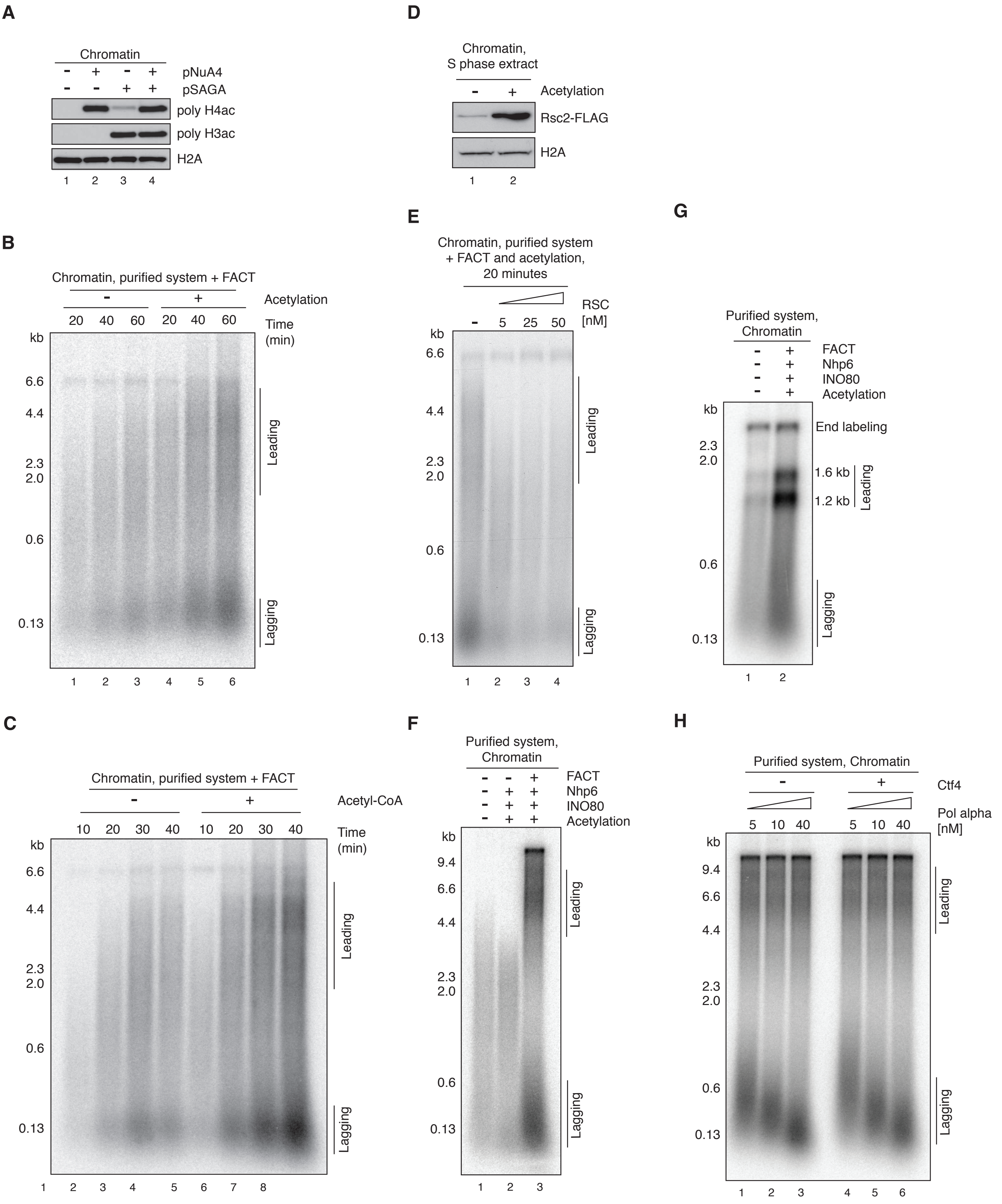

Supplemental Figures Legends

Figure S1. Related to all Figures. Chromatin assembly using different DNA substrates

(A) Purified yeast core histones, histone chaperone Nap1 and chromatin remodeler ISW1A were analyzed by SDS-PAGE and Coomassie (core histones and Nap1) or silver staining (ISW1A).

(B) Chromatin assembly on ARS1-containing 2.8 kb linear DNA coupled to paramagnetic beads. Reactions were performed in the presence or absence of Nap1 or ISW1A and treated with micrococcal nuclease (MNase) to examine efficiency of chromatin assembly.

(C) MNase assay showing chromatin assembly on a ARS1-containing 5.8 kb plasmid coupled to beads.

(D) Chromatin assembly on a 10.6 kb ARS1 plasmid in solution.

Figure S2. Related to Figure 1. Nucleosome profiles in the presence or absence of ORC

(A) Nucleosome positioning maps of the full-length ARS1-containing 2.8 kb fragment in the presence or absence of ORC are compared. Chromatin was assembled and MNase resistant nucleosomal DNA was prepared and sequenced as described in Experimental Procedures. Black bars show the relative location of the A/B1 and B2 elements. The red line is an overlay of the ARS1 wild type plus ORC experiment (first panel). Green lines indicate the relative position of the A1/B1 element of ARS1. The ARS1-flanking genes *TRP1* and *GAL3* are shown as black arrows.

(B) The top and middle panels show ARS1-adjacent nucleosomes from the experiment described in (A). The third panel shows an experiment where ORC was added after chromatin assembly. The red line is an overlay of the ARS1 wild type plus ORC experiment (first panel).

(C) Chromatin was assembled as in Figure S1 and ISW1A binding to chromatin was analyzed by immunoblotting after different washes (0.3 M K-acetate: low salt wash; 0.5 M NaCl: high salt wash). An antibody recognizing the FLAG epitope tag was used.

(D) Nap1 binding to chromatin was assessed by silver staining after a high salt wash (0.5 M NaCl).

(E) MCM loading on chromatin under standard conditions and after a high salt wash. Chromatin was assembled on 2.8 kb linear DNA in the presence of ORC and MCM was loaded as in Figure 1A (panel 1). Chromatin was assembled plus ORC and treated with a high salt wash (0.5 M NaCl) to remove ISW1 and Nap1 (Figure S5 A and B). After a low salt wash (0.3 M K-acetate), ORC, Cdc6 and MCM were added and loading reactions were performed as in Figure 1A.

Figure S3. Related to Figure 3 and Table S3. Mass spectrometry analyses of protein composition on chromatin

MCMs were loaded after chromatin assembly and either treated with DDK or left untreated before adding to an S phase extract. Histone chaperones (FACT, Nap1 and Asf1), Nhp6, chromatin remodelers (INO80, RSC and ISW1A), catalytic subunits of lysine acetyltransferases NuA4 (Esa1) and SAGA (Gcn5), the MCM complex and core histones are highlighted. Note that X- and the Y-axis are $\log_{10}$ transformed so that each unit represents a 10-fold difference in abundance. Protein quantification was performed using intensity-based quantification (iBAQ). For a complete list of all proteins identified see Table S3.

Figure S4. Related to Figure 3. Coomassie-stained SDS-PAGE analysis of multi-subunit complexes used in this study

Individual protein subunits from the polyacrylamide gel in Figure 3A are shown.

Figure S5. Related to Figure 3. Chromatin replication reaction after high salt wash

Replication reactions on chromatin in the presence or absence of the FACT complex plus and minus a high salt wash were performed as in Figure 2A.

Figure S6. Related to Figure 4. Stimulatory effect of Nhp6 on FACT

(A) Effects of histone chaperones and chromatin remodelers on chromatin replication in conjunction with Nhp6. Soluble replication reactions on chromatin were conducted as described in Figure 4A.

(B) Left panel - chromatin replication reactions showing the stimulatory effect of the INO80 and ISW1A together with FACT and Nhp6. Right panel – quantification of the replication reactions.

Figure S7. Related to Figure 4. Effects of histone acetylation and other factors on chromatin replication

(A) Chromatin was assembled on bead-coupled 5.8 kb plasmid as shown in Figure S1. Purified pNuA4, pSAGA (Figure 3A, Figure S4) and acetyl-CoA were added and acetylation was assessed using antibodies recognizing polyH3 and polyH4 acetylation. Detection of H2A was used as a loading control.

(B) Chromatin replication reactions on a bead-bound 5.8 kb plasmid were performed as shown in Figure 2A. Nucleosomes were acetylated with pNuA4 and pSAGA and replication reactions in the presence of FACT are shown at the time points indicated.

(C) Stimulatory effect of histone acetylation is dependent on acetyl-CoA. Chromatin was incubated with pSAGA and pNuA4 in the presence or absence of acetyl-CoA. Time courses of chromatin replication reactions were conducted as in (B).

(D) Recruitment of RSC in the presence and absence of histone acetylation using S phase extract. RSC recruitment was assessed using anti-FLAG antibody. Detection of H2A was used as a loading control.

(E) Chromatin replication reactions as shown in (B) with indicated amounts of RSC in the reaction.

(F) FACT is the major factor in chromatin replication. Soluble replication reactions on a 10.6 kb DNA template were performed as shown in Figure 4A with the indicated factors.

(G) Replication reactions on chromatin assembled on linear 2.8 kb DNA in the presence or absence of FACT, Nhp6, INO80 and histone acetylation. Because of the position of ARS1, two distinct leading strand products (1.2 and 1.6 kb) were observed.

(H) Ctf4 does not affect lagging strand size. Chromatin replication reactions were performed as shown in (F) in the presence or absence of Ctf4 and the indicated amounts of Pol α.

Supplemental Tables

Table S1. Related to all Figures. Yeast strains.

Strain	Genotype	Reference
yCFK1	*MATa ade2-1 ura3-1 his3-11,15 trp1-1 leu2-3,112 can1-100* *bar1::Hyg* *pep4::KanMX* *IOC3*-3xFLAG-*NAT*	This study
yCFK2	*MATa ade2-1 ura3-1 his3-11,15 trp1-1 leu2-3,112 can1-100, cdc7-4,* *his3::HIS3pRS303/SLD3-13MYC,* *trp1::TRP1pRS304/SLD2,* *leu2::LEU2pRS305/ SLD7,CDC45*	This study

	ura3::URA3pRS306/ DPB11<br>*Spt16-3xFLAG-NAT*	
yCFK3	*MATa ade2-1 ura3-1 his3-11,15 trp1-1 leu2-3,112 can1-100, cdc7-4,*<br>*his3::HIS3pRS303/SLD3-13MYC,*<br>*trp1::TRP1pRS304/SLD2,*<br>*leu2::LEU2pRS305/ SLD7,CDC45*<br>*ura3::URA3pRS306/ DPB11*<br>*Rsc2-3xFLAG-NAT*	This study
BCY211	Wittmeyer et al., 2004; Rsc2 tagged with a TAP tag at the C terminus	Kornberg lab
yAE84	*MAT**a** ade2-1 ura3-1 his3-11,15 trp1-1 leu2-3,112 can1-100*<br>*bar1::Hyg*<br>*pep4::KanMX*<br>*his3::HIS3pRS303/Ino80-TEV-3XFLAG, Nhp10*<br>*trp1::TRP1pRS304/Ies3, Ies4*<br>*leu2::LEU2pRS305/Ies5, Ies6*<br>*ura3::URA3pRS306/Ies1, Ies2*	This study
yAE85	*MATalpha ade2-1 ura3-1 his3-11,15 trp1-1 leu2-3,112 can1-100*<br>*bar1::Hyg*<br>*pep4::KanMX*<br>*his3::HIS3pRS303/Gal4, Taf14*<br>*trp1::TRP1pRS304/Rvb1, Rvb2*<br>*leu2::LEU2pRS305/Arp5, Arp8*<br>*ura3::URA3pRS306/Act1, Arp4*	This study

yAE86	*ade2-1 ura3-1 his3-11,15 trp1-1 leu2-3,112 can1-100*<br>*bar1::Hyg*<br>*pep4::KanMX*<br>*his3::HIS3pRS303/Ino80-TEV-3XFLAG, Nhp10*<br>*his3::HIS3pRS303/Gal4,Taf14*<br>*ura3::URA3pRS306/Ies1, Ies2*<br>*ura3::URA3pRS306/Act1,Arp4*<br>*leu2::LEU2pRS305/Ies5, Ies6*<br>*leu2::LEU2pRS305/Arp5, Arp8*<br>*trp1::TRP1pRS304/Ies3, Ies4*<br>*trp1::TRP1pRS304/Rvb1, Rvb2*	This study
yJH7	*MAT***a** *ade2-1 ura3-1 his3-11,15 trp1-1 leu2-3,112 can1-100*<br>*bar1::Hyg*<br>*pep4::KanMX*<br>*his3::HIS3pRS303/Asf1-CBP,Gal4*	This study

Table S2. Related to all Figures. Plasmids.

**Plasmid**	**Plasmid construction**	**Reference**
pCFK1	*NAP1* was cloned as a 5'-BamHI and 3'-NotI fragment into pGEX-6p-1 (a gift from Tim Hunt)	This study
pRS303-Ino80A	Ino80-Tev-3xFLAG-Gal-Nhp10 (starting plasmid yJF2 (for all yJF plasmids see Coster et al., 2014; Frigola et al., 2013)).<br>Ino80 was cloned with SgrAI and NotI, Nhp6 was cloned with AscI and XhoI. All subsequent genes left	This study

	to the GAL promoter were cloned with SgrAI and NotI and all genes right to GAL with AscI and XhoI.	
pRS303-Ino80B	Taf14-Gal-Gal4 (starting plasmid yJF2).	This study
pRS304-Ino80A	Ies3-Gal-Ies4 (starting plasmid pJF3).	This study
pRS304-Ino80B	Rvb2-Gal-Rvb1 (starting plasmid pJF3).	This study
pRS305-Ino80A	Ies5-Gal-Ies6 (starting plasmid pJF4)	This study
pRS305-Ino80B	Arp5-Gal-Arp8 (starting plasmid pJF4)	This study
pRS306-Ino80A	Ies1-Gal-Ies2 (starting plasmid pJF5)	This study
pRS306-Ino80B	Act1-Gal-Arp4 (starting plasmid pJF5)	This study
pRS303-Asf1	Asf1 cloned between SgrAI and NotI sites	This study
pBP83	Frigola et al., 2013	Diffley lab
pCDFduet.H2A-H2B	Kingston et al., 2011	Singleton lab
pETduet.H3-H4	Kingston et al., 2011	Singleton lab
pRJ1228-Nhp6	Ruone et al., 2003	Formosa lab
pTF175	Biswas et al., 2005	Formosa lab

pJW22	Biswas et al., 2005	Formosa lab
pST44-yAda3D2HIS-yAda3D1-Gcn5	Barrios et al., 2007	Tan lab
pST44-HISyEsa1-yEpl1D3-Yng2D2	Barrios et al., 2007	Tan lab

Table S3. Related to Figures 3 and S3. Chromatin associated proteins identified by mass spectrometry.

Supplemental Experimental Procedures

Yeast strains

Strains used in this study were constructed by transforming strain yJF1 with linearized plasmids or PCR products using standard genetic methods (details on strains and vectors see Table S 1 and 2). For generation of ISW1A expression strain, a 3xFLAG tag was chromosomally inserted at the C-terminus of Ioc3 in yJF1 using pBP83 as a template.

For INO80, codon optimized versions of the 15 subunits of INO80 were cloned in pairs into one of four versions of a yeast expression vector containing a bidirectional GAL promoter (pJF2-5, see Table S2 for details). The exception was Taf14, which was paired with Gal4. Ino80 was tagged at the C-terminus with a codon-optimized version of the 3xFLAG tag. For each of the four markers, two expression plasmids were created, which were transformed sequentially either into a MAT alpha or MAT a version of yJF1. The two final haploid strains, each expressing eight proteins, were

mated to produce the diploid INO80 expression strain. The synthetic constructs were codon optimised and synthesized as described in the accompanying manuscript.

Proteins

All proteins purified in this study, except yeast core histones and Nhp6, had affinity tags, which were used for the first step of purification. Nap1, Nhp6, pNuA4 and pSAGA were expressed in in *E. coli* BL-21 CodonPlus (DE3)-RIL. ISW1A and RSC were expressed from yeast under control of the endogenous promoters. INO80 and FACT subunits as well as Asf1 were overexpressed from a *GAL1-10* promoter in a yJF1 background. For FACT, yJF1 harbouring 2 plasmids for expression of the two subunits of FACT (see Table S 2) were grown in yeast minimal medium containing 2% sucrose as the carbon source. Expression was induced by the addition of 2 % galactose for 16 hours.

ORC, Cdc6, Cdt1-Mcm2-7, DDK, Sld2, Sld3/7, GINS, Cdc45, Dpb11, Ctf4, RPA, Topo I, Pol ε, Pol α, S-CDK, RSC, pNuA4 and pSAGA were expressed and purified as described previously (Barrios et al., 2007; Wittmeyer et al., 2004; Yeeles et al., 2015b). Mrc1, Csm3-Tof1, Pol δ, RFC and PCNA were purified as described in the accompanying manuscript.

Protein purifications

ORC, Cdc6, Cdt1-Mcm2-7, DDK, Sld2, Sld3/7, GINS, Cdc45, Dpb11, Ctf4, RPA, Topo I, Pol ε, Pol α, S-CDK, RSC, pNuA4 and pSAGA were expressed and purified as described previously (Barrios et al., 2007; Wittmeyer et al., 2004; Yeeles et al., 2015). Mrc1, Csm3-Tof1, Pol δ, RFC and PCNA were purified as described in the accompanying manuscript.

Histone purification.

Yeast core histones were expressed and purified as described previously (Kingston et al., 2011) with modifications. Pellets were slowly thawed on ice before adding 0.5 M NaCl, 20 mM Tris-HCl pH 8, 0.1 mM EDTA, 10 mM β-Mercaptoethanol and EDTA-free Protease Inhibitor Tablets (cOmplete, Roche). Cells were broken by sonication (2 minutes, 40 % output) and insoluble material was collected by centrifugation (235.000g, 4˚C, 45 minutes). Supernatant was passed over a 5ml HiTrap Heparin column (GE Healthcare) and eluted with 20 column volumes (CV) of a 0 M to 2 M NaCl gradient (20 mM Tris-HCl pH 8, 0.1 mM EDTA, 10 mM β-Mercaptoethanol, no protease inhibitors). Peak fractions were collected, concentrated and passed over a Superdex 200 column (GE Healthcare) using 2 M NaCl buffer ((20 mM Tris-HCl pH 8, 0.1 mM EDTA, 10 mM β-Mercaptoethanol, no protease inhibitors). Peak fractions were pooled and concentrated.

Nap1 purification.

Cell pellets were slowly thawed on ice before adding an equal amount of Nap1 lysis buffer (50 mM $KxPO_4$ pH 7.6, 150 mM KOAc, 5 mM $MgCl_2$, 1% Triton, 1 mM DTT and EDTA-free Protease Inhibitor Tablets (cOmplete, Roche, 1 tablet per 50 ml lysis buffer)). Cells were broken and centrifuged as described for histone purification. 1 ml glutathione agarose slurry was washed with Nap1 lysis buffer, before adding the cleared lysate for 2 hours at 4 ˚C followed by transfer into a disposable column. Beads were washed extensively with Nap1 lysis buffer without protease inhibitors. An equal amount of lysis buffer was added to beads to generate 50% slurry. To cleave Nap1 from GST, 50 ml Prescission protease (GE Healthcare) was added for 2 hours at 4 ˚C. Eluate was collected, dialyzed into 20 mM Tris-HCl ph 7.5, 100 mM NaCl, 0.5 mM EDTA, 10 % glycerol, 1 mM DTT and 0.1 mM PMSF) and Nap1 was further purified using a 1 ml MonoQ column (GE Healthcare). Nap1 was eluted using a 20

CV gradient from 0.1 M to 1 M NaCl (20 mM Tris-HCl ph 7.5, 100 mM NaCl, 0.5 mM EDTA, 10 % glycerol and 1 mM DTT). Peak fractions were pooled and concentrated.

ISW1A purification.

ISW1A was purified as previously described with modifications (Vary et al., 2004). Cell pellets were resuspended in an equal volume of lysis buffer (25 mM HEPES pH 7.6, 0.1 mM EDTA, 2 mM $MgCl_2$, 20 % glycerol, 0.02 % NP40, 1 mM DTT and 300 mM KCl) with 1x protease inhibitors (0.3 mM PMSF, 7.5 mM benzamidine, 0.5 mM AEBSF, 1 mM pepstatin A, 1 mg/ml aprotinin (Sigma) and 1 mM leupeptin (Merck)). Insoluble material was cleared as above (235.000g, 4˚C, 45 minutes) and Ioc3-FLAG was bound to pre-washed anti-FLAG M2 affinity gel (Sigma) in batch 1 hour at 4 ˚C. After transferring into a disposable column, beads were washed with 20 CV of lysis buffer containing 300 mM KCl followed by 20 CV of lysis buffer containing 100 mM KCl. ISW1 was eluted in 1 CV of lysis buffer containing 100 mM KCl with 0.5 mg/ml 3x FLAG peptide, followed by 2 CV of lysis buffer containing 100 mM KCl with 0.25 mg/ml 3x FLAG peptide.

The eluates were pooled and applied to a 1 ml MonoQ column. ISW1 was eluted with a 15 CV gradient from 200 mM KCl to 600 mM KCl in lysis buffer. Peak fractions were pooled and concentrated.

Asf1 purification.

For purification of Asf1-CBP, frozen cell pellets were thawed slowly on ice. An equal amount of CBP lysis buffer (30 mM HEPES pH 7.6, 100 mM KCl, 10% glycerol, 5 mM DTT, 0.1% NP-40 and EDTA-free Protease Inhibitor Tablets) was added and insoluble material was removed by centrifugation (235.000 g, 4˚C, 1 hour). Supernatant was added to pre-washed Calmodulin affinity resin (Agilent) together with $CaCl_2$ to 2 mM and rotated for 2 hours at 4 ˚C. After transferring into a 20 ml disposable column, beads were washed extensively with wash buffer (30 mM

HEPES pH 7.6, 100 mM KCl, 10% glycerol, 5 mM DTT, 0.1% NP-40 and 2 mM $CaCl_2$). Asf1-CBP was eluted with 10x 1ml buffer containing 30 mM HEPES pH 7.6, 100 mM KCl, 10% glycerol, 5 mM DTT, 0.1% NP-40, 2 mM EDTA and 2 mM EGTA. The eluates were pooled and applied to a 1 ml MonoQ column. Asf1-CBP was eluted with a 30 CV gradient from 0 to 1 M KCl (0/1 M KCl, 30 mM HEPES pH 7.6, 10% glycerol, 1 mM DTT and 0.5 mM EDTA). Peak fractions were pooled and dialyzed into buffer containing 30 mM HEPES pH 7.6, 100 mM KCl, 10% glycerol, 5 mM DTT and 0.1% NP-40.

FACT purification.

FACT was purified as previously described (Biswas et al., 2005) with modifications. 16 g of cell pellets were resuspendend in 8 ml buffer containing 20 mM Tris-HCl pH 8 and 5 mM Imidazole and dropped into liquid nitrogen to make popcorn. Popcorn was then manually ground in a pre-cooled mortar with constant addition of liquid nitrogen. Cell powder was collected, thawed on ice (24 ml) and 16 ml buffer (20 mM Tris-HCl pH 8, 5 mM Imidazole, 0.5 mM NaCl and EDTA-free Protease Inhibitor Tablet) was added to give a total volume of 40ml. Because there was no NaCl in the cell-resuspension buffer, 2.4 ml of 5 M NaCl was added to give a final concentration of 0.5 M NaCl. After a first centrifugation step (12.000 g, 10 minutes, 4 ˚C), supernatant was collected and insoluble material was removed by a second centrifugation step (235.000 g, 4˚C, 30 minutes). The supernatant was added to 8 ml of pre-washed Talon Metal Affinity resin (Clontech). Beads were gently rotated in batch for 75 minutes at 4˚C. Beads were then washed with 2x 50 ml buffer (20 mM Tris-HCl pH 8, 5 mM Imidazole and 0.5 mM NaCl) before transferring into a 20 ml disposable column. Beads were washed with additional 40 ml buffer (20 mM Tris-HCl pH 8, 0.5 M NaCl and 20 mM Imidazole) before FACT was eluted with 8 x 1ml buffer E1 (20 mM Tris-HCl pH 8, 0.5 M NaCl and 100 mM Imidazole), 2 x 1ml buffer E2 (20 mM Tris-HCl pH 8, 0.5 M NaCl and 500 mM Imidazole) and 2 x 1 ml buffer E3 (20 mM

Tris-HCl pH 8, 0.5 M NaCl and 1 M Imidazole). Desired fractions were concentrated and loaded onto Superdex S200 column, which was equilibrated with buffer containing 20 mM Tris-HCl pH 7.5, 200 mM NaCl, 10 % glycerol and 1 mM β-Mercaptoethanol.

Nhp6 purification.

Nhp6 was purified as previously described with modifications (Ruone et al., 2003). Cell pellets were thawed on ice and an equal amount of lysis buffer was added (20 mM Tris-HCl pH 7.5, 500 mM NaCl, 2 mM EDTA, 10% glycerol and 1 mM β-Mercaptoethanol). Cells were lysed by sonication (2 minutes, 40 % output) and supernatant was collected by centrifugation (12.000 g, 10 minutes, 4 ˚C) before pouring the supernatant into an Erlenmeyer flask with a magnetic stir bar. The volume of 50% trichloroacetic acid (TCA) to add was determined by multiplying the volume of the supernatant by 0.0417 (2 % final concentration of TCA). TCA was added slowly while stirring to prevent precipitation of Nhp6 (unlike Nhp6, most other proteins precipitate at 2 % TCA). Stirring was continued for 30 minutes at 4 ˚C. The solution was distributed to polypropylene centrifuge tubes and centrifuged (39.000 g, 30 minutes, 4˚C). The supernatant was pooled into a graduated cylinder to get an accurate measurement of the volume before pouring into an Erlenmeyer flask with a magnetic stir bar. TCA was added to a final concentration of 10 % to precipitate Nhp6. Stirring was continued for 30 minutes at 4 ˚C. Precipitated material was collected by a centrifugation step (39.000 g, 30 minutes, 4˚C). The pellet was washed with acetone, vortexed and centrifuged for 5 minutes (30.000 g, 4 ˚C). Pellets were dried under vacuum and dissolved in buffer containing 20 mM Tris-HCl pH 7.5, 300 mM NaCl, 1 mM EDTA, 10 % glycerol and 1 mM β-Mercaptoethanol and dialyzed against the same buffer twice for at least 3 hours each time. After filtering through a 0.2mM syringe filter, the dialyzed sample was applied to a MonoS column

(GE Healthcare) and eluted with a 35 ml gradient from 300 to 1400 mM NaCl (20 mM Tris-HCl pH 7.5, 1 mM EDTA, 10 % glycerol and 1 mM β-Mercaptoethanol). Desired fractions were pooled and dialyzed against 600 ml of buffer containing 200 mM KOAc, 1 mM β-Mercaptoethanol, 25 mM HEPES pH 7.6 and 10 % glycerol.

INO80 purification.

INO80 was purified as previously described with modifications (Shen, 2004). Pellets were thawed slowly on ice before adding an equal amount of lysis buffer (25 mM HEPES pH 7.6, 500 mM KCl, 10 % glycerol, 0.05% NP-40, 1 mM EDTA, 1 mM DTT, 4 mM $MgCl_2$ and protease inhibitors (0.3 mM PMSF, 7.5 mM benzamidine, 0.5 mM AEBSF, 1 mM leupeptin, 1 mM pepstatin A and 1 mg/ml aprotinin)). Soluble material was collected by centrifugation (80.000 g, 2 hours, 4˚C). An equal amount of lysis buffer plus protease inhibitors was added to the supernatant and applied to anti-FLAG M2 affinity gel (Sigma) in batch for 1 hour at 4 ˚C. Beads were washed extensively with 25 mM HEPES pH 7.6, 500 mM KCl, 10 % glycerol, 0.05% NP-40, 1 mM EDTA, 1 mM DTT, 4 mM $MgCl_2$, and then washed with 25 mM HEPES pH 7.6, 200 mM KCl, 10 % glycerol, 0.05% NP-40, 1 mM EDTA, 1 mM DTT, 4 mM $MgCl_2$. INO80 was eluted by 1 CV of the same buffer with 0.5 mg/ml 3x FLAG peptide, followed by 2 CV of buffer with 0.25 mg/ml 3x FLAG peptide. The elutate was then dialyzed into 200 mM KOAc, 25 mM Tris-HCl pH 7.2, 10 % glycerol, 1 mM DTT, 0.05 % NP-40 and 4 mM $MgCl_2$ before applying to a MonoQ column, which was equilibrated in a buffer containing 100 mM KCl, 25 mM Tris-HCl pH 7.2, 10 % glycerol, 1 mM DTT, 0.05 % NP-40 and 4 mM $MgCl_2$. After running a gradient from 100 mM to 300 mM KCl for 10 CV, INO80 was stepped-off with a short gradient of 2 CV from 300 to 600 mM KCl.

INO80 was then dialyzed into buffer containing 100 mM NaCl, 25 mM Tris-HCl pH 7.2, 1 mM DTT, 10 % glycerol and 1 mM EDTA.

Chromatin assembly and MCM loading on bead-coupled linear DNA

Chromatin assembly was carried out as described previously (Vary et al., 2004). For chromatin assembly and MCM loading on bead-coupled linear DNA, a 2.8 kb fragment of yeast DNA containing ARS1 wild type and ars1 A^-B2^- mutant was prepared as described (Yeeles et al., 2015). Highly saturated chromatin was assembled in buffer containing 10 mM HEPES pH 7.6, 50 mM KCl, 5 mM $MgCl_2$, 0.5 mM EGTA, 10 % glycerol and 0.1 mg/ml BSA. Yeast histones (370 nM), Nap1 (3.5 μM) and ISW1 (7 nM) were combined and left on ice for 30 minutes. Then, creatine phosphate (40 mM), ATP (3mM), creatine phosphate kinase (0.6 ml of a 14 mg/ml stock solution) and bead coupled DNA (500 ng) were added and incubated at 30 ˚C with shaking (1250 rpm). After 10 minutes, ORC (20 nM, unless stated otherwise) was added and the reaction was incubated for additional 3 hours and 50 minutes. Naked DNA was treated exactly as chromatin, without addition of histones, Nap1 and ISW1. To get origin specificity, beads were washed twice with 200 ml of low salt buffer (45 mM HEPES-KOH pH 7.6, 5 mM $Mg(OAc)_2$, 0.02 % NP-40, 10 % glycerol and 0.3 M KOAc). Loading buffer (25 mM HEPES-KOH pH 7.6, 10 mM $Mg(OAc)_2$, 90 mM KOAc, 1 mM DTT, 0.1 % NP-40 and 5 % glycerol), Cdt1-Mcm2-7 (50 nM), Cdc6 (80 nM) and ATP (2 mM) were added to the reaction and incubated for 30 minutes at 30 ˚C with shaking. Beads were washed with high (as low salt buffer, just 0.5 M NaCl instead of 0.3 M KOAc) or low salt buffer, naked DNA and chromatin bound fractions were released from beads with addition of MNase (NEB, 2000 units, 37 ˚C, 5 minutes) and analysed by immunoblotting or silver staining.

Nucleosome positioning

Chromatin was assembled plus and minus ORC (30 nM). MNase sequencing was carried out on the Illumina HiSeq 2500 platform and typically generated ~40 million 51bp paired-end reads per sample. Alignments were performed using bwa (version

0.7.7-r441; (Li and Durbin, 2009)) with the following parameters; -l=51 -k=2 -n=2. Alignments were filtered to include concordantly mapped read pairs with an insert size between 110-160bp. Per base sample coverage was calculated by treating each pair of reads as a single fragment, including the inferred insert portion. A median mapped library size of 40 million read pairs was used to normalise the coverage for cross-sample comparison.

S phase extracts

Spt16-FLAG (FACT complex) was depleted from an yCFK2 extract by 3 rounds (60 minutes each) of incubation at 4˚C with a 20 % volume of anti-FLAG M2 magnetic beads (Sigma). A mock sample was incubated for the same time with shaking at 4 ˚C. Total protein concentrations of both samples were measured using Bradford and the mock sample was diluted using 50 mM HEPES-KOH pH 7.6, 5 mM $Mg(OAc)_2$, 300 mM K-glutamate, 1 mM EDTA, 1 mM EGTA, 10 % glycerol and 3 mM DTT to adjust total protein concentrations. Recruitment of RSC to chromatin was detected by incubation with an S phase extract harbouring Rsc2-FLAG (yCFK3). Chromatin was acetylated as described below.

CMG recruitment assays

For CMG recruitment reactions, ARS1 containing circular plasmids coupled to beads were generated as described previously (Yeeles et al., 2015a). Chromatin assembly and MCM loading were executed as described before using 500 ng bead-coupled DNA. On bead bound circular plasmids, MCM loading on chromatin was less than on naked DNA, presumably because MCM loading on naked DNA was not origin specific. Therefore MCM levels had to be adjusted. For all subsequent experiments (recruitment and replication assays) involving naked DNA 15 nM Cdt1-Mcm2-7 (instead of 50 nM for chromatin) were used for the loading step. Loaded MCMs were then incubated with or without 100 nM DDK at 25 ˚C for 30 minutes. For reactions

using S phase extract, 10 ml of loaded MCMs, 10 ml of S phase extract and 15 ml of buffer containing 13 mM $MgCl_2$, 60 mM HEPES-KOH pH 7.6, 7 mM ATP, 4 nM DTT, 60 mM creatine phosphate, 1 ml creatine phospho kinase (14 mg/ml stock solution), 40 μM dATP, dCTP, dGTP, dTTP and 100 μM CTP, GTP, UTP were added. Reactions were incubated at 30 ˚C for 30 minutes with shaking. Beads were washed twice in TE buffer pH 8 and then twice with high salt buffer containing 45 mM HEPES-KOH pH 7.6, 5 mM $Mg(OAc)_2$, 0.02 % NP-40, 10 % glycerol and 0.3 M KCl. Naked DNA and chromatin bound fractions were released from beads by the addition of MNase and analysed by immunoblotting. For recruitment assays using purified proteins, MCM loading and DDK treatment were executed as described above. Beads were then collected, washed with low salt buffer and reactions were started by adding a master mix containing 40 mM HEPES-KOH pH 7.8, 250 mM K-glutamate, 5 % glycerol, 5 mM ATP, 10 mM $Mg(OAc)_2$, 2 mM DTT, 400 mg/ml BSA, 0.02 % NP-40, 40 nM Sld3/7, 60 nM Sld2, 40 nM Cdc45, 30 nM Dpb11, 30 nM Pol ε, 210 nM GINS, 50 nM S-CDK and 5 nM Mcm10. Reactions were incubated at 30 ˚C with shaking for 10 minutes before treated as described above. CMG recruitment was assessed by immunoblotting.

Replication assays on bead bound plasmid DNA templates

Replication assays using bead coupled circular ARS1 plasmid (Yeeles et al., 2015a) and S phase extract were identical to CMG recruitment assays with the exception of adding 80 nM of [a-^{32}P] -dCTP to the reaction. Replication reactions were terminated by removing the supernatant and washing the twice beads in TE buffer pH 8. Beads were resuspended in 5 mM EDTA, and NaOH (50 mM) and sucrose (1 % w/v) were then added. Beads were incubated at 30 ˚C for 30 minutes with shaking. Replication products were separated through 0.8 % alkaline agarose gels in 30 mM NaOH and 2 mM EDTA for 16 hours at 26 V. After fixing with 5 % cold trichloroacetic acid for 20

minutes twice, gels were dried onto Whatman papers and autoradiographed with Amersham Hyperfilm-MP (GE Healthcare) or scanned using a Typhoon phosphoimager (GE Healthcare). Quantification was executed using ImageQuant software.

For replication reactions using bead bound plasmid DNA and purified proteins, chromatin assembly, MCM loading and DDK phosphorylation were conducted as described above. After collecting the beads and removing the supernatant, beads were washed twice in buffer containing 45 mM HEPES-KOH pH 7.6, 5 mM $Mg(OAc)_2$, 0.02 % NP-40, 10 % glycerol and 0.3 M KOAc. Replication was initiated by adding a replication mix containing 25 mM Hepes KOH pH 7.6, 200 mM K-glutamate, 10 mM $Mg(OAc)_2$, 100 μg/ml BSA, 1 mM DTT, 0.01% NP-40, 3 mM ATP, 200 μM CTP, GTP, UTP, 40 μM dCTP, dGTP, dATP, dTTP, 60 nM [α^{32}P]-dCTP, 20 nM Ctf4, 30 nM Dpb11, 210 nM GINS, 40 nM Cdc45, 20 nM Pol ε, 5 nM Mcm10, 50 nM Sld2, 25 nM Sld3/7, 20 nM Pol α, 10 nM Topo I, 100 nM RPA, 20 nM S-CDK, 20 nM Csm3/Tof1, 10 nM Mrc1, 10 nM Pol δ, 20 nM RFC and 20 nM PCNA. For chromatin replication reactions, FACT was added at 40 nM unless stated otherwise. For chromatin replication reactions using acetylated nucleosomes as a template, 20 mM acetyl CoA, 300 nM pNuA4 and 300 nM SAGA were added after the DDK step and incubated for 30 minutes at 30 ˚C with shaking.

Samples were treated and analysed as described above.

Soluble replication assays

All reactions were incubated at 30 ˚C without shaking. ARS1 containing 10.6 kb plasmids were generated as described in the accompanying manuscript. Chromatin assembly reactions in the presence of ORC were performed as described before with 1.3 nM plasmid DNA as the template and in a buffer containing 25 mM HEPES-KOH pH 7.6, 10 mM $Mg(OAc)_2$, 100 mM KOAc, 0.1 % NP-40, 5 % glycerol and 0.1 mg/ml

BSA. Prior to MCM loading, ORC containing chromatinised circles were then put over a gel filtration column (MicroSpin S-400 HR Column, GE Healthcare), which was equilibrated three times with 250 ml buffer containing 100 mM K-glutamate, 25 mM HEPES, 10 mM Mg(OAc)$_2$, 0.02 % NP-40 and 1 mM DTT. For MCM loading, 2 mM ATP, 50 mM Cdt1-Mcm2-7 and 80 mM Cdc6 were added and incubated at 30 ˚C for 30 minutes. After a DDK step (50 nM, 30 ˚C for 30 minutes), chromatin was acetylated if indicated (20 mM acetyl CoA, 300 nM pNuA4 and 300 nM SAGA; 30 ˚C for 30 minutes). For replication, 10 ml of a master-mix containing 300 mM K-glutamate, 25 mM Hepes KOH pH 7.6, 10 mM Mg(OAc)$_2$, 100 μg/ml BSA, 1 mM DTT, 0.01% NP-40, 3 mM ATP, 400 μM CTP, GTP, UTP, 80 μM dCTP, dGTP, dATP, dTTP, 60 nM [α^{32}P]-dCTP, 20 nM Ctf4, 30 nM Dpb11, 210 nM GINS, 40 nM Cdc45, 20 nM Pol ε, 5 nM Mcm10, 50 nM Sld2, 25 nM Sld3/7, 20 nM Pol α (except Pol α titration experiments), 10 nM Topo I, 100 nM RPA, 20 nM S-CDK, 20 nM Csm3/Tof1, 10 nM Mrc1, 10 nM Pol δ, 20 nM RFC and 20 nM PCNA was added to 10 ml of a MCM loading reaction. This resulted in final concentrations of 200 mM K-glutamate, 200 μM CTP, GTP, UTP and 40 μM dCTP, dGTP, dATP, dTTP. For chromatin replication reactions unless stated otherwise, FACT was added at 40 nM and Nhp6 was added at 400 nM. When indicated, 40 nM INO80, 40 nM ISW1A, 30 nM Asf1, 30 nM Nap1 and 40 nM RSC were added. To stop the reactions, 30 mM ETDA was added and unincorporated nucleotides were removed using Illustra MicroSpin G-50 columns (GE Healthcare). Samples were separated through 0.8% alkaline agarose gels as described before.

For soluble reactions using naked DNA, MCMs were loaded in a buffer containing 1.3 nM ARS1 10 kb plasmid DNA, 15 nM Cdt1-Mcm2-7, 20 nM ORC, 80 nM Cdc6, 100 mM K-glutamate, 25 mM HEPES-KOH pH 7.6, 10 mM Mg(OAc)$_2$, 5 mM ATP, 0.02 % NP-40 and 1 mM DTT. Reactions were incubated for 30 minutes at 30 ˚C before DDK (50 nM) was added and incubated for another 30 minutes at 30˚C.

Replication reactions were started by adding the same master-mix as described for soluble reactions on chromatin, the only exception being 400 mM K-glutamate in the buffer, which resulted in a final concentration of 250 mM after combining with loaded MCMs. When indicated, 40 nM FACT and 400 nM Nhp6 were added to the reaction. Reactions were treated as described above. For MNase assays in the presence of chromatin modifying proteins, chromatin was made as described, purified over a gel filtration column. When indicated, chromatin was acetylated as described and incubated with indicated factors for 7 minutes at 30 ˚C. MNase assay was executed as described before.

Data analyses

All gels were scanned using a typhoon phosphorimager. For pulse-chase experiments the positions of the peaks were assigned manually. To obtain replication rates, data were processed as described in the accompanying manuscript.

Antibodies

Spt16-FLAG and Ioc3-FLAG were visualised using anti-FLAG M2 peroxidase (Sigma). Psf1 antibody was a gift from K. Labib. Antibody against Cdc45 was described (On et al., 2014). Anti-Mcm7 was from Santa Cruz (yN-19, sc 6688) and anti-ORC6 from S. Bell (SB49). To detect acetylation, anti-acetyl histone H3 and H4 were used respectively (Millipore 06-599 and 06-866) were used. Anti H2A was used as a loading control (Active Motif, 39235).

DNA primers

Ioc3-F:

ACGACAATGATTCTTCTTTTGATGATGGTAGAGTTAAAAGGCAGCGCACTCTGG

AAGTGCTGTTTCAGGGCCCGCGTACGCTGCAGGTCGAC

Ioc3-R:

GCCTGTAAGGAGTTTCACAATCTTCACGTTCGTTGAAAGCTAGTTGTCTAATCGA

TGAATTCGAGCTCG

Spt16-F:

AATTAGAGAAAAAGGCTGCTAGGGCTGATAGGGGTGCAAACTTTAGAGATCGTA

CGCTGCAGGTCGAC

Spt16-R:

TTCTGTCAGATCAAGGTCTTGCTGGTGAAACCCAGTAAGTGTTATAAAGTATCGA

TGAATTCGAGCTCG

Rsc2-F:

AGTTCACGGCGCACAGACTCTCTATGCTGCGGCCTCCTTCGTCGTCTTCACGTA

CGCTGCAGGTCGAC

Rsc2-R:

ATGCGCAATGGGAAGATATTATGCTGCCATTGCTTTTACAATAAAGGTGAATCGA

TGAATTCGAGCTCG

Nap1_BamHI-F: CGATGGATCCTCAGACCCTATCAGAACGAAACC

Nap1_NotI-R: CGATGCGGCCGCTTATGACTGCTTGCATTCAGGAG

Supplemental References

Barrios, A., Selleck, W., Hnatkovich, B., Kramer, R., Sermwittayawong, D., and Tan, S. (2007). Expression and purification of recombinant yeast Ada2/Ada3/Gcn5 and Piccolo NuA4 histone acetyltransferase complexes. Methods *41*, 271-277.

Biswas, D., Yu, Y., Prall, M., Formosa, T., and Stillman, D.J. (2005). The yeast FACT complex has a role in transcriptional initiation. Mol Cell Biol *25*, 5812-5822.

Kingston, I.J., Yung, J.S., and Singleton, M.R. (2011). Biophysical characterization of the centromere-specific nucleosome from budding yeast. J Biol Chem *286*, 4021-4026.

Li, H., and Durbin, R. (2009). Fast and accurate short read alignment with Burrows-Wheeler transform. Bioinformatics *25*, 1754-1760.

Ruone, S., Rhoades, A.R., and Formosa, T. (2003). Multiple Nhp6 molecules are required to recruit Spt16-Pob3 to form yFACT complexes and to reorganize nucleosomes. J Biol Chem *278*, 45288-45295.

Shen, X. (2004). Preparation and analysis of the INO80 complex. Methods Enzymol *377*, 401-412.

Vary, J.C., Jr., Fazzio, T.G., and Tsukiyama, T. (2004). Assembly of yeast chromatin using ISWI complexes. Methods Enzymol *375*, 88-102.
Wittmeyer, J., Saha, A., and Cairns, B. (2004). DNA translocation and nucleosome remodeling assays by the RSC chromatin remodeling complex. Methods Enzymol *377*, 322-343.
Yeeles, J.T., Deegan, T.D., Janska, A., Early, A., and Diffley, J.F. (2015). Regulated eukaryotic DNA replication origin firing with purified proteins. Nature *519*, 431-435.
